# Synthesis of N-, O-, and S-heterocycles from aryl/alkyl alkynyl aldehydes

**DOI:** 10.1039/d3ra01778h

**Published:** 2023-05-09

**Authors:** Fatemeh Doraghi, Farid Mohaghegh, Omid Hosseinchi Qareaghaj, Bagher Larijani, Mohammad Mahdavi

**Affiliations:** a Endocrinology and Metabolism Research Center, Endocrinology and Metabolism Clinical Sciences Institute, Tehran University of Medical Sciences Tehran Iran momahdavi@tums.ac.ir; b School of Chemistry, College of Science, University of Tehran Tehran Iran; c Chemistry and Chemical Engineering Research Center of Iran. Tehran Iran

## Abstract

In the field of heterocyclic synthesis, alkynyl aldehydes serve as privileged reagents for cyclization reactions with other organic compounds to construct a broad spectrum of N-, O-, and S-heterocycles. Due to the immense application of heterocyclic molecules in pharmaceuticals, natural products, and material chemistry, the synthesis of such scaffolds has received wide attention. The transformations occurred under metal-catalyzed, metal-free-promoted, and visible-light-mediated systems. The present review article highlights the progress made in this field over the past two decades.

## Abstract 2

Preparation of many kinds of N-, O-, and S-heterocycles from aryl/alkyl alkynyl aldehydes were investigated in recent years. In this review, the synthesis of bioactive heterocycles using by substituted alkynyl aldehydes is highlighted.

## Graphical abstract

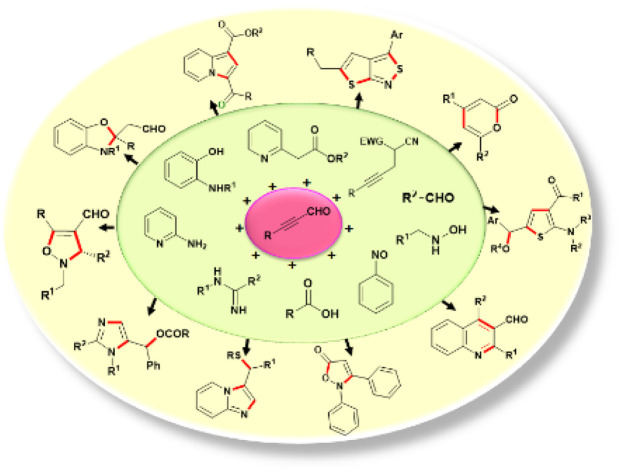

## 1. Introduction

**Scheme 1:**
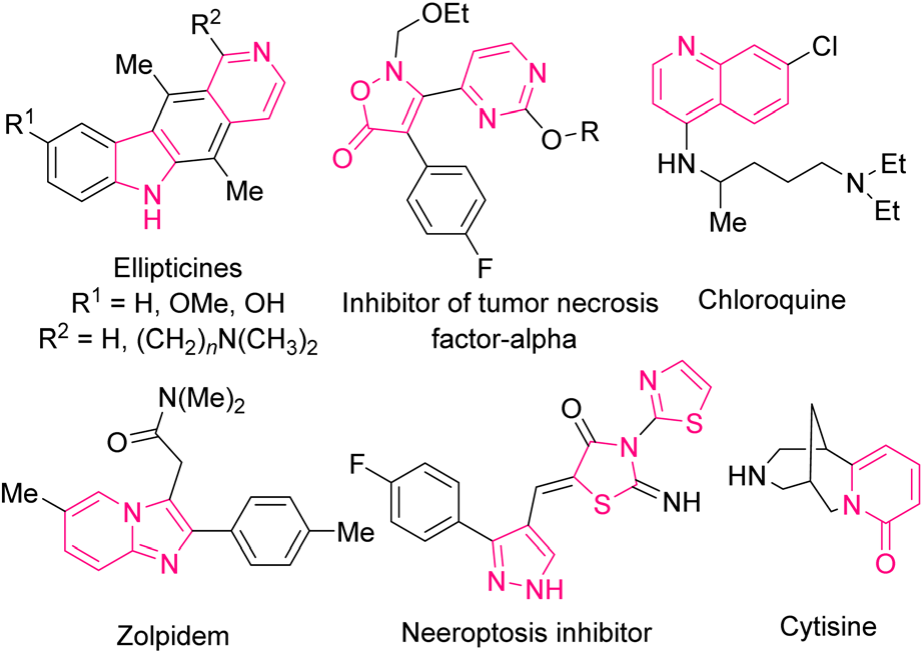
Some biologically heterocyclic compounds.

The alkynyl group is found in various natural products, bioactive molecules, and pharmaceutical agents^1^ and also, regards as a versatile and valuable building block for the organic transformations and manipulations in organic chemistry.^2^ If there is a carbonyl functionality in conjugation with a C–C triple bond, it can expand the application and synthetic significance of the compound. In this regard, a substituted alkynyl aldehyde as an end-product or an intermediate is widely used in organic synthesis, including 1,4-addition, or cyclization reactions which result in the preparation of imidazoles, imidazo[1,2-*a*]pyridines, oxazoles, thiazoles, triazoles, indolizines, pyrimidines, thiophenes, pyrroles, pyridines, pyranones, furans, quinolines, thiopyranes, *etc.* Due to the immense presence of N-, O-, and S-heterocyclic molecules in medicinal, and material science, the synthesis of such compounds has attracted extensive attention from chemists.^3^ A variety of drugs, drug candidates, and natural products consist of one N-, O-, and S-heterocycle ring or two bound, linked, spaced, or fused heterocycles. Some of these biologically heterocyclic compounds are depicted in Scheme 1. These structural motifs have emerged as novel targets in modern organic chemistry because of their hybrid features and improved potential applications in comparison with their discrete components in many areas.

Over the years, many approaches have been described for the preparation of heterocycles from alkynyl moieties and different kinds of chemical compounds.

This review covers the developments in the last two decades in the construction of valuable N-, O-, and S-heterocyclic compounds from aryl/alkyl/heteroaryl alkynyl aldehydes, and other organic compounds, including amidines, amines, alcohols, oximes, hydrazines, ylides, isonitriles, arylnitrones, nitrosobenzenes, isatins, 1,3-dicarbonyl compounds, *etc. via* transition metal-catalyzed, metal-free or visible light-promoted the transformations. In addition, several challenging mechanistic studies and the scope of some substrate ranges have been described.

## 2. Synthesis of N-heterocyclic compounds

### 2.1. Synthesis of triazoles

**Scheme 2:**
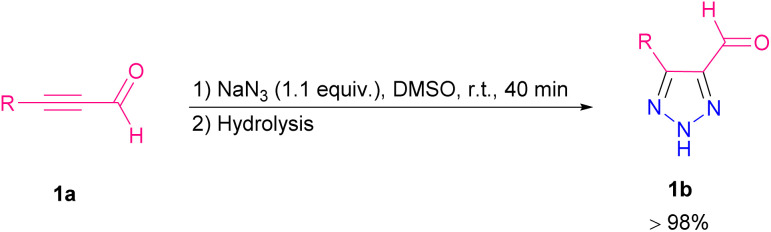
Synthesis of 5-substituted-4-carbaldehyde-1,2,3-triazoles.

Triazoles are interesting compounds for many years in terms of biological properties.^4^ In 2001, Journet and co-workers synthesized 5-substituted-4-carbaldehyde-1,2,3-triazoles 1b through the reaction of α,β-acetylenic aldehydes 1a with sodium azide (NaN_3_) (Scheme 2).^5^ It is essential to keep the reaction alkaline to prevent the generation of hazardous HN_3_.

### 2.2. Synthesis of imidazoles

**Scheme 3:**
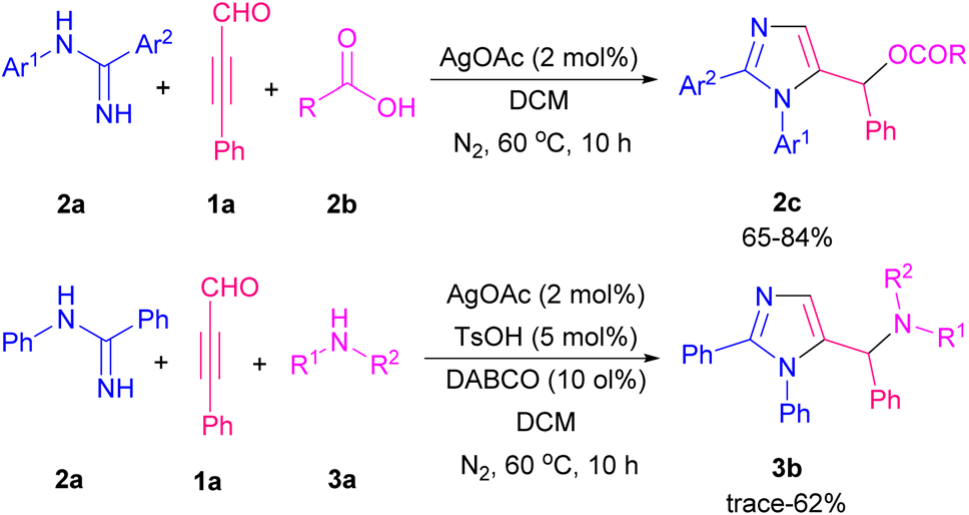
Synthesis of imidazoles using acids and amines as a nucleophile.

**Scheme 4:**
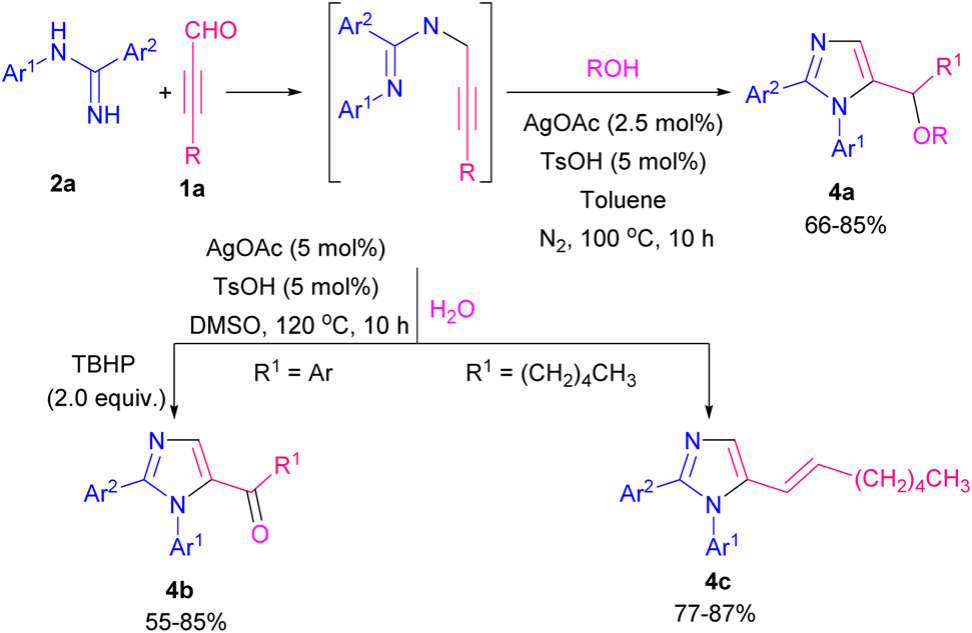
Reaction of amidines, propargyl aldehydes, and phenols, alcohols, or water.

Imidazole and its derivatives are highly polar five-membered aromatic heterocycles present in various natural products, applied pharmaceuticals, and biologically active molecules.^6^ In 2017, a three-component reaction of amidines 2a, aryl alkynyl aldehydes 1a, and carboxylic acids 2b or amines 3a was accomplished by Li and Cao *et al.* (Scheme 3).^7^ For the synthesis of imidazole products 2c, and 3b, various Ag salts such as AgOAc, AgOTf, Ag_2_O, AgSbF_6_, AgNO_3_, AgBF_4_, and AgCl, as the metal catalysts were examined, in which the best reaction yield was obtained in the presence of AgOAc. In this ionic pathway, carboxylic acids and amines acted as nucleophiles to regenerate Ag(i) from the Ag complex. After a while, the same group achieved imidazole-5-carbaldehydes in the presence of amidines and aryl alkynyl aldehydes using AgOTf as the optimized catalyst. The [3 + 2] domino reaction proceeded under O_2_ as a green oxidant.^8^ Cao and co-workers by controlling the substrates, could achieve diversely functionalized imidazoles from the reaction of amidines 2a, propargyl aldehydes 1a, and phenols, alcohols, or water (Scheme 4).^9^ When propargyl aldehydes 1a reacted with aliphatic and aromatic alcohols in the presence of a silver catalyst, imidazole products 4a were obtained. When alkynyl 1a (R = aryl) reacted with H_2_O in the presence of TBHP, arylimidazoles 4b were detected, while the reaction of alkynyl 1a (R = alkyl) with H_2_O without using any peroxide resulted in vinylimidazoles 4c.

**Scheme 5:**
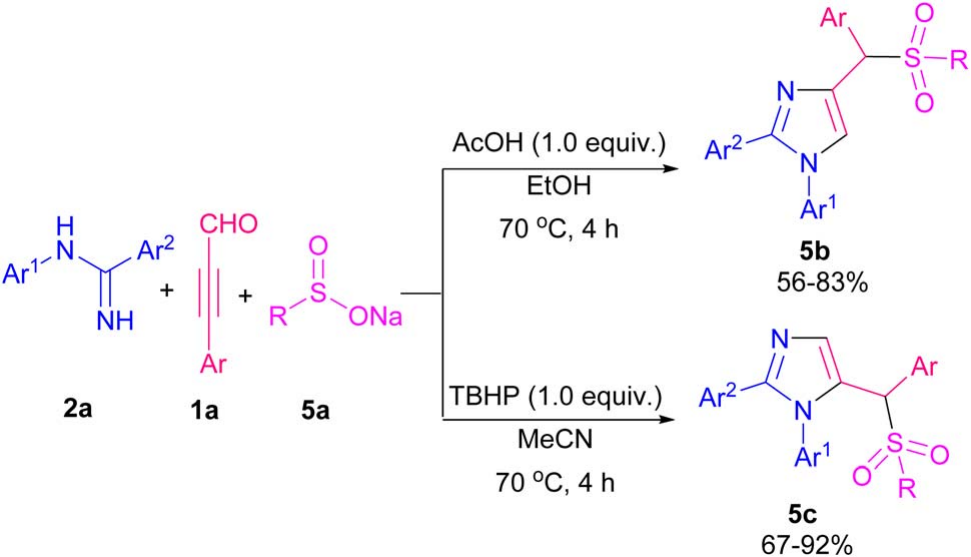
Synthesis of 4- or 5-sulfonated imidazoles using AcOH or TBHP as the additive.

**Scheme 6:**
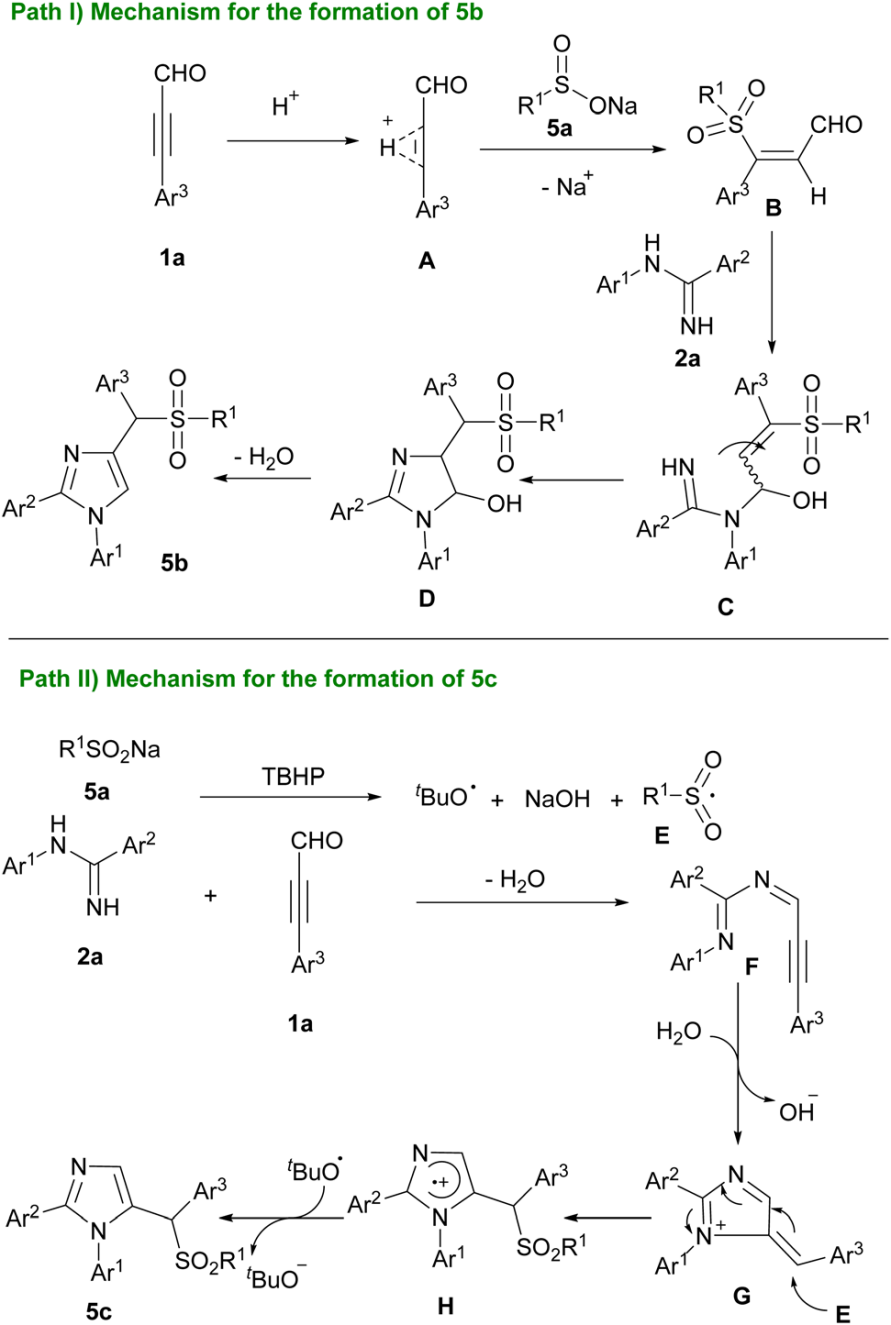
Two mechanistic pathways for the synthesis of 4- or 5-sulfonated imidazoles using AcOH or TBHP as the additive.

**Scheme 7:**
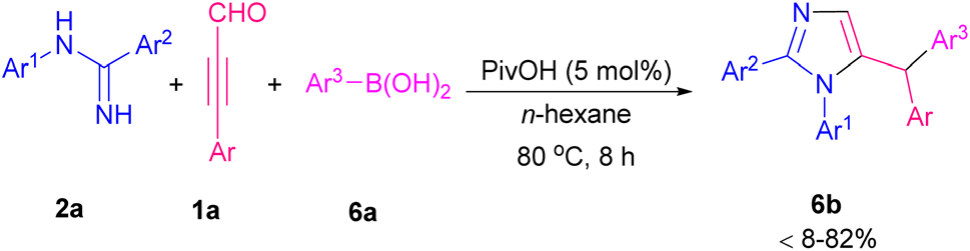
Synthesis of imidazole-containing triarylmethanes promoted by pivalic acid.

Cao's research group designed two transition metal-free reactions for the construction of imidazole derivatives. In one work, they used amidines 2a, aryl alkynyl aldehydes 1a, and sodium sulfonates 5a to provide 4- or 5-sulfonylated imidazoles by additive control (Scheme 5).^10^ Two possible pathways suggested for the reaction in the presence of AcOH or TBHP as the additives (Scheme 6). In path I, cation A generated by acid, which was subjected to nucleophilic addition of 5a to afford intermediate B. Subsequently, nucleophilic attack of 2a to B gave C that underwent intramolecular cyclization formed D. Product 5b produced by dehydration of D. In path II, oxidation of 5a by TBHP furnished sulfonyl radical E. Intermolecular dehydration of 1a and 2a resulted in imine intermediate F. Intramolecular annulation and protonation of F created G. Adding sulfonyl radical E to G and aromatization led to the production of product 5c. The same group, in a similar transformation, used boronic acids 6a with amidines 2a, and aryl alkynyl aldehydes 1a where pivalic acid acted as a catalyst for the synthesis of imidazole-containing triarylmethanes 6b (Scheme 7).^11^ In continuation of previous works, Cao *et al.* reported the preparation of 4- and 5-hydroxyalkyl-substituted imidazoles using amidines, aryl alkynyl aldehydes, and H_2_O in a controllable regioselective route.^12^ When they employed TsOH/NaSO_2_CF_3_ catalytic system, 4-hydroxyalkyl-substituted imidazoles were obtained while they achieved 5-hydroxyalkyl-substituted imidazoles in the presence of CuI.

### 2.3. Synthesis of imidazo[1,2-*a*]pyridines

**Scheme 8:**
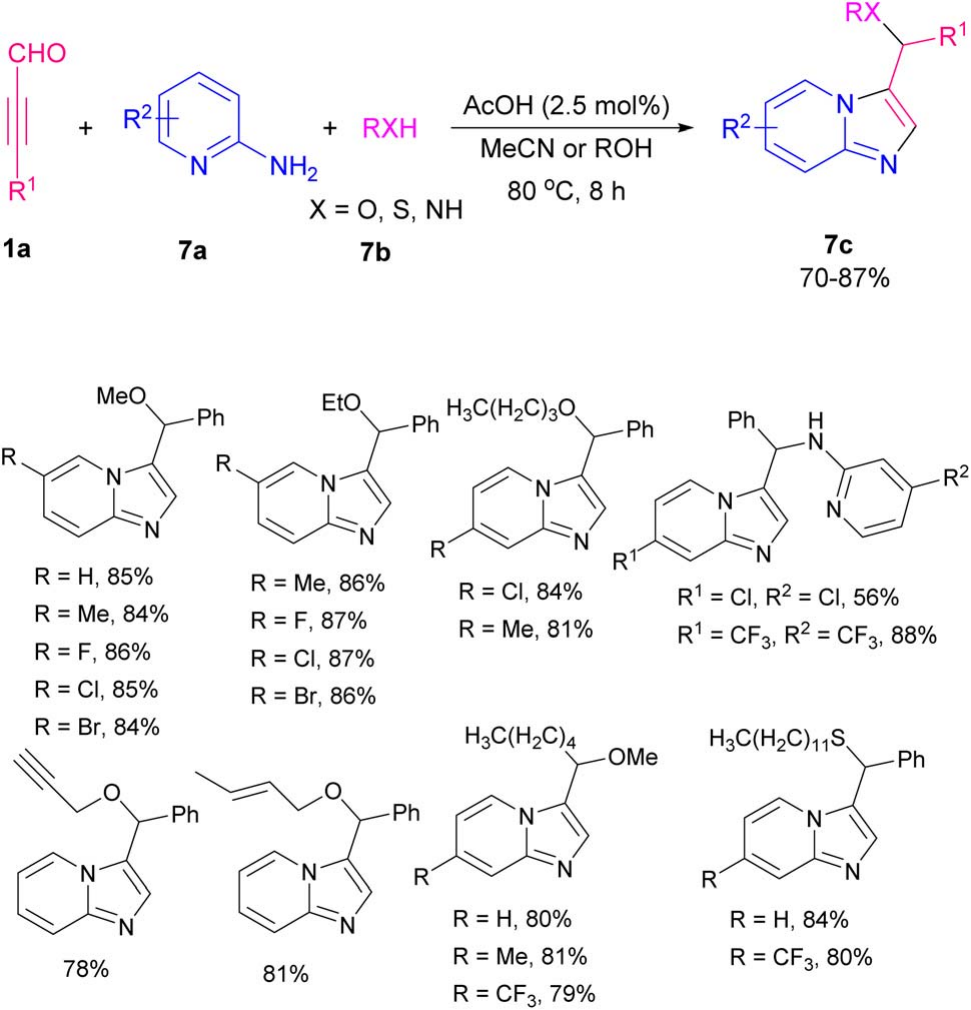
Metal-free synthesis of imidazo[1,2-*a*]pyridine derivatives.

**Scheme 9:**
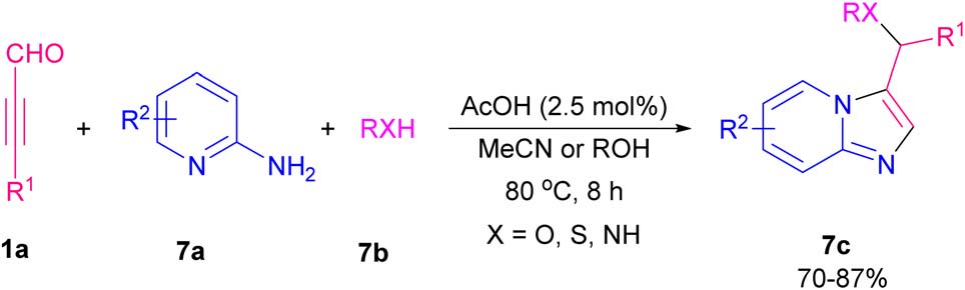
F_3_CCO_2_H-promoted formation of sulfoether-decorated imidazo[1,2-*a*]pyridines.

Imidazo[1,2-*a*]pyridine scaffolds are recognized as an important structural motif in the field of organochemistry due to divergent bioactivity features.^13^ In 2014, an efficient metal-free approach was developed by Cao *et al.* for the construction of imidazo[1,2-*a*]pyridine derivatives 7c, 2-amino pyridine 7a, aryl alkynyl aldehydes 1a, and alcohols, thiols or amines 7b as starting materials contributed in the one-pot C–N, C–O, or C–S bond formation process. The reaction worked well for both alcohols, thiols, and amines (Scheme 8).^14^ Subsequently, the formation of functionalized imidazo[1,2-*a*]pyridine aldehydes/ketones and 3-vinyl imidazo[1,2-*a*]pyridines *via* the reaction of aryl alkynyl aldehydes and 2-amino pyridines in the presence of Pd(ii) and Cu(i) catalysts was developed by the previous group.^15^ After a while, they could synthesize sulfoether-decorated imidazo[1,2-*a*]pyridines 7c by utilizing aryl alkynyl aldehydes 1a, 2-amino pyridines 7a, and aliphatic thiols 7b. F_3_CCO_2_H catalyzed C–N and C–S bond formation under microwave 500 W in 30 minutes (Scheme 9).^16^

**Scheme 10:**
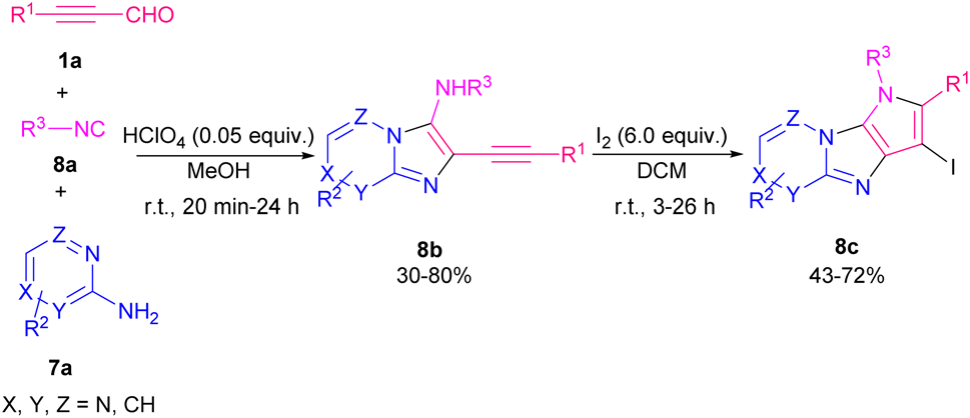
I_2_-promoted synthesis of 3-iodo-1*H*-pyrrolo[3′,2′:4,5]imidazo-[1,2-*a*]pyridines.

**Scheme 11:**
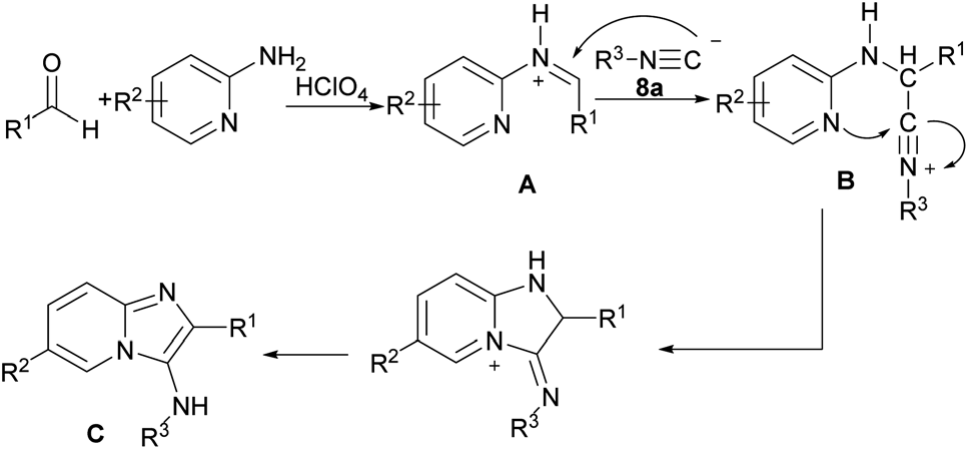
Groebke–Blackburn–Bienaymé MCR reaction toward 3-aminoimidazo[1,2-*a*]pyridine.

**Scheme 12:**
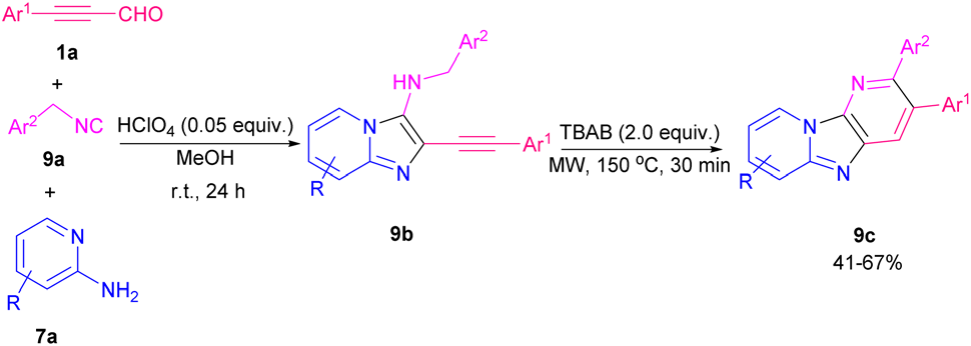
TBAB-mediated synthesis of imidazo-dipyridines.

I_2_-promoted electrophilic cyclization strategy for the preparation of 3-iodo-1*H*-pyrrolo[3′,2′:4,5]imidazo-[1,2-*a*]pyridines 8b and [1,2-*b*]pyridazines 8c from aryl alkynyl aldehydes 1a, 2-amino pyridines 7a and isonitriles 8a was developed (Scheme 10).^17^ Generally, in a Groebke–Blackburn–Bienaymé MCR reaction, the intermediate imine A underwent the nucleophilic addition of an isonitrile to afford the nitrilium ion B, followed by intramolecular cyclization and dehydration to achieve 3-aminoimidazo[1,2-*a*]pyridine C. In the next step, by the addition of molecular iodine, subsequent cyclization took place (Scheme 11). The reaction of 2-alkynyl aldehydes with 2-aminopyridines in the presence of FeCl_3_ (5 mol%) as a Lewis acid catalyst in toluene as a solvent at 60 °C led to a series of 3-arylimidazo[1,2-*a*]pyridines in moderate to good yields (41–85%).^18^ In 2019, a novel approach to diverse imidazo-dipyridines in a similar manner was introduced in two steps. In first step, the reaction between aryl alkynyl aldehydes 1a, 2-amino pyridines 7a, and isonitriles 9a resulted in product 9b, which by tetrabutylammonium bromide (TBAB), second cyclization occurred to achieve imidazo-dipyridine product 9c (Scheme 12).^19^ Anticancer activity of products against human prostate cancer also were determined by biological evaluation.

**Scheme 13:**
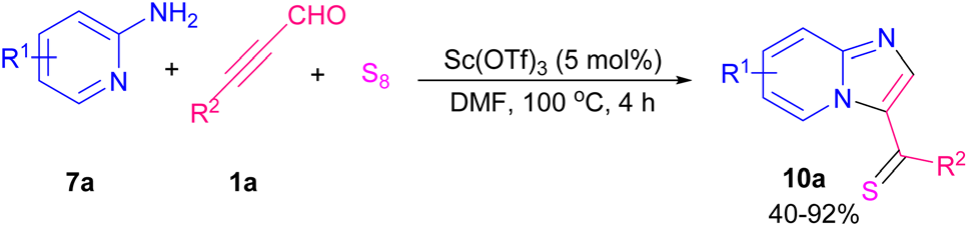
Lewis acid-catalyzed synthesis of imidazo[1,2-*a*]pyridine thiones.

**Scheme 14:**
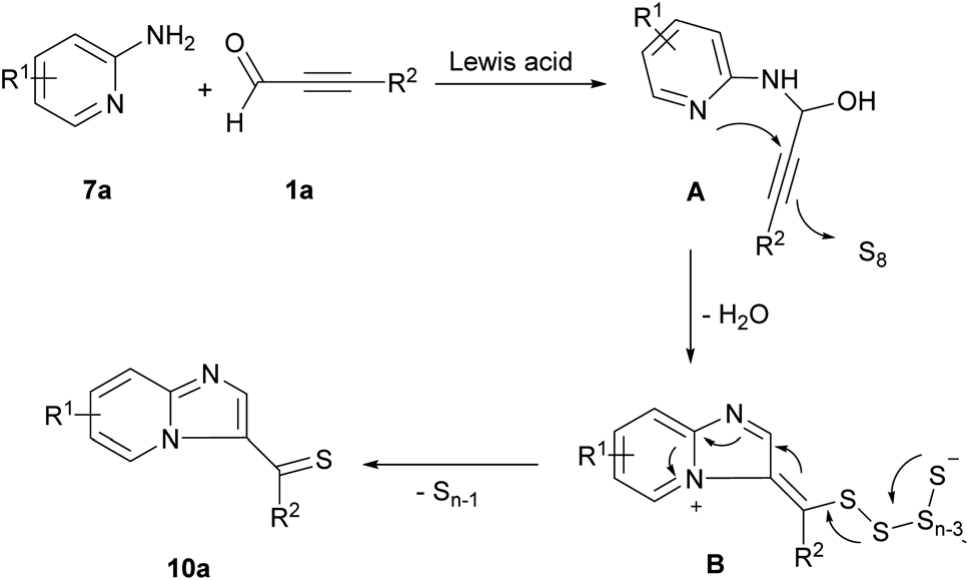
Proposed mechanism for synthesis of imidazo[1,2-*a*]pyridine thiones.

**Scheme 15:**
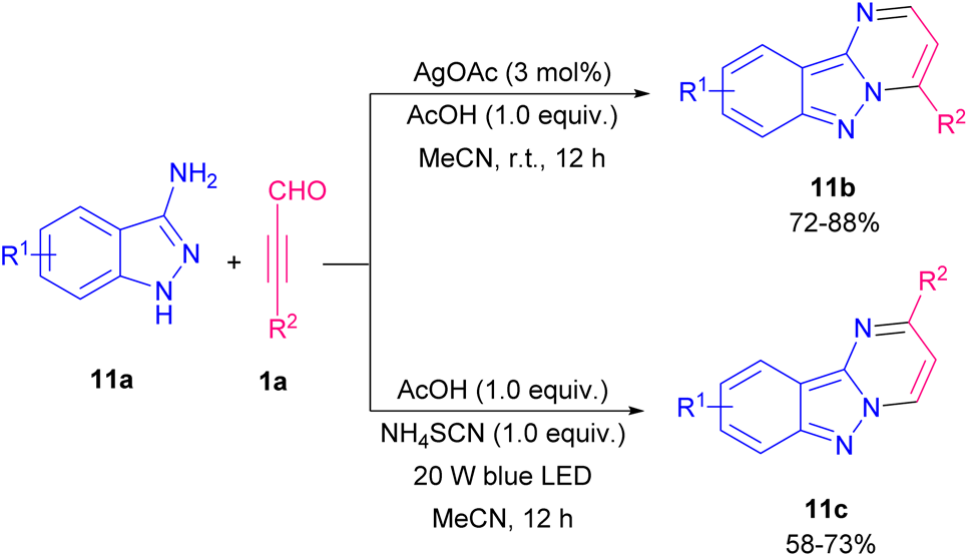
Controllable site-selective synthesis of 2- and 4-substituted pyrimido[1,2-*b*]indazoles.

Chen and co-workers investigated Lewis acid-catalyzed synthesis of imidazo[1,2-*a*]pyridine thiones in moderate to excellent yields through the reaction of 2-amino pyridines 7a, aryl alkynyl aldehydes 1a, and sulfur element (Scheme 13).^20^ The reaction began with Lewis acid-promoted the generation of intermediate A through nucleophilic addition of 7a to 1a. Nucleophilic attack of the nitrogen to the C≡C and activation of S8 by dehydration afforded intermediate B. Elimination process of B led to imidazo[1,2-*a*]pyridine thione 10a (Scheme 14). Cao *et al.* extended a strategy for the preparation of imidazo[1,2-*a*]pyridine by gold-catalyzed coupling reaction of propargyl aldehydes and 2-aminopyridines and a series of 3-acylimidazo[1,2-*a*]pyridines were obtained in reasonable yields.^21^ In 2021, the first example of providing 2- and 4-substituted pyrimido[1,2-*b*]indazole 11b, and 11c in a controllable site-selective manner from 3-aminoindazoles 11a and aryl alkynyl aldehydes 1a was introduced by He and Cao *et al.* (Scheme 15).^22^ In this strategy, when AgOAc and AcOH were used as a catalytic system, 2-substituted pyrimido[1,2-*b*]indazoles were obtained, whereas if metal-free conditions containing AcOH and NH_4_SCN under blue LED irradiation were examined, a series of 4-substituted pyrimido[1,2-*b*]indazole products were detected.

### 2.4. Synthesis of indolizines

**Scheme 16:**
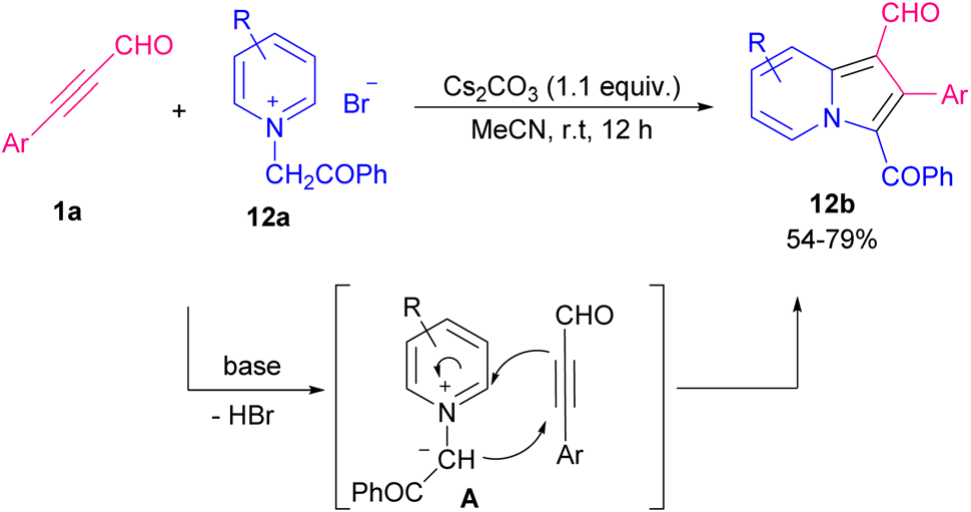
Base-mediated the reaction between aryl alkynyl aldehydes with pyridinium ylides.

**Scheme 17:**
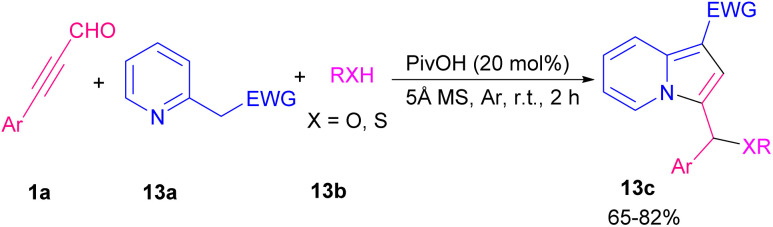
Acid-promoted the synthesis of indolizines.

**Scheme 18:**
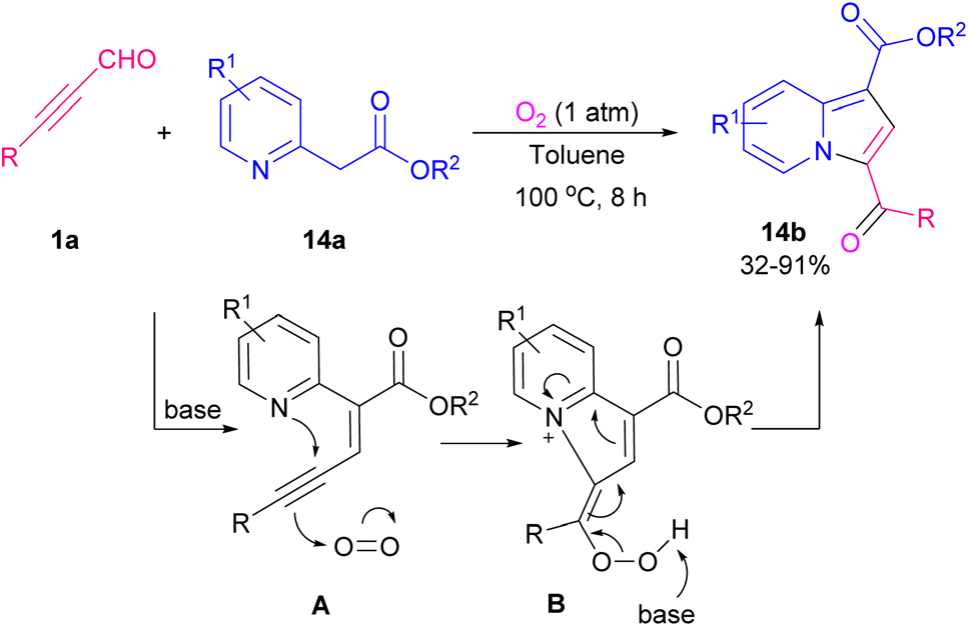
Metal-free formation of 3-acylated indolizines.

In 2016, Sun and Xing *et al.* described an easy practical strategy for providing indolizine-1-carbaldehydes *via* the reaction between aryl alkynyl aldehydes and pyridinium ylides (Scheme 16).^23^ Removal of HBr from pyridinium ylide 12a by a base resulted in nonstabilized pyridinium ylide A, which underwent 1,3-dipolar cycloaddition with alkynyl 1a to produce 12b. Synthesis of indolizines also can be assisted by an acid catalyst. 2-(pyridin-2-yl)acetates 13a, aryl alkynyl aldehydes 1a, and alcohols or thiols 13b participated in a multi-component reaction to construct a wide range of functionalized indolizines 13c in the presence of pivalic acid (Scheme 17).^24^ Molecular oxygen as a green oxidant employed in the cyclization of 2-pyridylacetates 14a and aryl/alkyl alkynyl aldehydes 1a to form 3-acylated indolizines 14b. Using radical scavenger for mechanism study revealed that the reaction proceeded under an ionic process involving Knoevenagel condensation of 1a with 14a, nucleophilic attack, the addition of O_2_, and elimination of OH group (Scheme 18).^25^

**Scheme 19:**
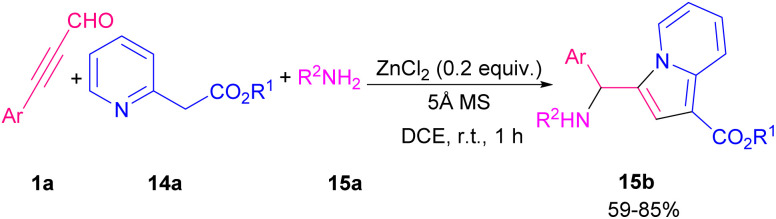
Metal-free formation of 3-acylated indolizines.

**Scheme 20:**
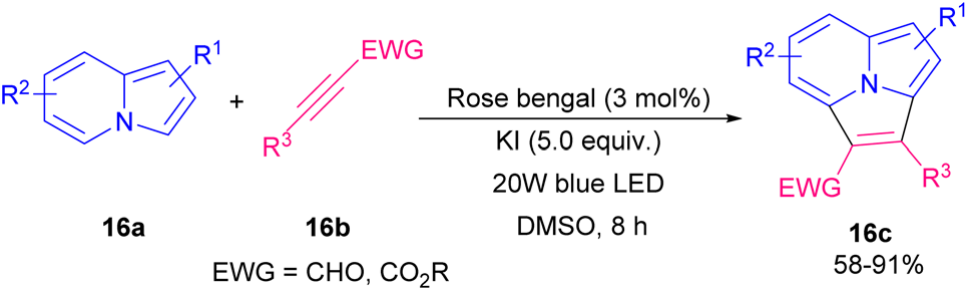
Zn-catalyzed synthesis of pyrrolo[2,1,5-*cd*]indolizine.

**Scheme 21:**
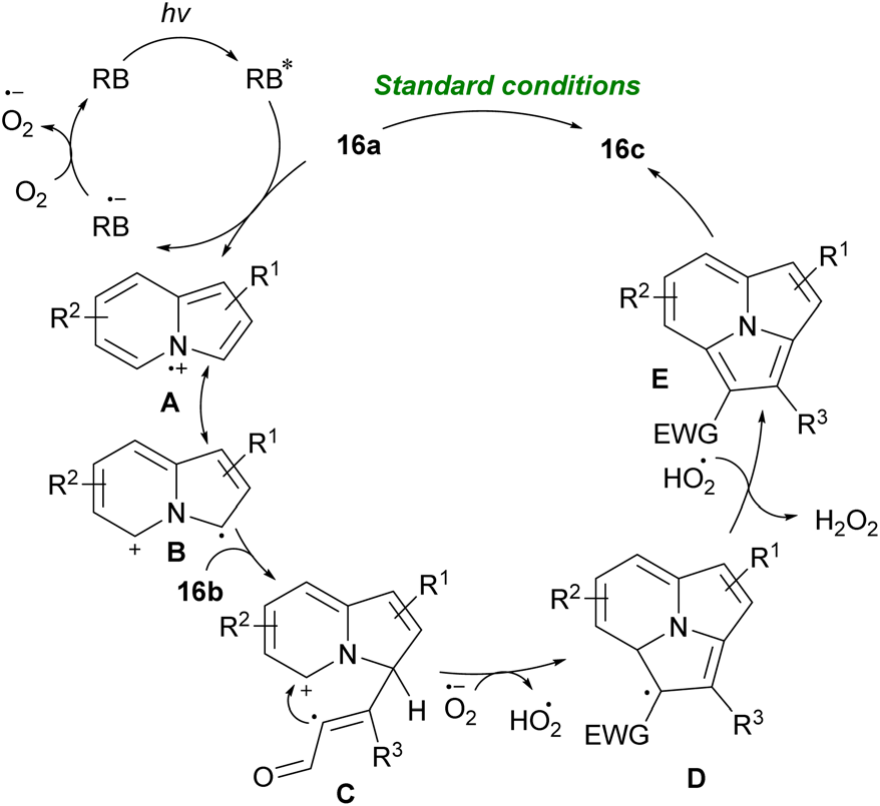
Visible-light-induced intermolecular [3 + 2] cycloaddition of indolizines.

In 2020, aryl alkynyl aldehydes were also employed by Zhu and Cao's research team to furnish aminoalkyl indolizine derivatives (Scheme 19).^26^ Thus, the reaction of 2-(pyridin-2-yl)acetates 14a, aryl alkynyl aldehydes 1a, and amines 15a in the presence of 0.2 equivalent of zinc(ii) chloride afforded the desired aminoalkyl indolizine products 15b in good yields. Their transformation had the advantages of an inexpensive catalyst, short reaction time, mild conditions, and easy product separation. Related work by the same group demonstrated that the use of rose bengal (RB) as a photocatalyst under visible-light irradiation could catalyze constructing pyrrolo[2,1,5-*cd*]indolizine rings 16c in good to high yields (Scheme 20).^27^ Intermolecular [3 + 2] annulation of indolizines with internal alkynes under 20 W LED irradiation as an energy source using various photocatalysts such as RB, eosin Y, eosin B, rhodamine 6G, and fluorescein was carried out. The best results were obtained in the presence of RB. Mechanistically, the reaction is proposed to proceed *via* the conversion of ground state RB to excited state RB* under visible-light irradiation, which reacts with 16a to form the radical intermediate A. Next, the intermolecular addition of intermediate B to alkyne 16b led to intermediate C that underwent sequential dehydrogenation oxidation *via* O_2_˙^−^ generated D and E. Finally, aromatization of E produced 16c (Scheme 21).

### 2.5. Synthesis of pyrroles

**Scheme 22:**
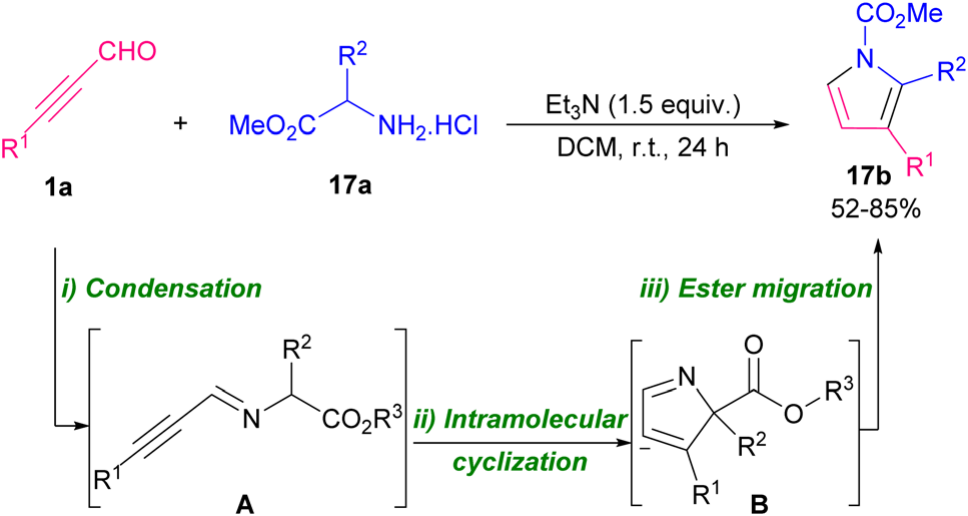
Metal-free-cascade reaction of α-amino esters and alkynals.

**Scheme 23:**
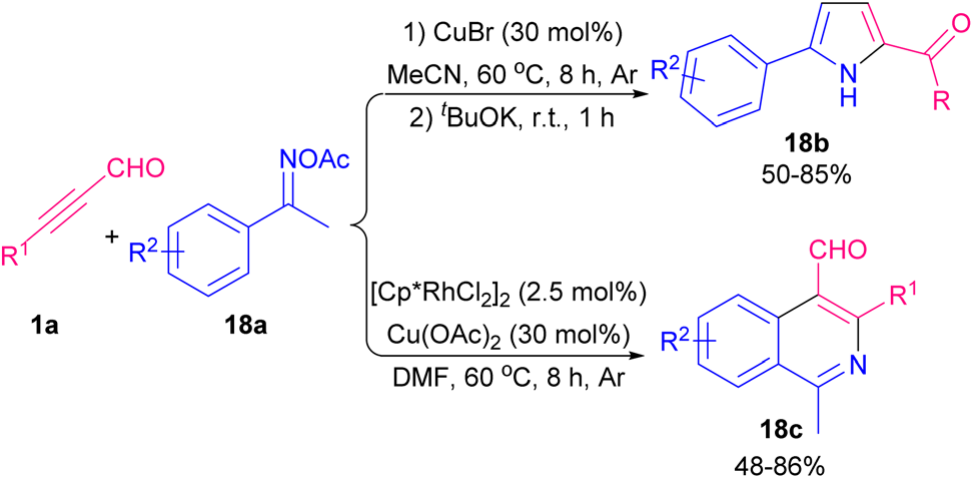
Transition metal-catalyzed synthesis of pyrrole and isoquinoline scaffolds.

**Scheme 24:**
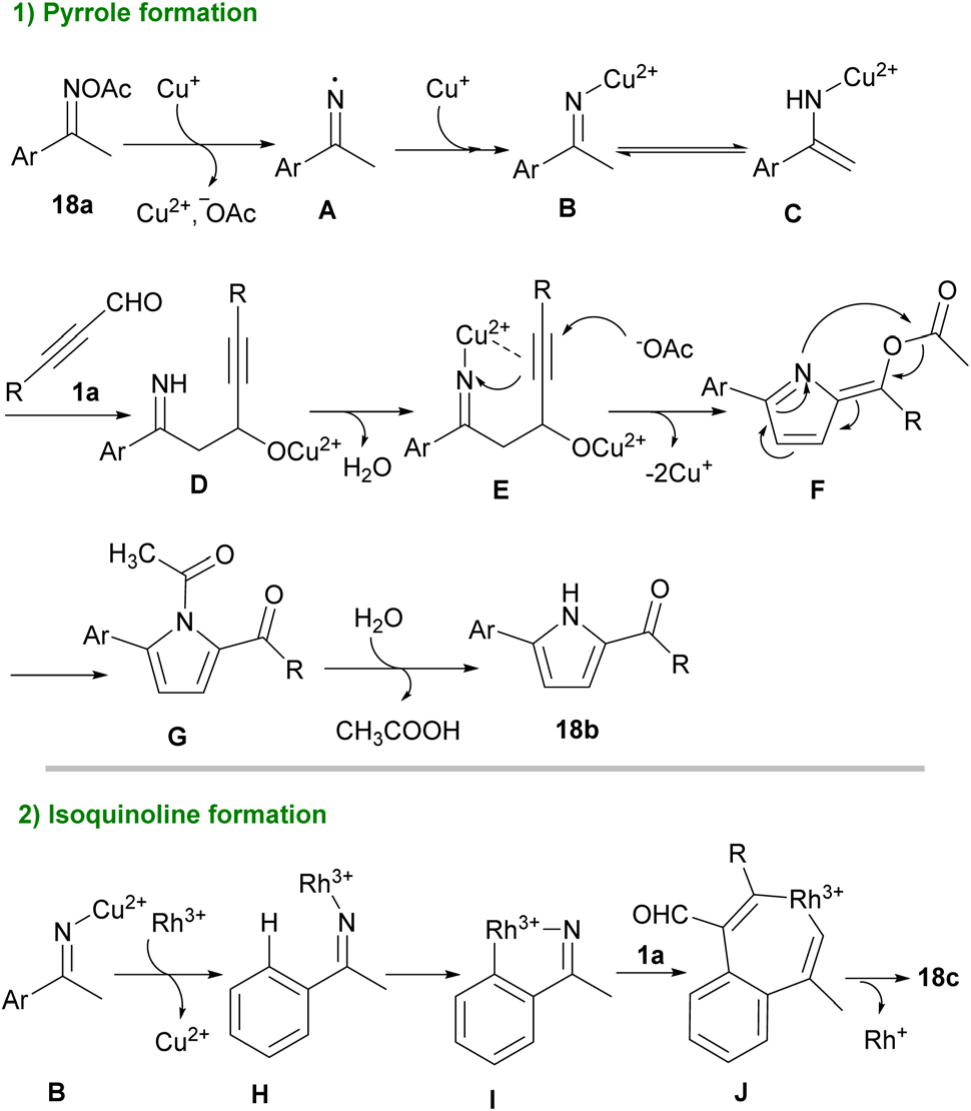
Proposed mechanisms for transition metal-catalyzed synthesis of pyrrole and isoquinoline scaffolds.

**Scheme 25:**
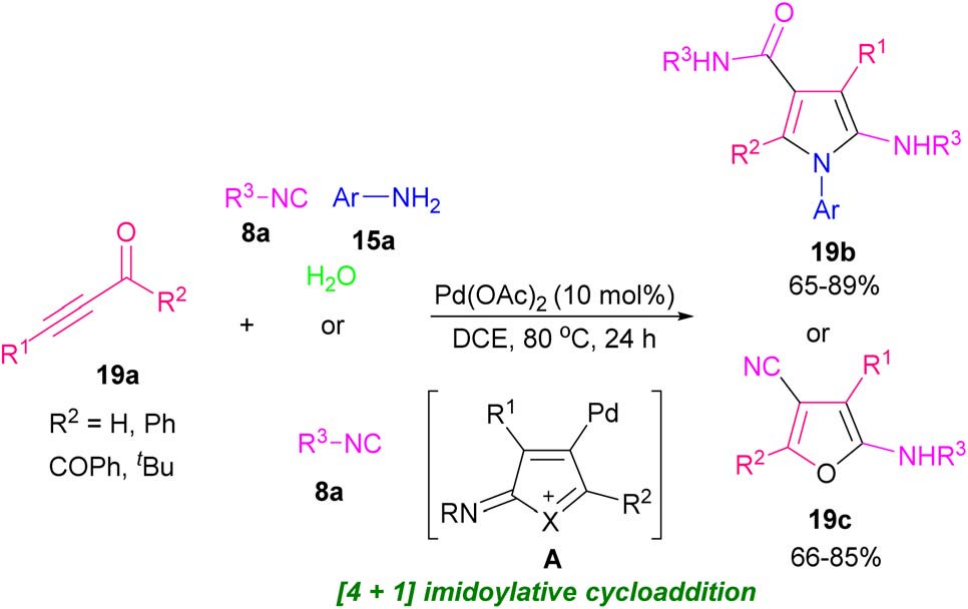
Pd-catalyzed [4 + 1] cyclization of prop-2-yn-1-ones and isocyanides.

Pyrroles are recognized as valuable simple five-membered heterocycles, due to their existence in numerous pharmaceutical agents,^28^ molecular optics,^29^ conducting polymers,^30^ electronics,^31^ gas sensors for organic molecules,^32^ and also scaffolds in diverse physiologically natural products.^33^ In 2020, Wang and co-workers demonstrated the use of triethyl amine in the reaction of α-amino acid esters hydrochloride 17a and alkynyl aldehydes 1a for the formation of 1,2,3-trisubstituted pyrroles 17b. The authors suggested that the reaction proceeded *via* the cascade condensation/intramolecular cyclization reaction followed by a C–N ester migration (Scheme 22).^34^ In related work, Chen *et al.* by using transition metal catalysts like copper and rhodium complex could synthesized pyrrole 18b and isoquinoline 18c scaffolds (Scheme 23).^35^ From [3 + 2] annulation of ketoxime acetates 18a and propargyl aldehydes 1a in the presence of Cu(i) salt and a base, pyrrole derivatives 18b were obtained. At the same time, Rh(iii) and Cu(ii) as co-catalysts triggered the [4 + 2] annulation of substrates to afford isoquinoline derivatives 18c. Two plausible mechanisms are suggested for synthesizing pyrroles and isoquinolines. Sequential single-electron reduction can form intermediate C that involves tautomerization from oxime acetate 18a. In the next step, nucleophilic addition of C to aldehyde 1a produced intermediate D, and H_2_O elimination of D created intermediate E. Consequently, pyrrole G was generated *via* a nucleophilic attack and cyclization perused by a rearrangement process. Eventually, product 18b formed *via* the *N*-acetylation step (Scheme 24, part 1). For achievement to isoquinolines, iminyl-Cu(ii) B with Rh(iii) gave rhodacyclic intermediate G through an iminyl rhodium intermediate I. Intercalation of aldehyde 1a afforded intermediate K, which was transformed into product 18c. Rh(iii) and Cu(i) are released through the redox reaction between Rh(i) and Cu(ii) (Scheme 24, part 2). Internal alkynes were also employed by Chen *et al.* to form 2-amino-4-amidylpyrroles 17e and 2-amino-4-cyanofurans 17c through an [1 + 4] imidolative cycloaddition process (Scheme 25).^36^ Thus, the reaction of aryl/alkyl/heteroaryl prop-2-yn-1-ones 19a with isocyanides 8a, aryl amines 15a, and H_2_O in the presence of catalytic amount of palladium acetate furnished pyrrole rings 19b. Interestingly, under these conditions, prop-2-yn-1-ones 19a were reacted with isocyanides 8a to afford the desired furan rings 19c.

**Scheme 26:**
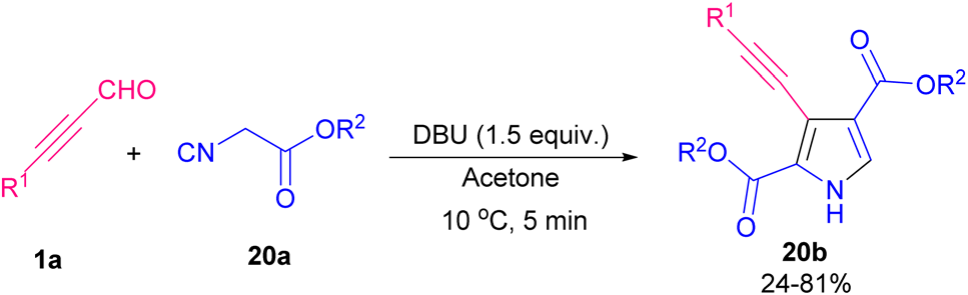
Constructing 3-alkynylpyrrole-2,4-dicarboxylates from methylene isocyanides and alkynyl aldehydes.

**Scheme 27:**
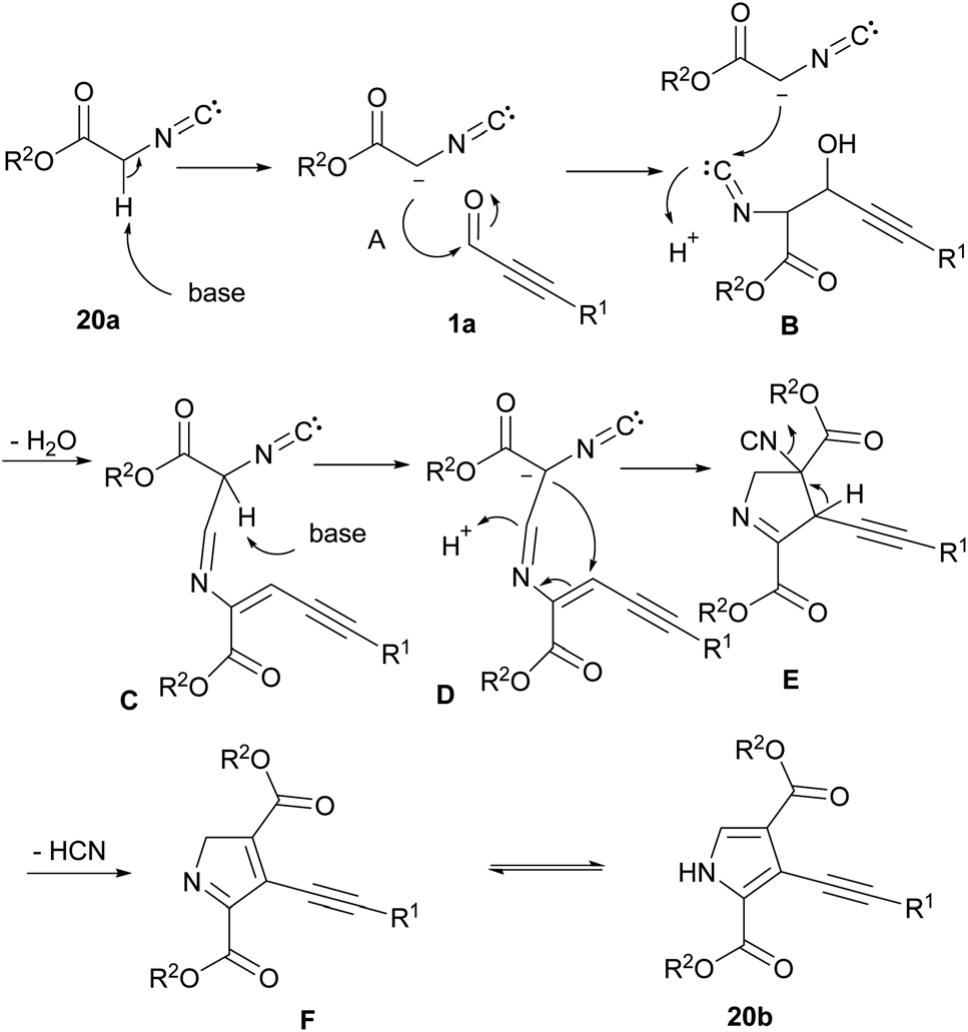
Plausible mechanism for constructing 3-alkynylpyrrole-2,4-dicarboxylates.

A convenient transition metal-free approach for constructing 3-alkynylpyrrole-2, 4-dicarboxylates 20b from alkynyl aldehydes 1a, and methylene isocyanides 20a was introduced by Shao's laboratory in 2021 (Scheme 26).^37^ A tentative mechanism was proposed for the system. As shown in Scheme 27, firstly, isocyanide 20a was deprotonated by DBU to form A. Nucleophilic attack A to 1a generated the intermediate B that underwent further attack by the second molecule 20a to afford C. Next, deprotonation of C followed by intra-molecular cyclization produced E. Removal of HCN created intermediate F, which upon interconversion furnished the target product 20b. Meantime, another procedure for the synthesis of acylpyrrole derivatives through the cyclization of ketoxime acetates and alkynyl aldehydes was performed by Huang *et al.*^38^ The reaction moved upon using 15 mol% of CuBr as the inexpensive metal catalyst to promote synthesizing 2,5-disubstituted and 1,2,5-trisubstituted pyrroles. Both electron-rich and electron-deficient aromatic rings were tolerated under these conditions and gave desired products in moderate yields.

### 2.6. Synthesis of pyrazoles

**Scheme 28:**
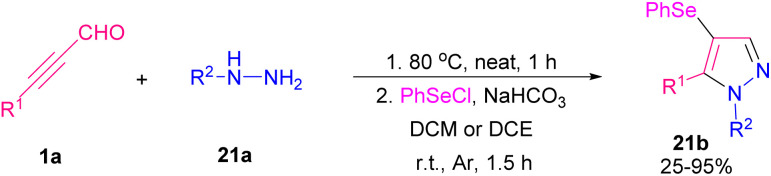
Synthesis of 4-(phenylselanyl)-substituted pyrazoles.

**Scheme 29:**
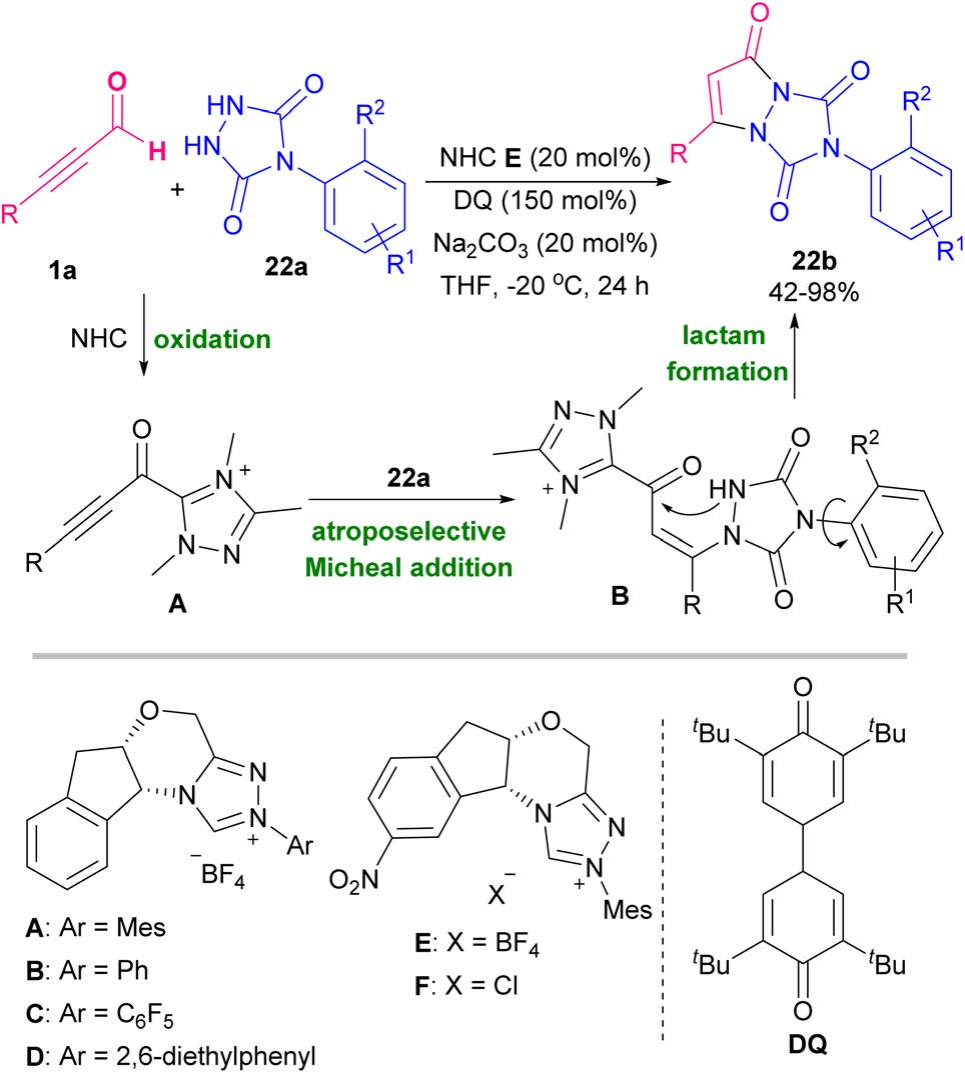
NHC-catalyzed annulation of alkynyl aldehydes and urazoles.

**Scheme 30:**
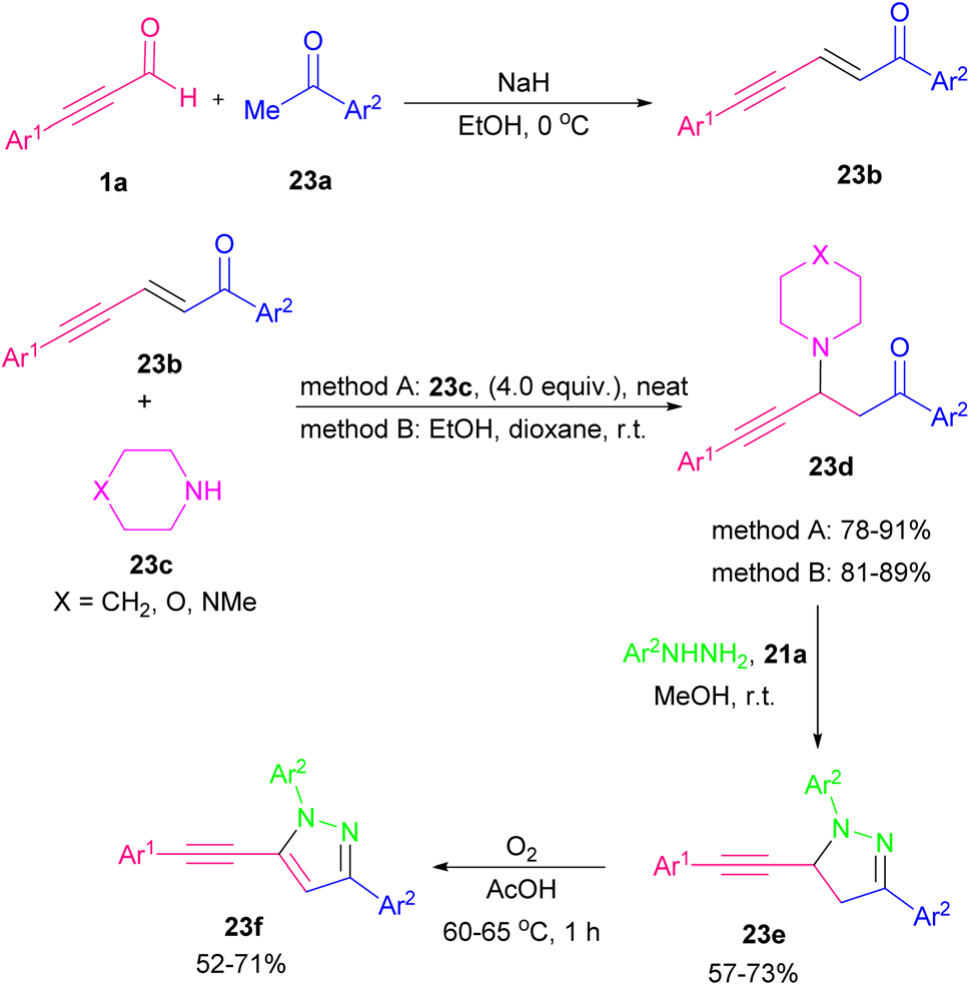
Synthesis of acetylenic 2-pyrazolines and pyrazoles.

Pyrazoles are well-known five-membered nitrogen-containing heterocycles that contain characteristic structural motifs exist in numerous natural products and pharmaceuticals.^39^ A general synthetic methodology to make 4-(phenylselanyl)-substituted pyrazoles 21b was described. The reaction started with the reaction of alkynyl aldehydes 1a with hydrazines 21a to generate *in situ* α,β-alkynyl hydrazones followed by the cyclization reaction with phenylselenyl chloride to produce 4-(phenylselanyl)pyrazoles 21b (Scheme 28).^40^ An annulation between alkynyl aldehydes 1a and urazoles 22a catalyzed by a chiral N-heterocyclic carbene (NHC) organocatalyst *via* atroposelective [3 + 2] Micheal addition and lactam ring formation was described by Chi research group in 2021. A broad range of axially chiral/heteroatom-rich pyrazolo[1,2-*a*]triazoles 22b with good enantioselectivities were achieved by this method. The reaction in the presence of chiral NHCs A, B, D, and E showed high chemical yields (Scheme 29).^41^ In 2022, Golovanov *et al.* investigated the formation of acetylenic 2-pyrazolines 23e and pyrazoles 23f from propargyl aldehyde 1a as starting material (Scheme 30).^42^ Propargyl aldehyde 1a in the reaction with arylmethyl ketone 23a led to 2,4,1-enynone 23b, which in the next step reacted with morpholine to generate propargylamine 23d. Finally, cyclocondensation of 23d in the presence of arylhydrazine 21a delivered pyrazoline 23e, which underwent further oxidative dehydrogenation to give pyrazole product 23f. The author found that the presence of cyclic amine was necessary for the cyclization reaction between 23d with arylhydrazine 21a. The N-heterocycle products demonstrated good fluorescent abilities.

### 2.7. Synthesis of pyridines

**Scheme 31:**
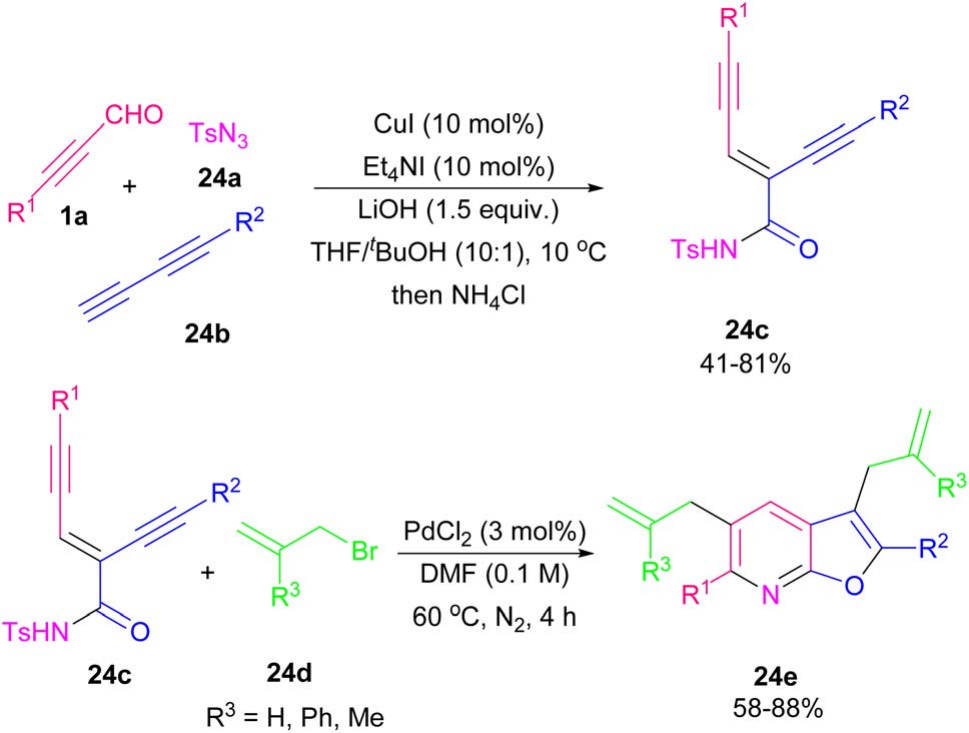
Pd-catalyzed cyclization of enediyne-imides toward furo[2,3-*b*]pyridines.

**Scheme 32:**
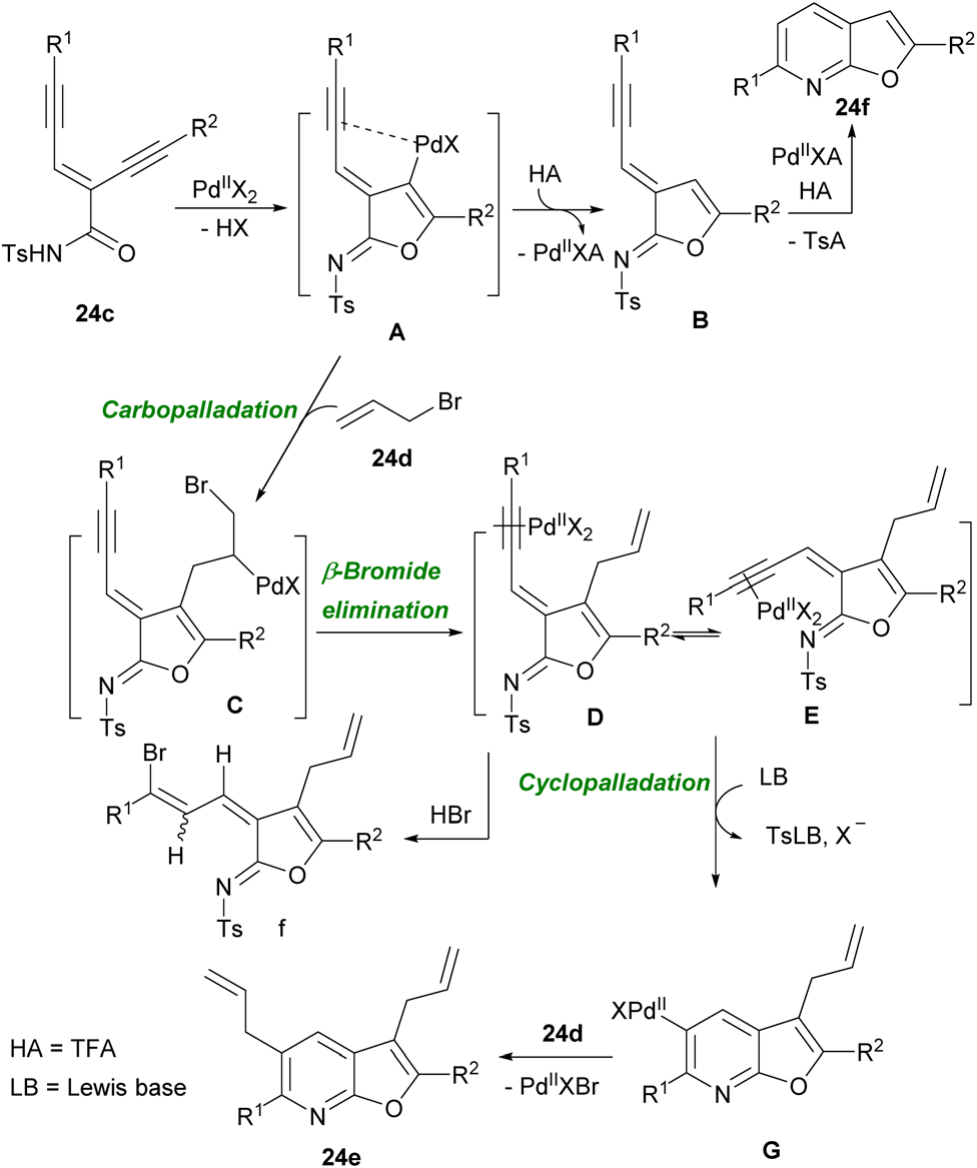
Proposed mechanism for Pd-catalyzed cyclization of enediyne-imides toward furo[2,3-*b*]pyridines.

**Scheme 33:**
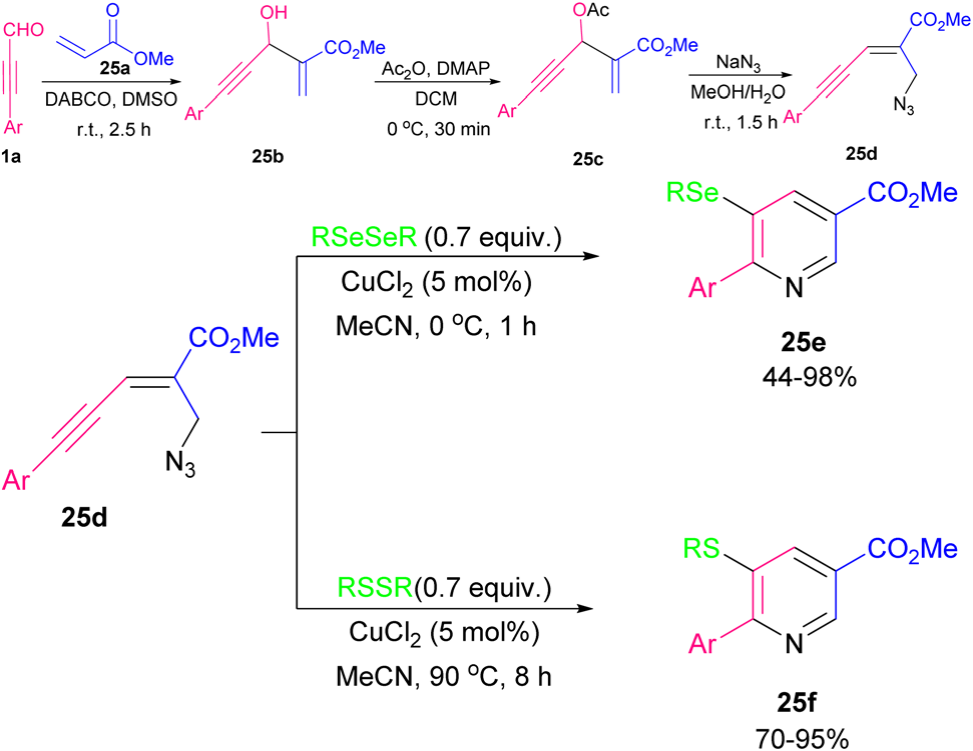
Cu-promoted synthesis of 5-selenyl/sulfenyl nicotinates.

In 2014, Ma's research group successfully synthesized 3,5-diallylfuro[2,3-*b*]pyridine and 2,6-disubstituted furopyridines 24e employing *N*-tosyl carboxamide moiety 24c as the *N*,*O*-bisnucleophile in the presence of palladium metal catalyst (Scheme 31).^43^ The authors proposed a mechanism for this cyclization system involving Pd-promoted *trans*-oxypalladation *via* 5-*endo*-dig to create the vinyl-palladium species A. Under acidic conditions, protonolysis of A formed imidate B, which underwent cycloisomerization to form product 24f with the removal of the tosyl groups. On the other hand, A coupled with 24d led to intermediate D through olefin insertion and β-bromide elimination. Next, olefin *E*/*Z* isomerization, and *N*-nucleophilic cyclopalladation of intermediate D, gave another palladium species G. Finally, G coupled with 24d to produce 24e (Scheme 32). Reddy and co-workers utilized enynyl azide 22e to construct substituted pyridines and 5-aminonicotinate derivatives in the presence of Ag(i) and Cu(i) catalysts, respectively.^44^ For the preparation of this substrated they performed a Morita–Baylis–Hillman (MBH) reaction using alkynyl aldehyde 1a and methyl acrylate 25a to obtain 25b. Then, 25b underwent *O*-acylation followed by allylic substitution with NaN_3_ to form enynyl azide 25d. In the next step, enynyl azide 25d was subjected to an intramolecular chalcogenoamination with diselenides and disulfides using Cu(ii) catalyst to produce 5-selenyl/sulfenyl nicotinates 25e, and 25f in moderate to excellent chemical yields (Scheme 33).^45^ This is the first example of the one-pot C–Se, C–S and C–N bond formation approach toward biologically important substituted 5-chalcogenyl nicotinates.

**Scheme 34:**
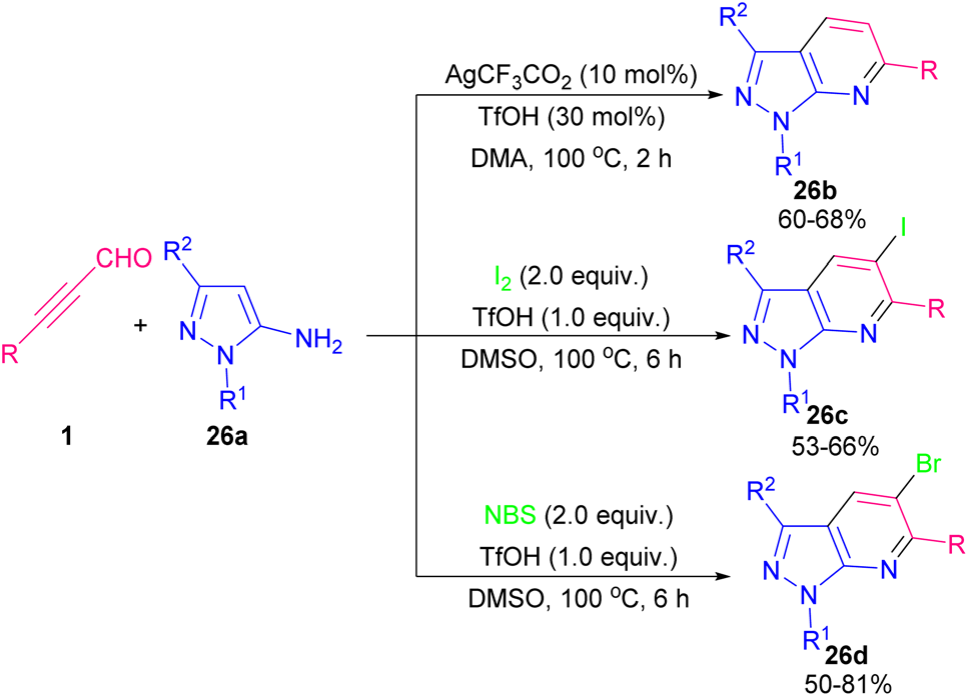
Synthesis of pyrazolo[3,4-*b*]pyridine frameworks from 5-aminopyrazoles and propargyl aldehydes.

**Scheme 35:**
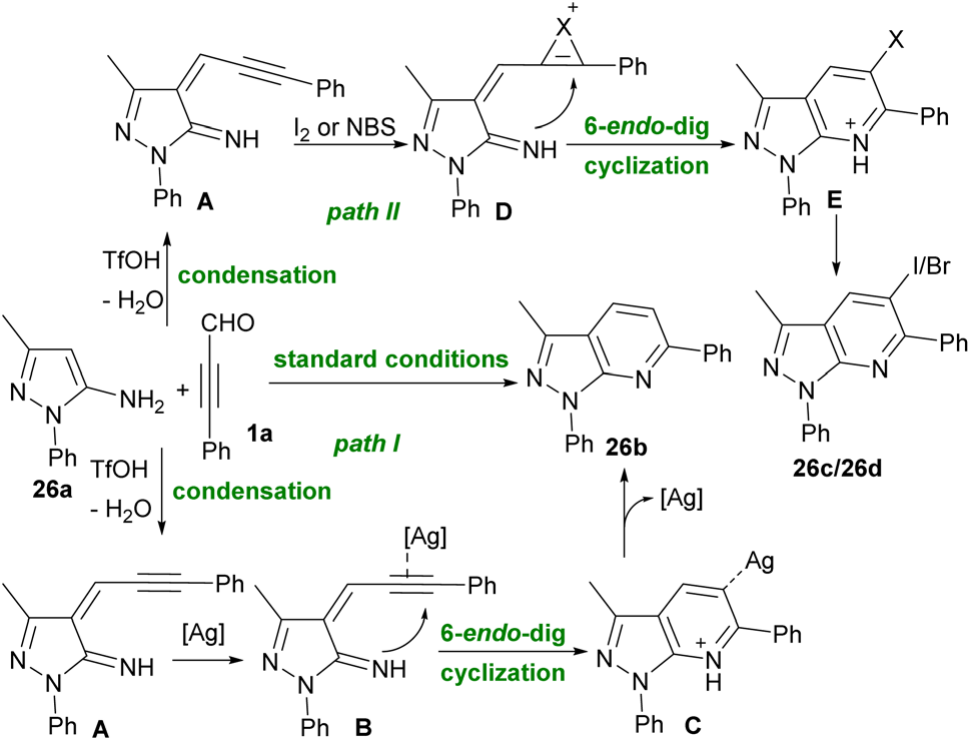
Proposed mechanism for synthesis of pyrazolo[3,4-*b*]pyridine frameworks from 5-aminopyrazoles and propargyl aldehydes.

The synthesis of pyridine derivatives from alkynyl aldehydes 1a and 5-aminopyrazoles 26a was reported by Zhu *et al.* in 2022 (Scheme 34).^46^ In this method, Ag, I_2_ and NBS were used to activate the C≡C bond of alkynyl aldehydes 1a. A wide variety of halogen and non-halogen-functionalized pyrazolo[3,4-*b*]pyridine compounds 26b, 26c, and 26d were achieved in moderate to good chemical yields. Two possible pathways are depicted in Scheme 35. In path I, the condensation of amine 2 with alkynyl aldehyde 1a generated intermediate A. The coordination of Ag salt into alkyne 1a formed intermediate B, which underwent 6-*endo*-dig cyclization to produce C. Finally, the demetallation of B delivered product 26b. In path II, the addition of I_2_ or NBS into a C≡C bond of alkyne A led to intermediate D, which underwent 6-*endo*-dig cyclization to create E. Elimination of a proton from E resulted in 26c or 26d.

**Scheme 36:**
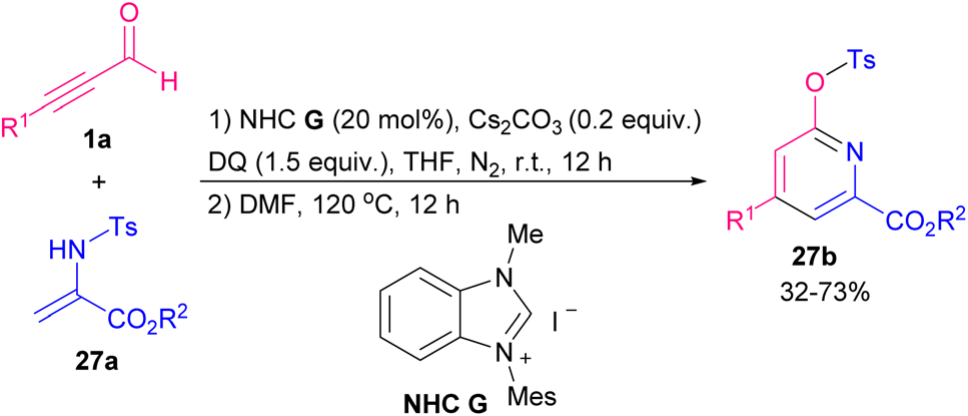
NHC-catalyzed construction of functional pyridines.

**Scheme 37:**
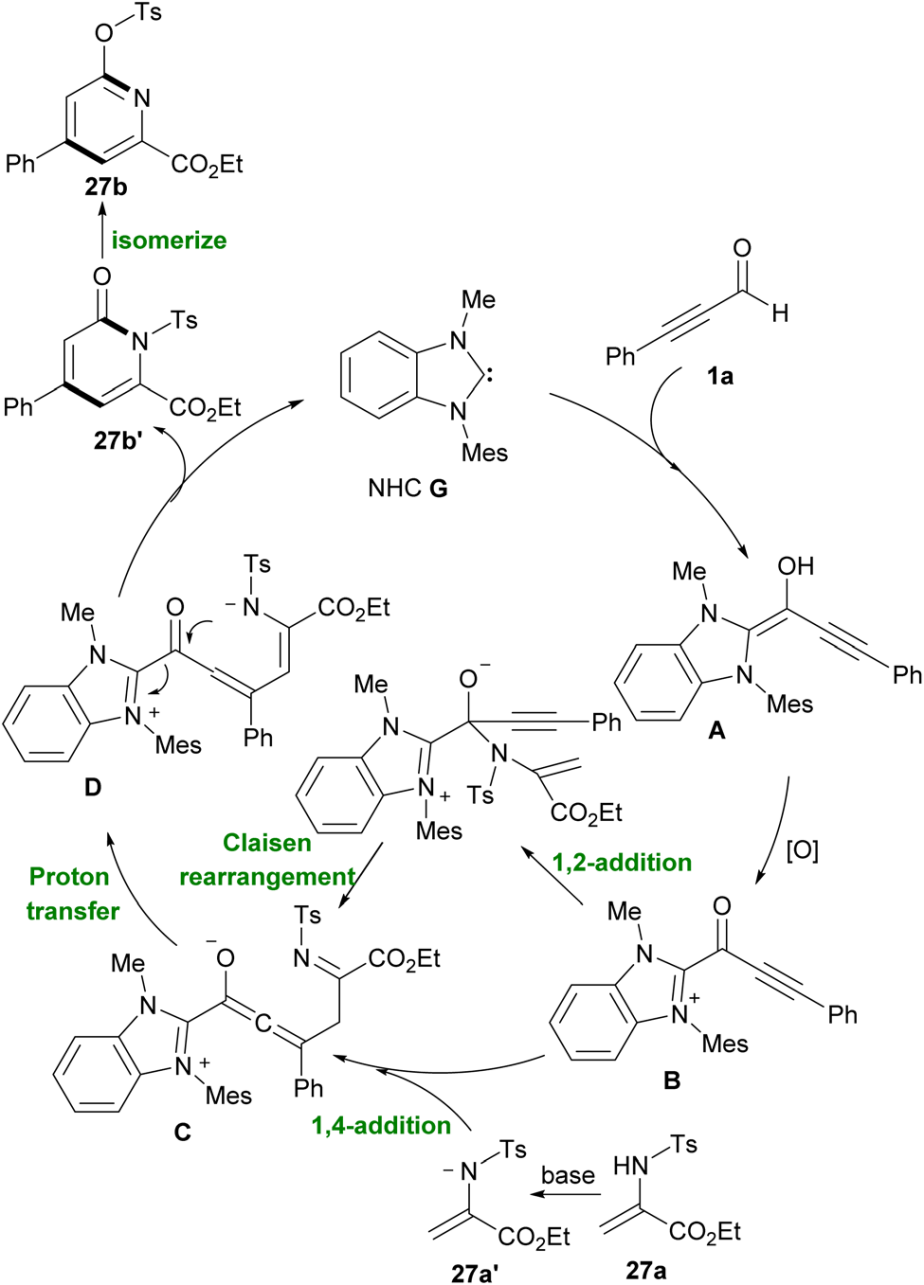
Plausible mechanism for NHC-catalyzed construction of functional pyridines.

In 2019 Duan and Qi *et al.* proposed a way to synthesize functional pyridines by which the reaction of NHC-bounded alkynyl acyl azolium with N-Ts-protected 2-aminoacrylate 27a led to final product 27b (Scheme 36).^47^ In these products, ester functional groups were directly attached to the pyridines. The synthesis of chiral pyridine ligands was another advantage of their work. Plausible mechanism of this report is depicted in Scheme 37. The addition of the NHC G to alkynyl aldehyde 1a produced the alkynyl intermediate A. Oxidative reaction of A gave NHC-bounded alkynyl acyl azolium B. Then nucleophilic 1,4-addition or 1,2-addition/Claisen rearrangement of deprotonated N-Ts 2-aminoacrylate 27a’ with B gave intermediate C, which in continue during a proton transfer process, produced intermediate D. Eventually, through an *N*-acylation reaction NHC catalyst released and the lactam product 27b formed.

### 2.8. Synthesis of pyrimidines

**Scheme 38:**
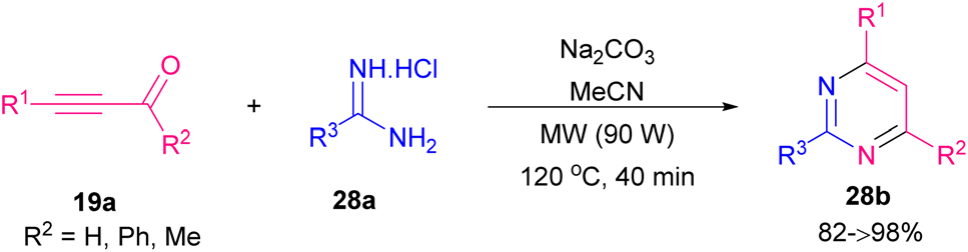
Cyclization reaction between alkynones and guanidine.

**Scheme 39:**
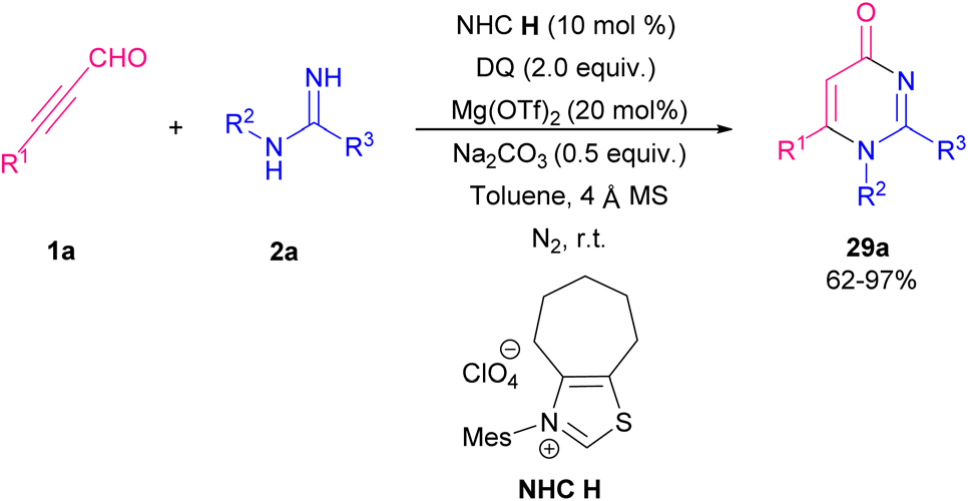
NHC-catalyzed reaction of alkynyl aldehydes and amidines.

**Scheme 40:**
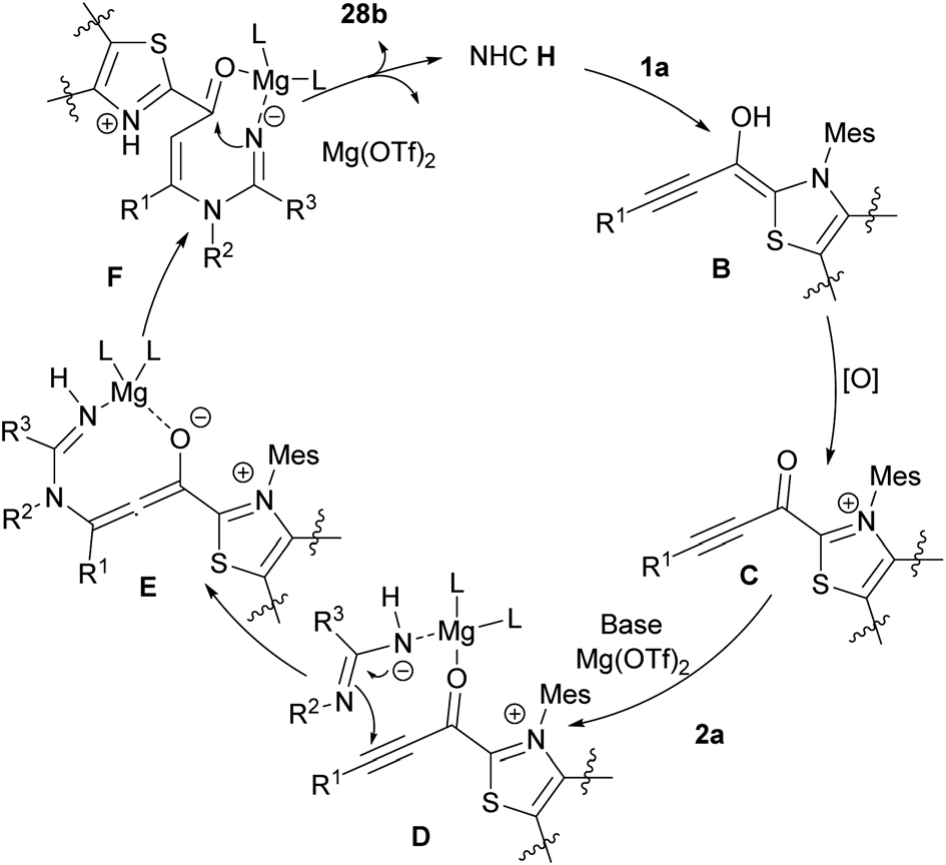
Proposed mechanism for NHC-catalyzed reaction of alkynyl aldehydes and amidines.

**Scheme 41:**
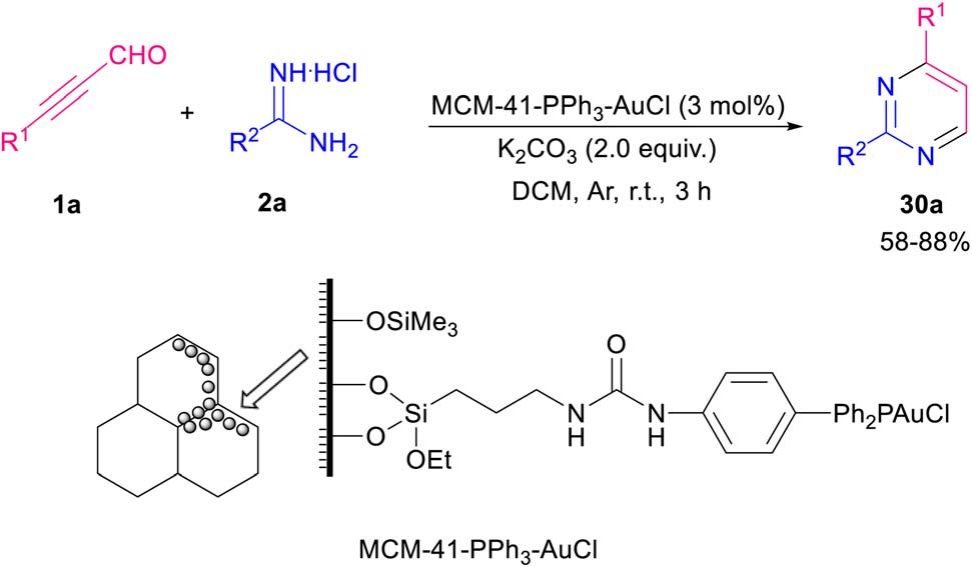
Synthesis of 2,4-disubstituted pyrimidines.

Amongst all heterocycles, pyrimidine shows a broad spectrum of biological activities.^48^ The core of pyrimidine is found in vitamin B1 and barbiturates. Pyrimidines are also used as hypnotics, like vernal. The bioactive structure pyrimidine has a vast therapeutic sketch as it is an essential component of natural composites, and chemotherapeutic drugs. This structure has been applied excellently against bacterial, malarial, viral, fungal, and cancerous contagions.^49^ In 2003, Bagley *et al.* proposed an effective microwave-assisted method for synthesizing pyrimidines 28b. In this new procedure, they used a variety of readily accessible alkynones 19a, and an excess of hydrochloride salt of either acetamidine, benzamidine, or guanidine 28a. These starting materials were stirred at 120 °C for 40 min in MeCN in the presence of Na_2_CO_3_ utilizing microwave irradiation at 90 W in a self-tunable microwave synthesizer (Scheme 38).^50^ NHCs are effective organocatalysts that promote a wide variety of Umpolung transformations of various aldehydes.^51^ Wang *et al.* developed a mild synthetic method *via* the NHC-catalyzed condensation of alkynyl aldehydes 1a, and amidines 2a (Scheme 39).^52^ In comparison with previous methods, this synthesis showed remarkable regioselectivity and a vast substrate scope and high functional group tolerance. An assumed mechanism is portrayed in Scheme 40. Alkynyl aldehydes 1a reacted with the NHC catalyst H to yield the intermediate B, then its oxidation produced the alkynyl acyl azolium intermediate C. Then, both *N*-substituted amidine 2a and alkynyl acyl azolium C activated by coordination to magnesium(ii). Micheal addition and following proton transfer results in intermediate F. Eventually, an intramolecular cycloaddition of F delivered the final product 28b. In 2019, Cai and Jiang reported the cyclization of 3-aryl/alkyl propiolaldehyde 1a and amidines 2a by using MCM-41-PPh_3_-AuCl as an effective recyclable catalyst. The reaction took place under mild conditions and resulted in 2,4-disubstituted pyrimidines 30a in moderate to high yields. The presence of heterogeneous gold(i) complex was essential for the reaction process (Scheme 41).^53^

**Scheme 42:**
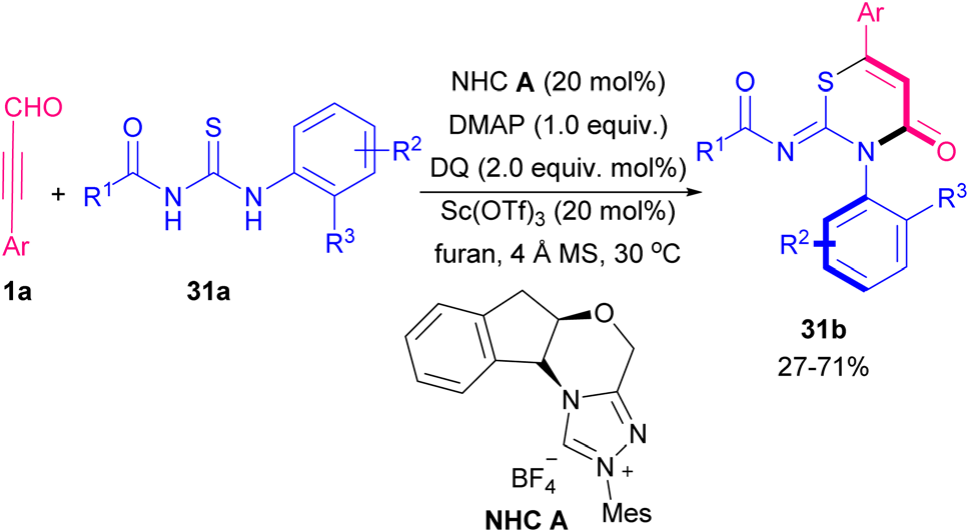
NHC-catalyzed synthesis of axially chiral thiazine derivatives.

**Scheme 43:**
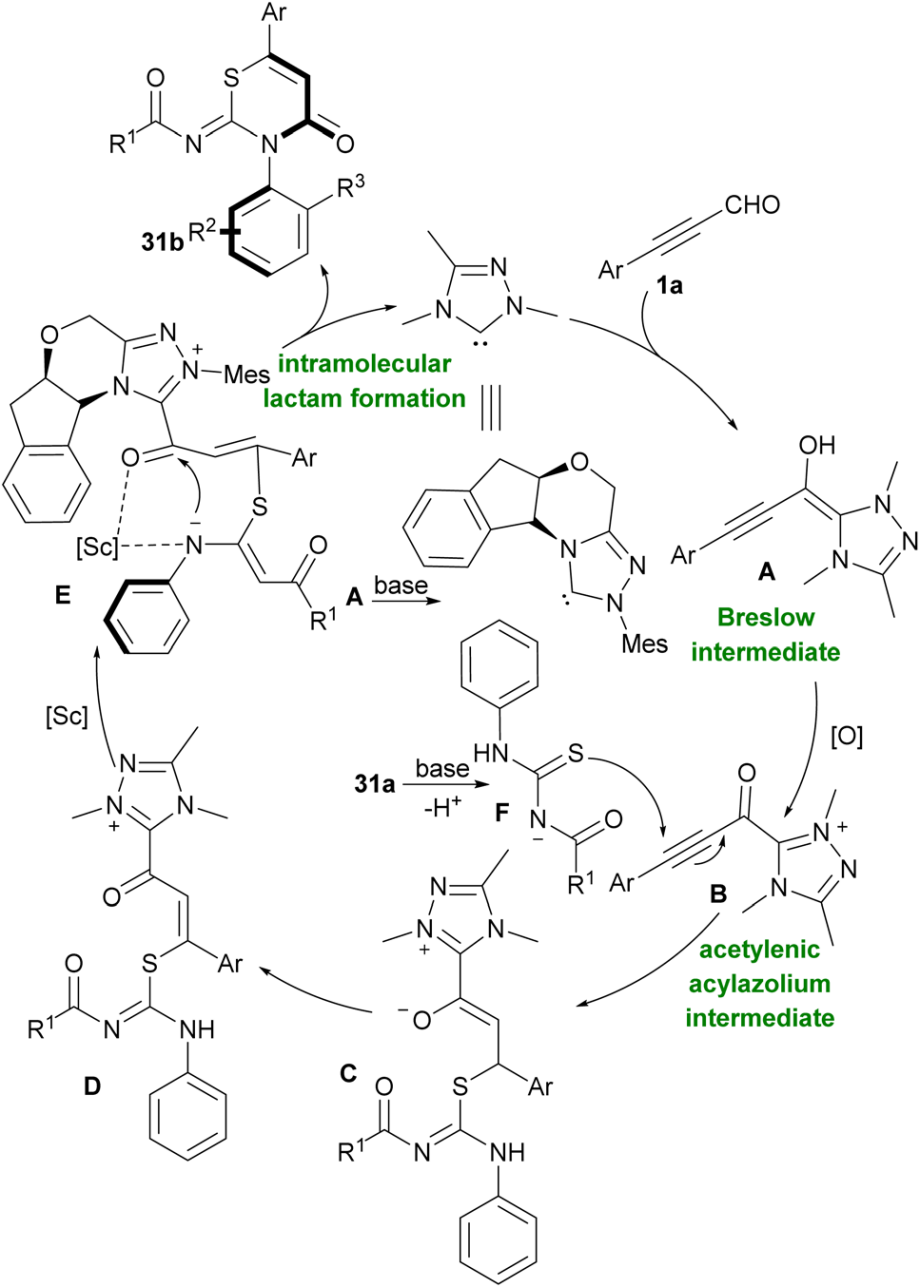
Proposed cyclic route for NHC-catalyzed synthesis of axially chiral thiazine derivatives.

**Scheme 44:**
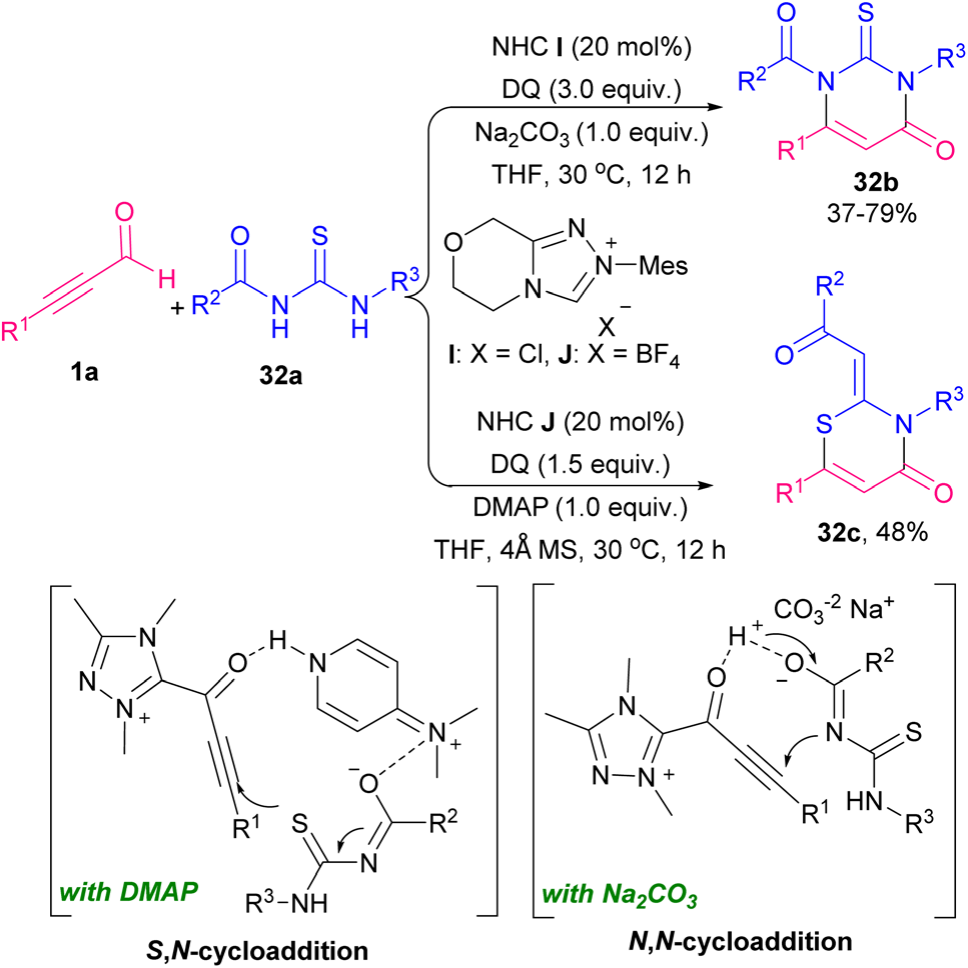
NHC-catalyzed synthesis of pyrimidinthione derivatives.

In 2021 Jin *et al.* explained the first example of a chiral NHC catalyst in the reaction of thioureas 31a and alkynyl aldehydes 1a to achieve axially chiral thiazine derivatives 31b with high optical purities (Scheme 42).^54^ The authors proposed a cyclic route for this atroposelective heteroatom cycloaddition involving a base promoted the formation of NHC catalyst A. Then, NHC A reacted with the alkynyl aldehyde 1a to afford the Breslow intermediate A. Acetylenic acylazolium intermediate B was created by the oxidation of A. Next, B reacted with F*via* a thio-Michael addition to rendering the adduct C, which led to the acylazolium intermediate D by a proton transfer. Interaction between the 2-isopropylbenzyl group in the substrate 31a and chiral NHC catalyst, triggered intermediate D under a selective intramolecular lactam generation to deliver thiazine 31b (Scheme 43). A similar transformation was reported by this research team in the application of thioureas 32a for [3 + 3] cycloaddition reaction with alkynyl aldehydes 1a using the NHC catalysts (Scheme 44).^55^ NHC-bounded acetylenic acylazolium intermediate acted as an electrophilic agent in this reaction. This work provided pyrimidinthione derivatives 32b with various substitutions. In addition, the authors showed two different pathways for cyclization by varying bases. *N*,*N*-Cycloaddition was observed in the presence of Na_2_CO_3_ while a *S*,*N*-cycloaddition product 32c was detected by changing the base into DMAP.

**Scheme 45:**
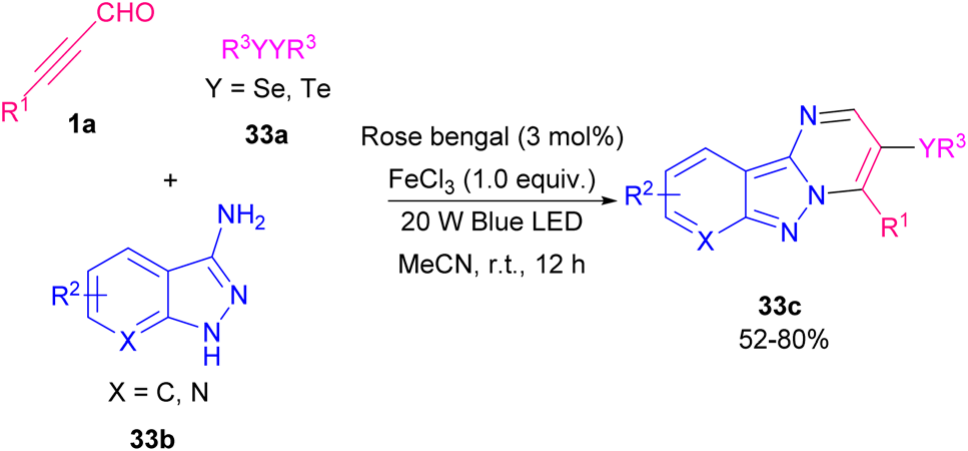
Blue LED-induced reaction between 3-amino indazoles, alkynyl aldehydes and chalcogenes.

**Scheme 46:**
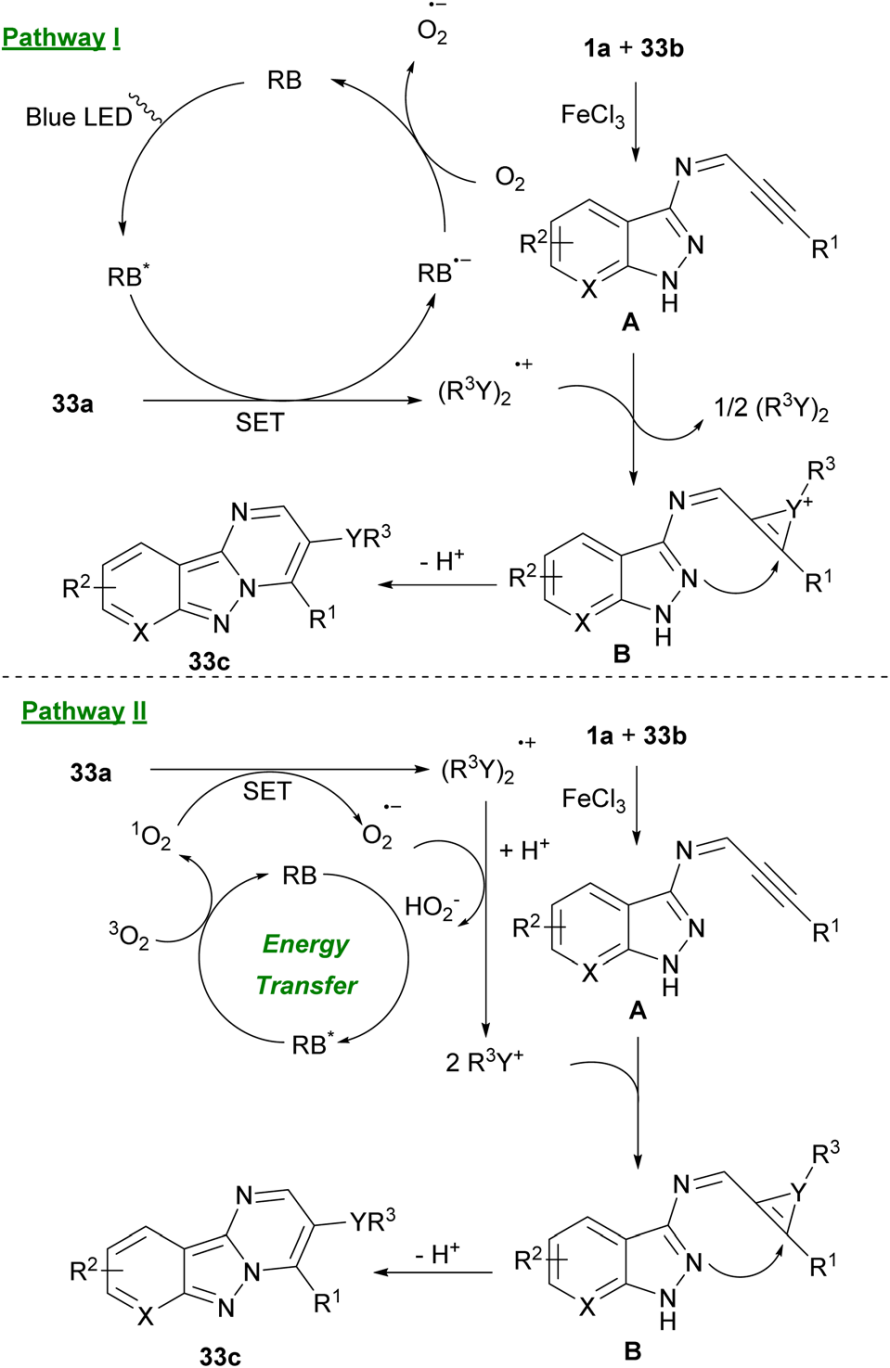
Two possible pathway for blue LED-induced reaction of 3-amino indazoles, alkynyl aldehydes and chalcogenes.

Liu and Cao *et al.* reported a green method for synthesizing selenium/tellurium-substituted pyrimido[1,2-*b*]-indazoles 33c from 3-amino indazoles 33b, alkynyl aldehydes 1a and chalcogenes 33a through a visible-light-promoted three-component reaction (Scheme 45).^56^ Two possible pathways for this synthesis are depicted in Scheme 33. In pathway I, RB was transformed to excited RB* *via* visible-light irradiation. Afterward, 33a underwent a SET process with RB* to create the (PhSe)_2_˙^+^ radical cation and the RB˙^−^ radical anion. Then, oxygen oxidized RB˙^−^ to the ground state, completing the photoredox cycle. On the other hand, FeCl_3_ promoted intermolecular condensation of 1a and 33b to form intermediate A. Intermediate A captured the (PhSe)_2_˙^+^ radical cation, resulting in cation B. Finally, intramolecular nucleophilic cyclization of cation B gave 33c. In pathway II, an energy-transfer process is likely involved in the reaction. RB converted to excited RB* under visible-light irradiation. Then, energy transfer between ground-state oxygen atom and RB* occurred to generate singlet oxygen, which underwent a SET reaction with diselenide 33a to form (PhSe)_2_˙^+^ radical cation. Radical cation (PhSe)_2_˙^+^ can be converted to intermediate PhSe^+^. At last, target product 33c was furnished *via* an electrophilic cyclization of A and PhSe^+^ (Scheme 46).

### 2.9. Synthesis of pyridones

**Scheme 47:**
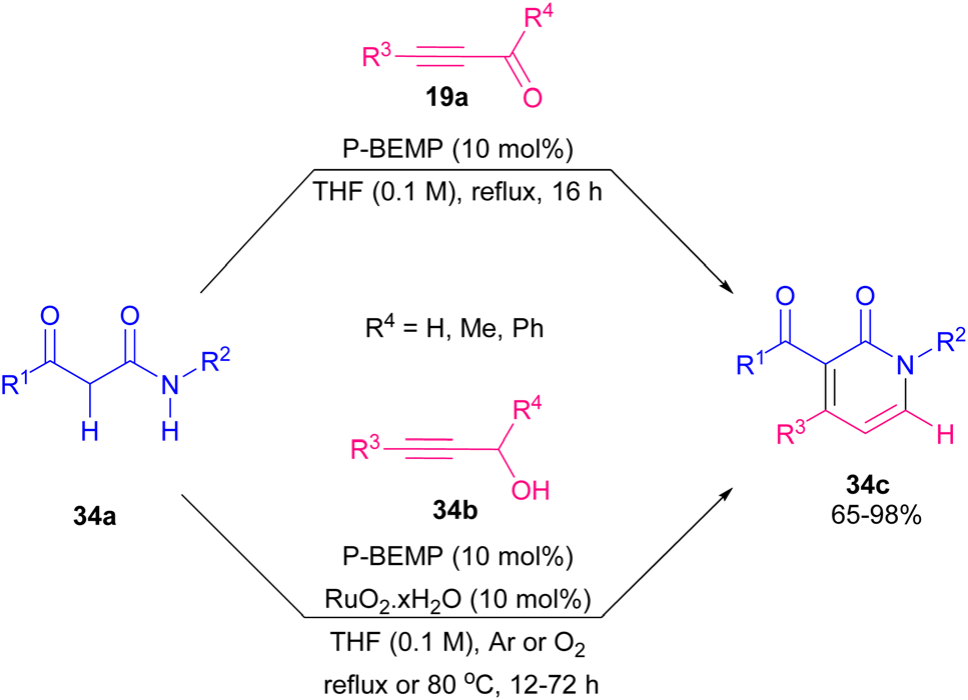
Synthesis of 2-pyridones from alkynyl aldehydes or alkynyl alcohols and β-keto amides.

**Scheme 48:**
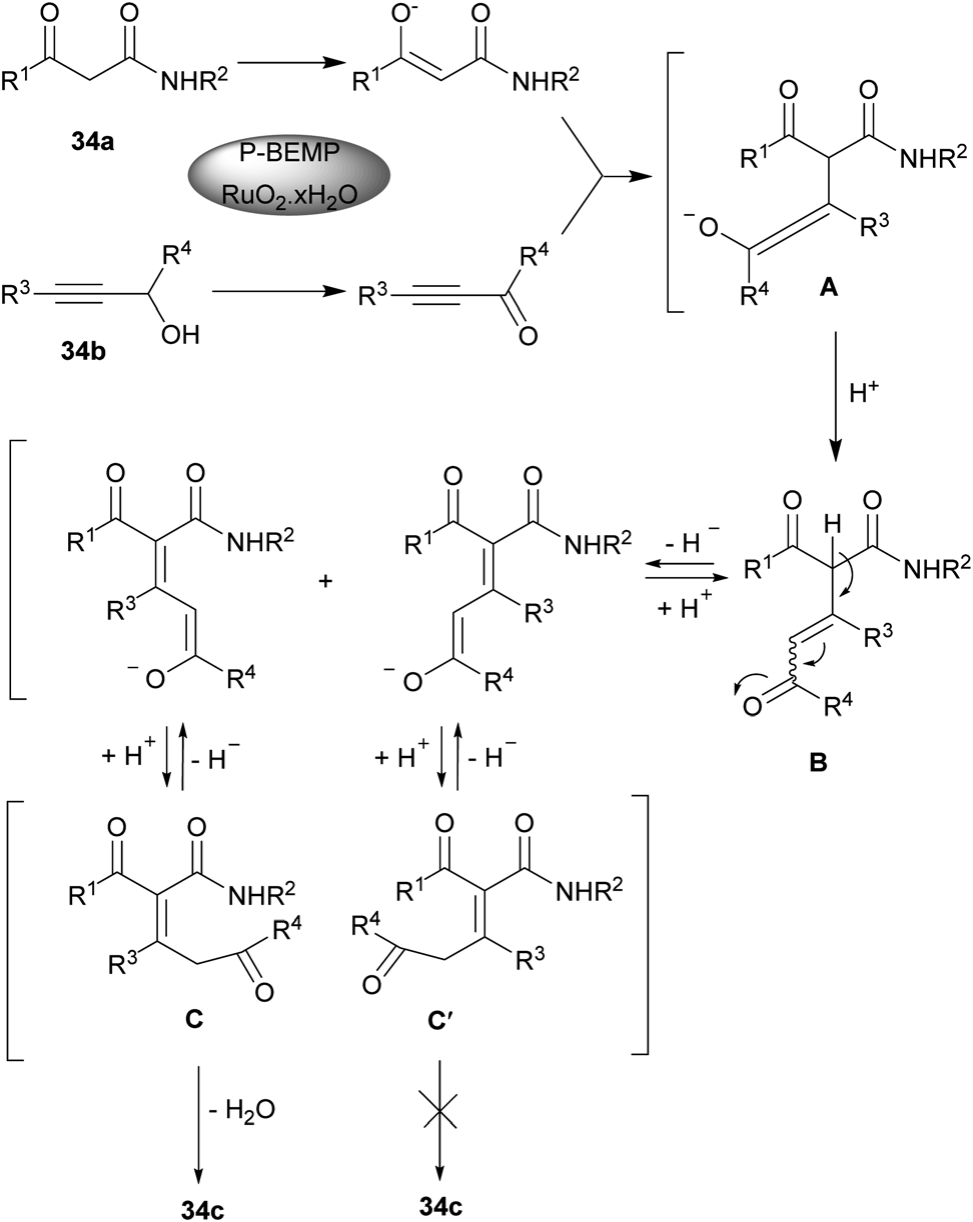
Suggested mechanism for synthesis of 2-pyridones.

**Scheme 49:**
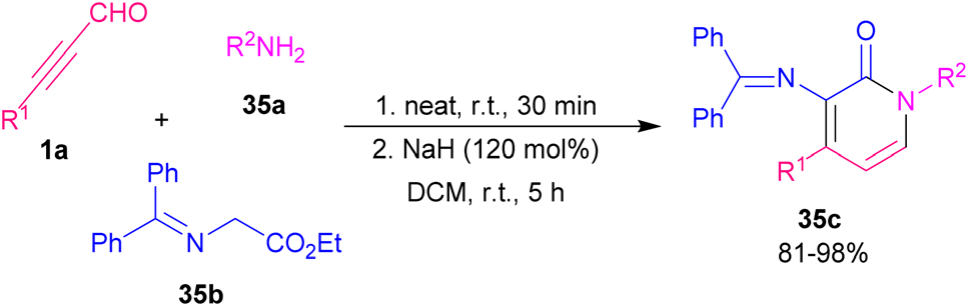
Three-component reaction of alkynyl aldehydes, amines and ethyl 2-(diphenylmethylene)amino acetate.

**Scheme 50:**
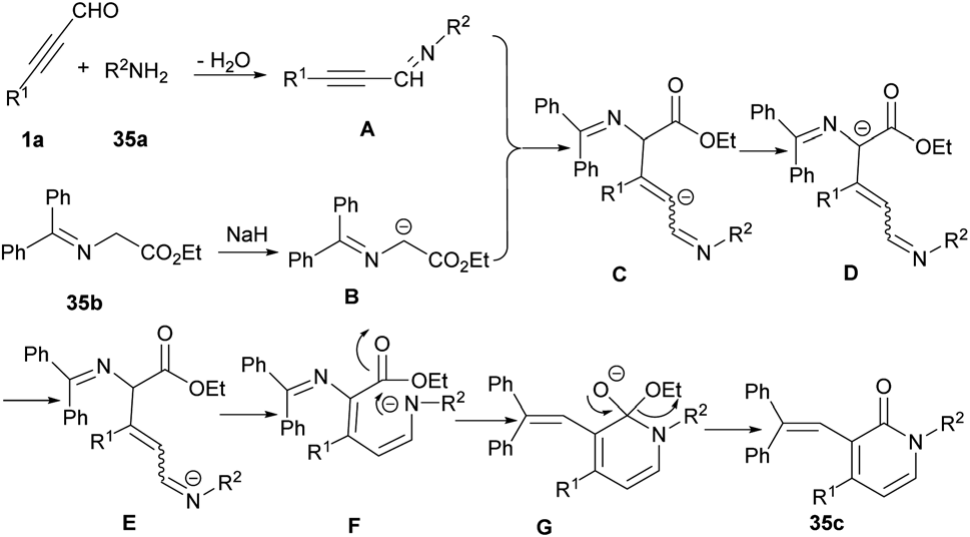
Plausible mechanism for the reaction of alkynyl aldehydes, amines and ethyl 2-(diphenylmethylene)amino acetate.

2-Pyridone scaffold is one of the essential structures found in various biological and natural compounds and its potent pharmaceutical and agrochemical activities provoke researchers to study it.^57^ Additionally, pyridone core is found in heterocycles which are important intermediates in the construction of alkaloid derivatives.^58^ Consequently, many methodologies have been developed for constructing 2-pyridones. In 2012 Rodriguez and Constantieux *et al.* succeeded in using alkynyl aldehydes 19a with β-keto amides 34a for constructing 2-pyridones 34c. Afterward, they can extend the substrate scope to alkynyl alcohols 34b instead related aldehydes (Scheme 47).^59^ β-keto amide 34a underwent the reaction with alkynyl alcohol 34b in refluxing THF for 12 hours using 10 mol% of P-BEMP (phosphazene-supported organocatalyst and RuO_2_·*x*H_2_O as an oxidizing catalyst. A possible mechanism is shown in Scheme 48. Apparently, the mixture RuO_2_·*x*H_2_O/P-BEMP sets off a domino reaction, allowing both *in situ* oxidation of the alkynyl alcohol 34b for construction of the transient amino enolate to generate the intermediate B by an intramolecular Micheal addition. At this level, proton transferred will drive the reaction by the selective cyclodehydration of diastereomer C. Lu *et al.* also developed a similar strategy for preparing 1,4-disubstituted 3-amino pyridone derivatives 35c through the three-component reaction of ynals 1a and amines 35a with ethyl 2-(diphenylmethylene)amino acetate 35b (Scheme 49).^60^ Sodium hydride deprotonated the active methylene proton of the 2-(diphenylmethylene)amino acetate 35b to produce intermediate B. Then, this intermediate was added to alkynyl imine A, which was formed by the reaction of 1a, and 35a to give C followed by proton transfer to afford compound D. Intermediate D underwent double bond migration and the consequent intramolecular aza-cyclization reaction of F led to the final product 35c (Scheme 50). Chen and Ye presented a simple procedure toward 2-pyridones from β-keto amides and alkynyl aldehydes through Micheal addition and intramolecular dehydration process under metal-catalyzed conditions.^61^ The reaction conditions were evaluated by varying solvent, base, and temperature. The best results were achieved by utilizing 30 mol% of K_2_CO_3_, which led to the highest yield.

### 2.10. Synthesis of quinolines

**Scheme 51:**
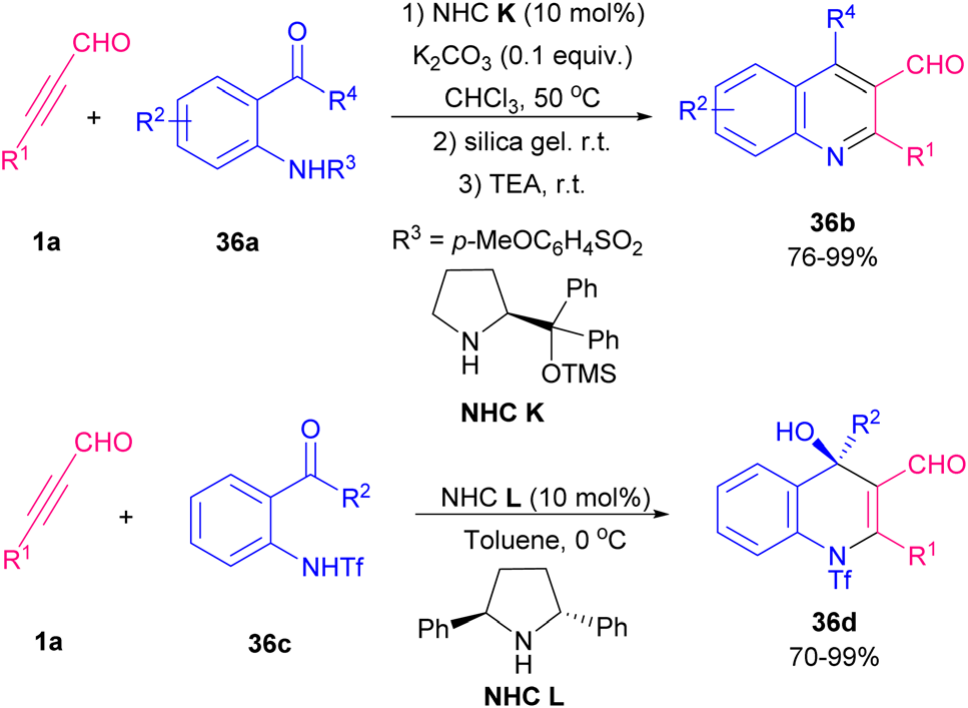
Reaction of *N*-tosyl-2-aminobenzaldehyde with alkynyl aldehyde.

**Scheme 52:**
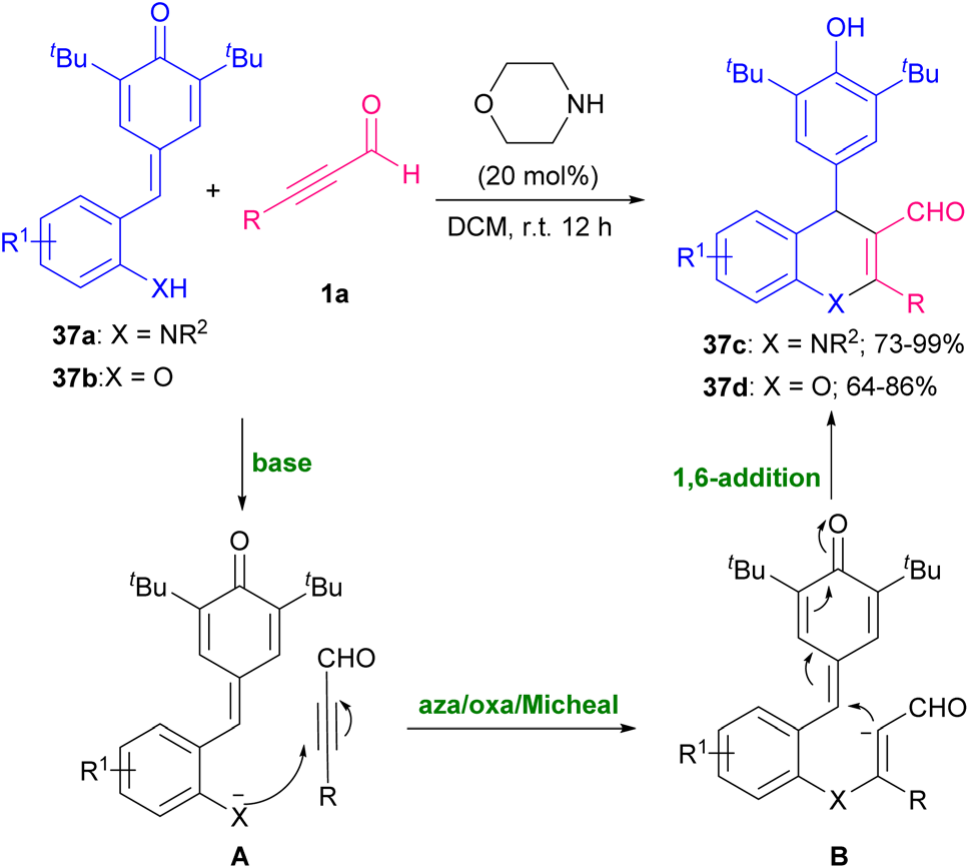
Synthesis of 1,4-dihydroquinolines and 4*H*-chromenes.

Quinoline is one of the most widespread heterocyclic compounds with potent industrial and medicinal applications. It is an essential moiety of both natural and synthetic compounds. This compound is used mainly as a central component for the synthesis of many drugs.^62^ For the synthesis of this sublime compound and its derivatives, numerous synthetic techniques have been reported. In 2012 Yu and Wang *et al.* investigated the reaction of *N*-tosyl-2-aminobenzaldehyde 36a with alkynyl aldehyde 1a by using diphenylprolinol TMS ether K (30 mol%) in CHCl_3_ at ambient temperature (Scheme 51).^63^ The tosyl(Ts) group was selected as a protecting group for the nitrogen atom, since its potent electron-withdrawing nature increased the acidity of the NH functionality, so facilitating ionization that generate a more nucleophilic nitrogen anion for primary Michael addition. Another use of organocatalysts in the construction of quinoline rings was proposed by Wang, Zhao and Hu *et al.* in 2020 (Scheme 52).^64^ In this regard, various organocatalysts, including pyrrolidine, piperidine, morpholine, triethylamine, *N*,*N*-diisopropylethylamine (DIPEA) and 1,8-diazabicyclo[5.4.0]undec-7-ene (DBU) and also inorganic bases, such as NaOH, Cs_2_CO_3_, and K_2_CO_3_ were examined. Among them, morpholine showed better results in [4 + 2] annulation reaction between *o*-tosylaminophenyl-*p*-QMs 37a and alkynyl aldehydes 1a. A series of 1,4-dihydroquinolines 37c were provided through aza/oxa-Michael/1,6-addition reactions. Moreover, *o*-hydroxyphenylsubstituted *p*-QMs 37b also exhibited good compatibility with this procedure and led to 4-aryl-4*H*-chromene products 57d.

**Scheme 53:**
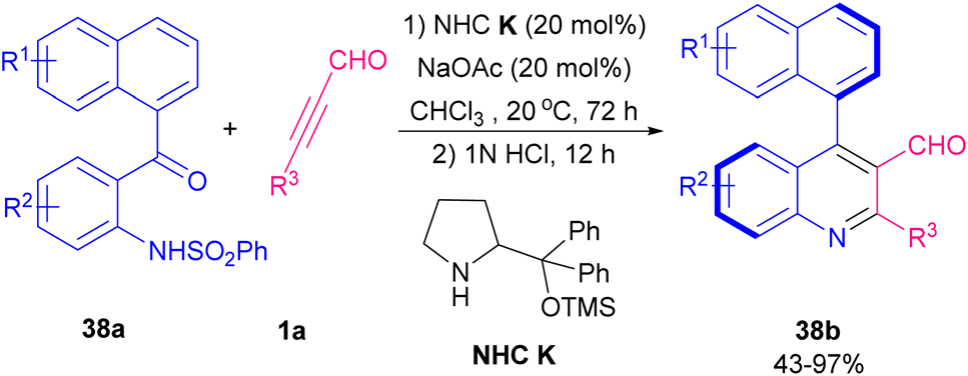
Aza-Michael/Aldol reaction of alkynyl aldehydes with *N*-(2-(1-naphthoyl) phenyl)benzenesulfonamides.

**Scheme 54:**
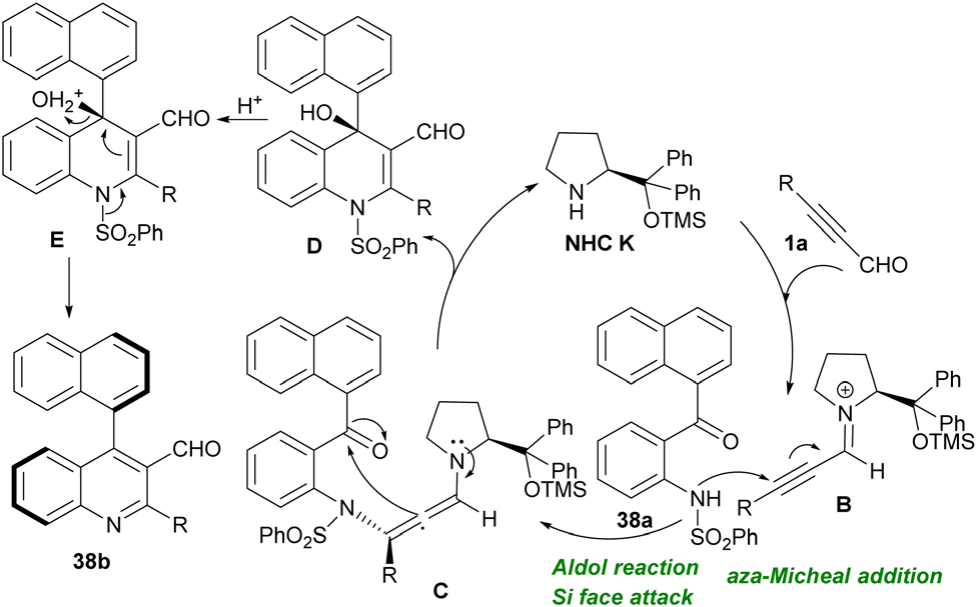
Suggested mechanism for aza-Michael/Aldol reaction of alkynyl aldehydes with *N*-(2-(1-naphthoyl) phenyl)benzenesulfonamides.

In 2021, Wang *et al.* revealed a NHC-catalyzed cascade aza-Michael/Aldol reaction of alkynyl aldehydes 1a with *N*-(2-(1-naphthoyl) phenyl)benzenesulfonamides 38a (Scheme 53).^65^ diverse axially chiral 4-naphthylquinoline-3-carbaldehyde structures 38b were acquired up to 97% chemical yield with high enantioselectivities (up to 96%). Besides, target chiral products could be transformed into chiral 4-naphthylquinolines with preserved enantioselectivities. Intrinsically, the alternative protocol to the preparation of chiral 4-naphthylquinolines can be anticipated. A possible reaction mechanism is depicted in Scheme 54. The reaction started *via* the nucleophilic addition of amine catalyst K to alkynyl aldehydes 1a, to form active iminium intermediate B. The aza-Michael addition of *N*-(2-(1-naphthoyl)phenyl)benzenesulfonamide 38a to iminium intermediate B gave the chiral intermediate C. After that, the nucleophilic attack of C onto ketone from *Si* face afforded a chiral dihydroquinoline intermediate D and regenerated catalyst K. Eventually, the protonation of D converted the OH group into OH_2_^+^ as a good leaving group. By a central-to-axial chirality conversion, the dehydration-deprotection of intermediate E delivered the axially chiral product 38b.

## 3. Synthesis of *O*-heterocyclic compounds

### 3.1. Synthesis of furans

**Scheme 55:**
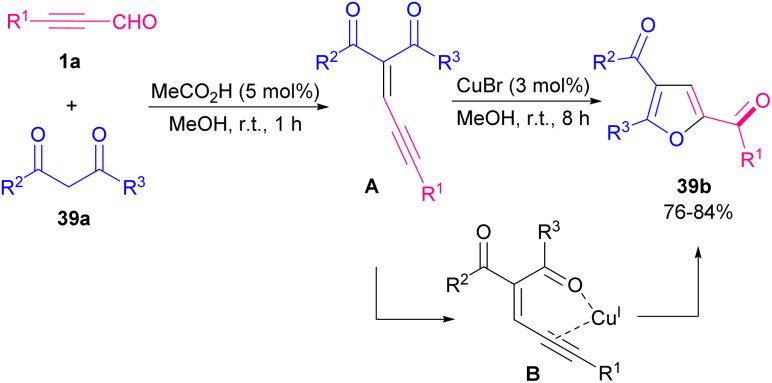
Visible-light-induced intermolecular [3 + 2] cycloaddition of indolizines.

**Scheme 56:**
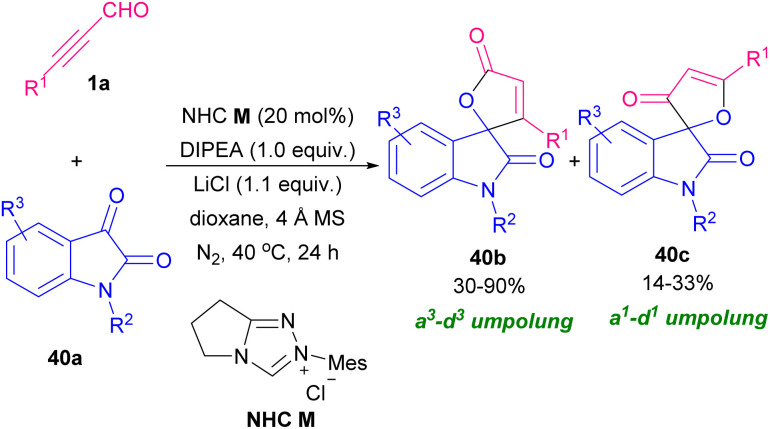
NHC/Lewis acid-mediated regioselective [3 + 2] annulation of alkynyl aldehydes with isatins.

**Scheme 57:**
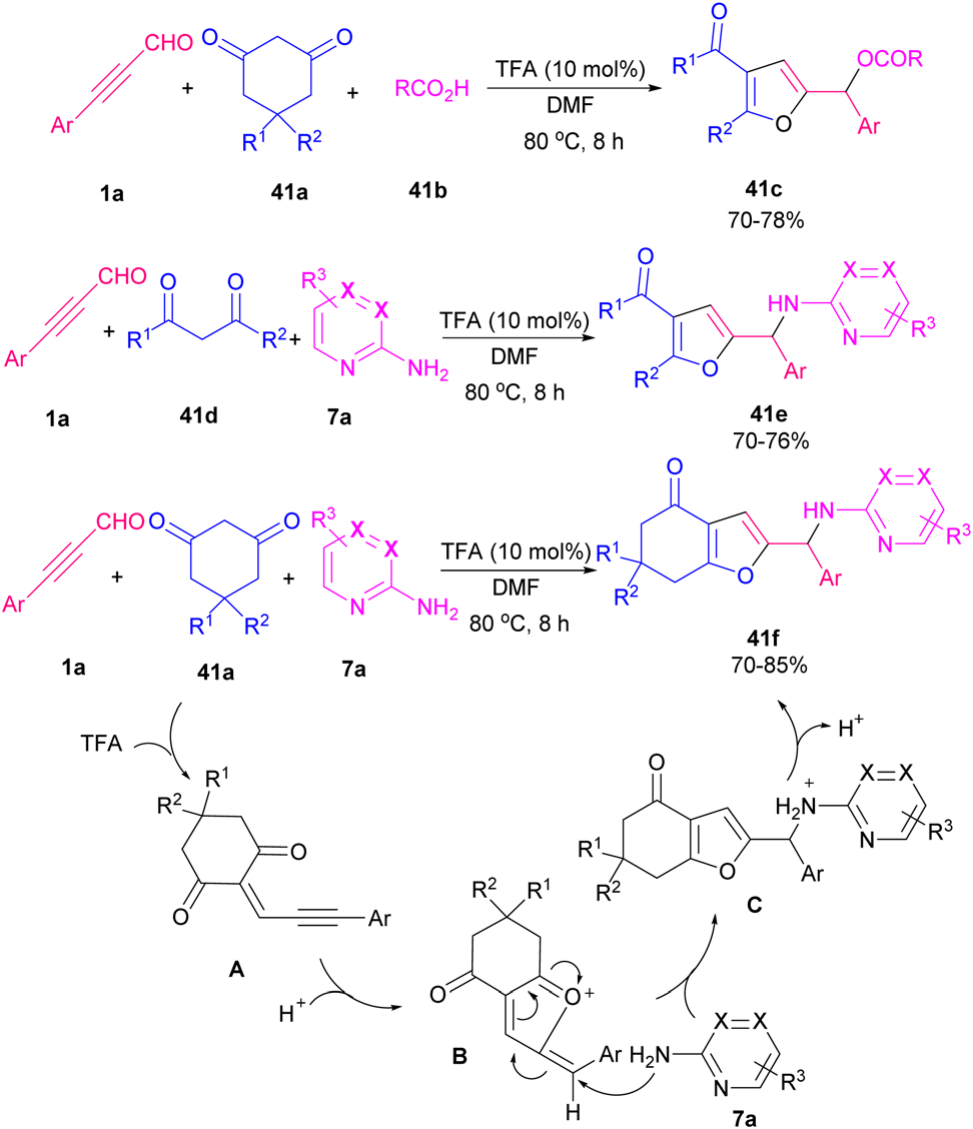
Synthesis of furans from aryl alkynyl aldehydes, 1,3-dicarbonyl compounds and aromatic amines or aliphatic acids.

In 2013, Cao, Jiang and co-workers constructed tri-substituted furan derivatives 39b using a copper catalyst in a one-pot manner, which opened the door for constructing different useful α-carbonyl furans using air as an external oxidant (Scheme 55).^66^ The reaction proceeded through the formation of intermediate A in the reaction mixture, followed by coordination with Cu(i) B, an intramolecular 5-exodig cyclization, and carbene oxidation by air oxygen. A cooperative NHC/Lewis acid-promoted regioselective [3 + 2] annulation of *N*-substituents isatins 40a with aryl alkynyl aldehydes 1a was performed toward oxindole core with furan-2-(5*H*)-one motif. The reaction proceeded in the presence of NHC M as an organocatalyst, LiCl as a Lewis acid, and *N*,*N*-diisopropylethylamine (DIPEA) as a base. By control of the reaction conditions, a^3^–d^3^ Umpolung products 40b were obtained, whereas in a few cases, a decrease in the reaction temperature resulted in a^1^–d^1^ Umpolung products 40c (Scheme 56).^67^ Synthesis of furan derivatives from readily available aryl alkynyl aldehydes 1a, 1,3-dicarbonyl compounds 41a and aromatic amines (pyridin-2-amine, pyrazin-2-amine, pyrimidin-2-amine) 7a or aliphatic acids 41b using trifluoroacetic acid (TFA) was reported by Cao *et al.* in 2016 (Scheme 57).^68^ The reaction began with Knoevenagel condensation of 1a and 41a toward intermediate A. Intermediate B was created by an intramolecular nucleophilic attack of the carbonyl oxygen under acidic conditions. By conjugate addition of 7a to B, and a dehydration process, target product 41f was obtained. In related work, the preparation of substituted furans was achieved through a three-component coupling of 1,3-dicarbonyl compounds, aryl/alkyl alkynyl aldehydes, and alkenes in the presence of 2 mol% ZnCl_2_.^69^

**Scheme 58:**
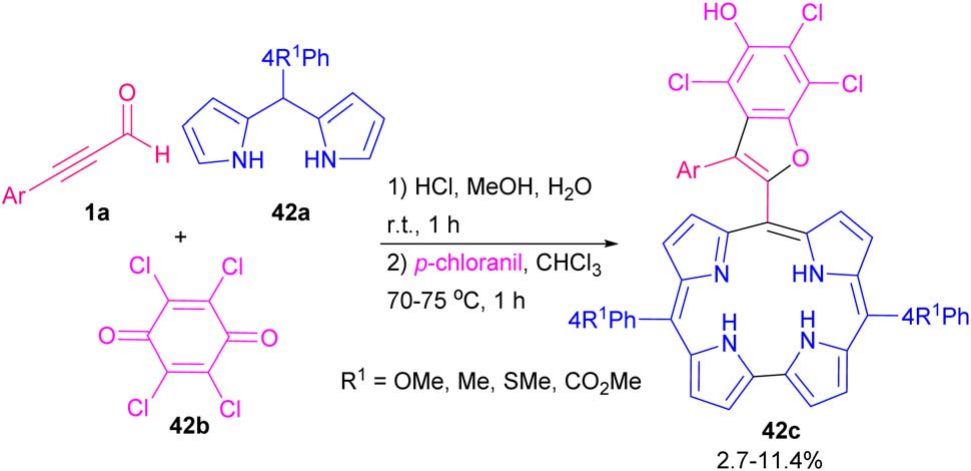
Synthesis of persubstituted benzofurans.

**Scheme 59:**
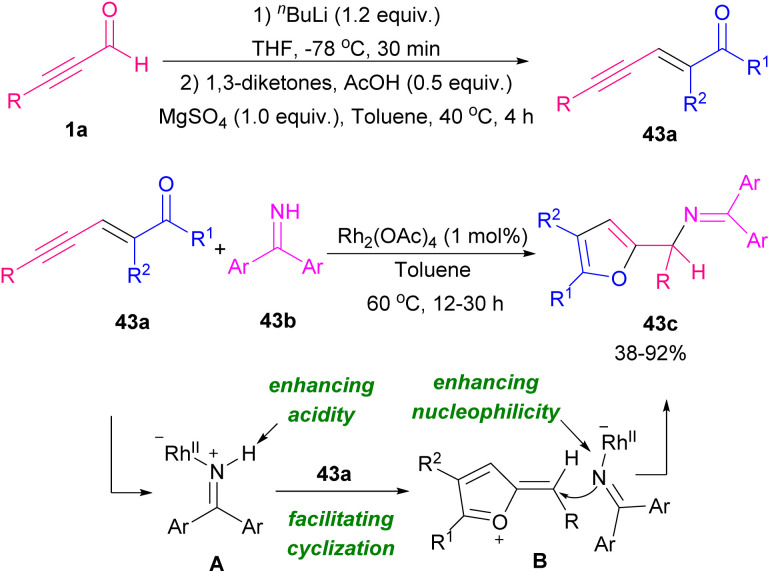
Rh-catalyzed synthesis of furan derivatives.

The corrole macrocycles showed good fluorescence features, which makes them potent candidate for cancer-targeting imaging and treatment.^70^ Aryl alkynyl aldehyde 1a can react with dipyrranes 42a, and *p*-chloranil 42b to form the corrole macrocycle bearing substituted benzofuran-2-yl 42c (Scheme 58).^71^ The presence of *p*-chloranil was essential in this three-segment reaction. Evaluating other oxidants, such as 2,3-dichloro-5,6-dicyano-1,4-benzoquinone (DDQ), *p*-benzoquinone, and bis(acetoxy)iodobenzene (BAIB) resulted in no product. Afterward, resulting products were investigated for UV-vis and fluorescence absorption. Recently, an atom-economical approach to the synthesis of furan derivatives starting from aryl alkynyl aldehyde was disclosed by Kim *et al.* (Scheme 59).^72^ In the procedure, at first, coordination of rhodium(ii) with nitrogen atom of diarylimine 43b occurred that enhanced N–H acidity. After the annulation of 43a to form cyclized intermediate B, the authors proposed a nucleophilic addition of Rh(ii)-iminyl complex A into B resulting in target product 43c.

### 3.2. Synthesis of pyranones

**Scheme 60:**
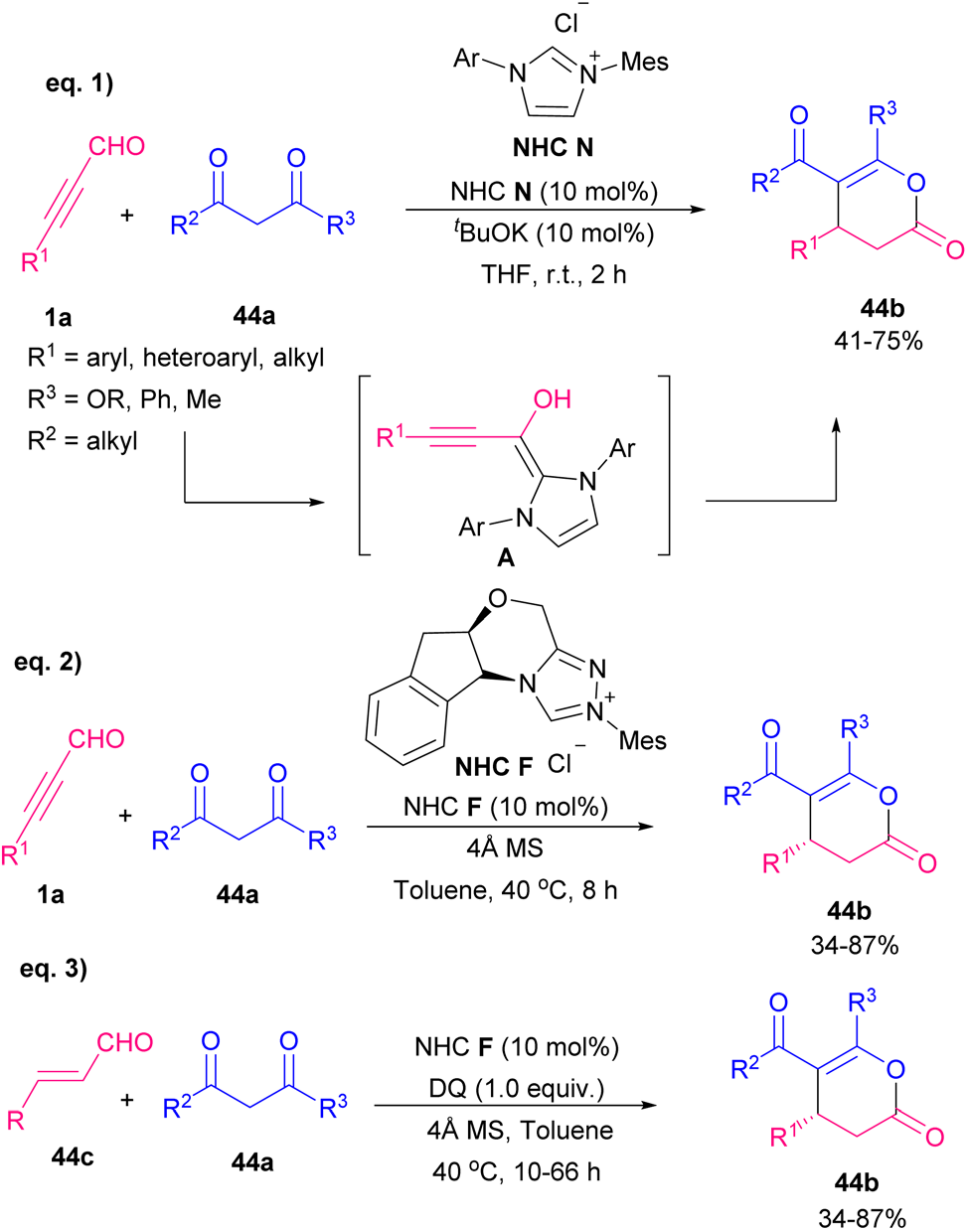
NHC-catalyzed synthesis functionalized dihydropyranones.

**Scheme 61:**
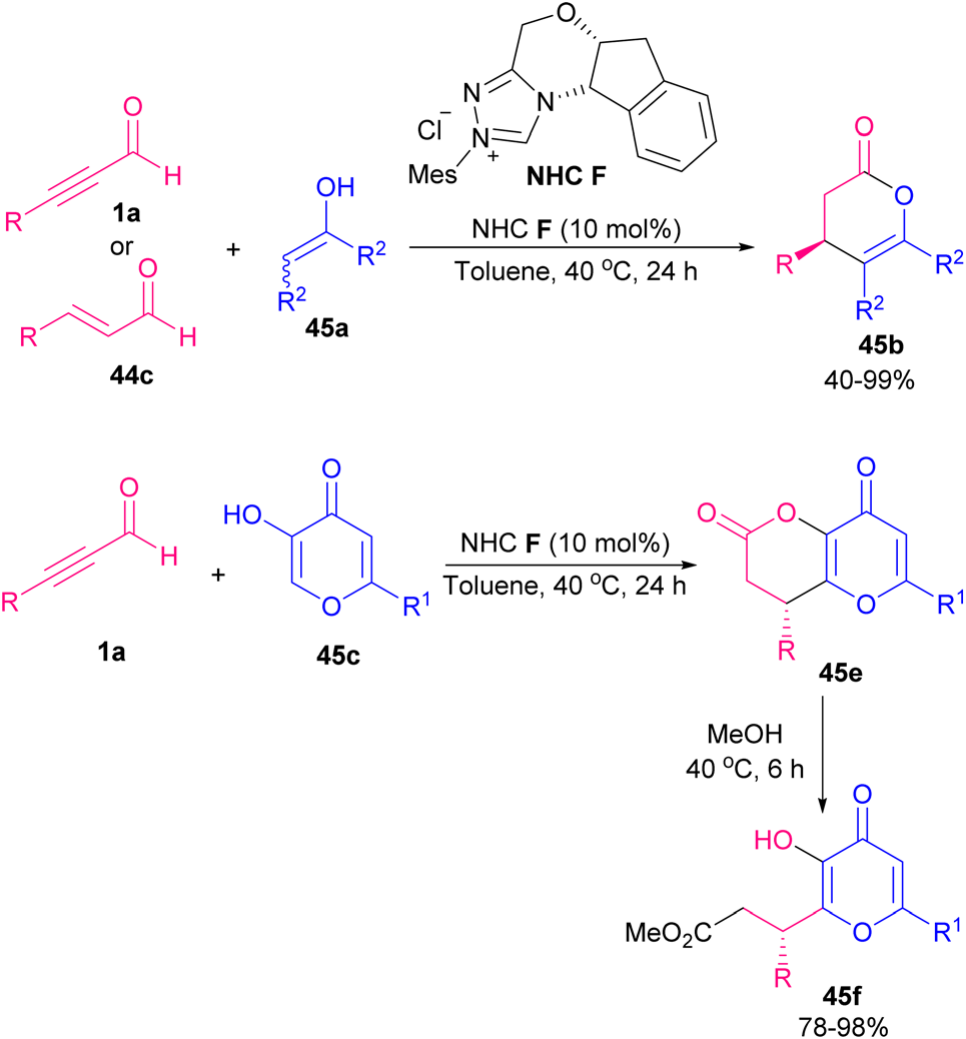
NHC-catalyzed annulation of ynals and enals with stable enols.

**Scheme 62:**
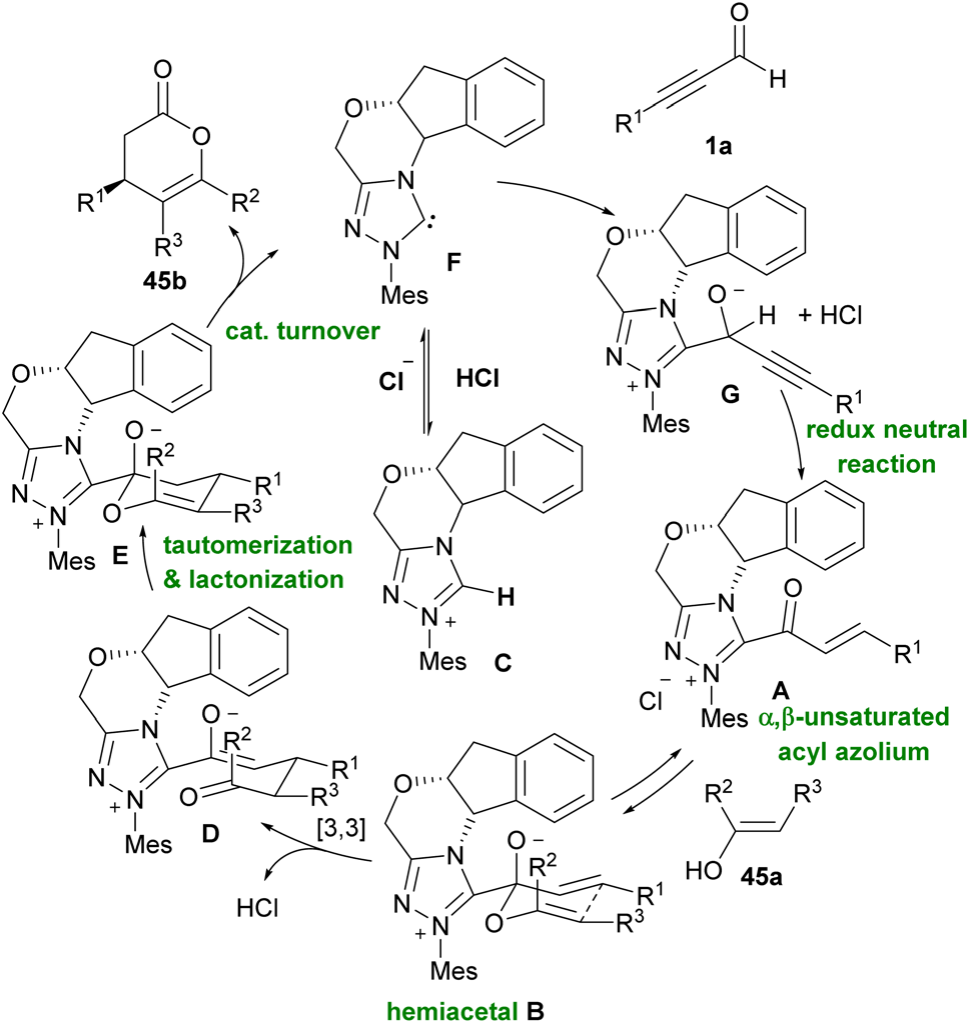
Proposed mechanism for NHC-catalyzed tandem Michael addition/lactonization.

Xiao and co-workers developed two similar NHC-catalyzed protocols for the synthesis of highly functionalized dihydropyranones (Scheme 60).^73^ In one work, alkynyl aldehydes 1a reacted with 1,3-diketones 44a or 1,3-keto esters through the formation of Breslow intermediate A alkynyl aldehydes 1a with catalyst NHC B (Scheme 60, eqn (1)). The reaction in the absence of base did not proceed. In other work, they use chiral NHC C to catalyze annulation between 1,3-dicarbonyls with alkynyl aldehydes 1a or alkenyl aldehydes 44c (Scheme 60, eqn (2) and eqn (3)). The reaction in the presence of molecular sieves showed higher yield and better stereoselectivity. In the case of alkenyl aldehydes 44c, the addition of quinone oxidant D modified the efficiency of the transformation. In 2012, Bode *et al.* prepared dihydropyranone scaffolds by using the chiral NHC F through an enantioselective Coates–Claisen rearrangement. For this purpose, they used ynals 1a and enals 44c and enols 45a as starting materials (Scheme 61).^74^ The key step in the reaction was most likely the formation of hemiacetal intermediate A. Mechanistic studies indicated that Claisen rearrangement on this intermediate was stereochemically determining step, which led to annulation reaction in competition with esterification. So, a cyclic pathway was proposed for this transformation. The deprotonation of triazolium salt precatalyst A by a base produced active NHC F, which added to ynals 1a to generate an adduct that underwent the proton transfer and the internal redox reaction to create α,β-unsaturated acyl azolium C. The species C and the enol 3 are in equilibrium with hemiacetal D, which underwent a [3,3]-sigmatropic rearrangement to produce adduct E. This was followed by tautomerization and lactonization to regenerate the catalyst and render dihydropyranone 45b (Scheme 62). In 2013, Du and Lu *et al.* in a related process provided 3,4-dihydropyrano[3,2-*b*]indol-2-ones. For this purpose, they employed 15 mol% of NHC catalyst, 1.1 equivalent of quinone oxidant, 4 Å MS, and a base for the annulation of alkynyl or alkenyl aldehydes with indolin-3-ones.^75^

**Scheme 63:**
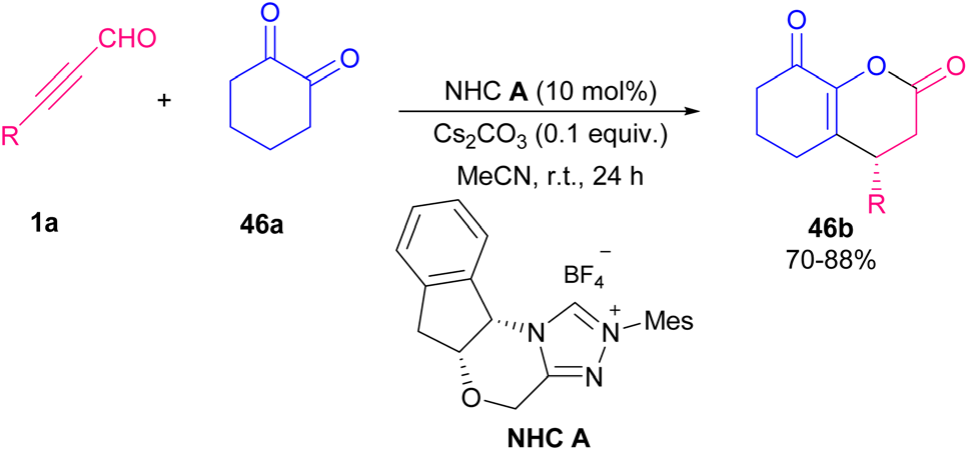
NHC-catalyzed tandem Michael addition/lactonization.

**Scheme 64:**
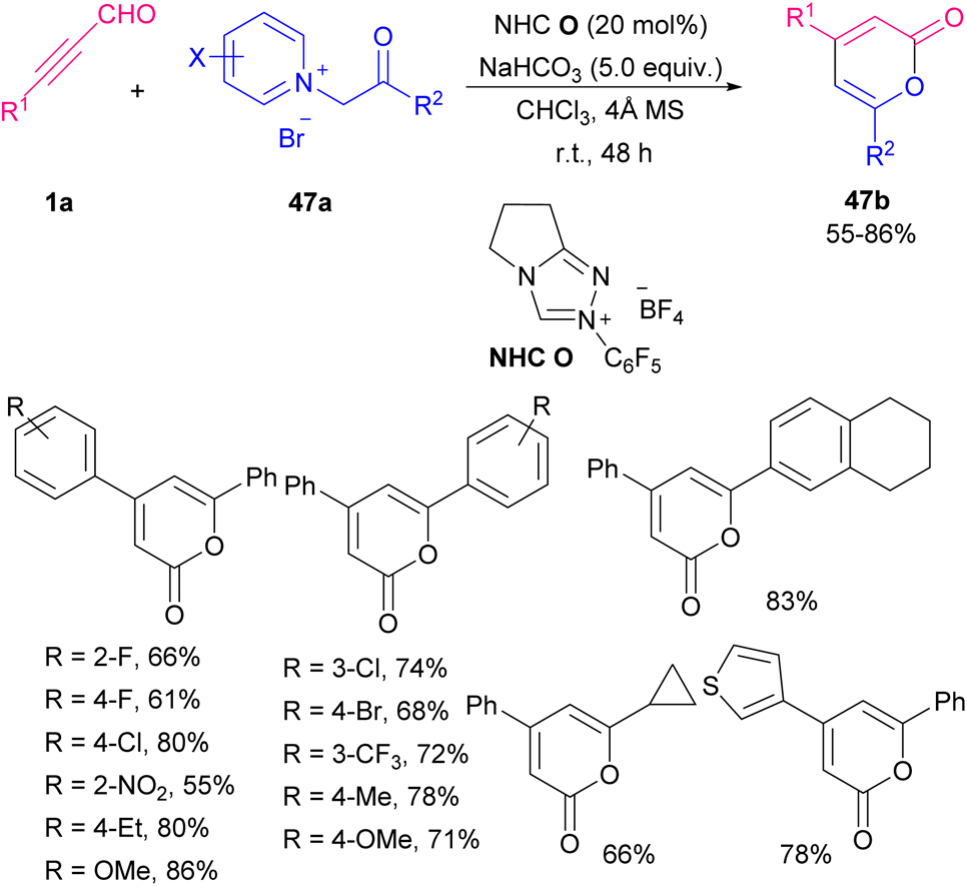
NHC-promoted the construction of 4,6-disubstituted α-pyrones.

Ren and Yuan *et al.* disclosed an approach toward chiral fused dihydropyranones 46b*via* NHC-catalyzed Michael addition/lactonization sequence of alkynyl aldehydes 1a with 1,2-diones 46a (Scheme 63).^76^ Among different kinds of organic bases (NMM, TBD/DIPEA, DMAP, TEA) and inorganic bases (K_2_CO_3_, Cs_2_CO_3_, KOH, KO^*t*^Bu), inorganic ones exhibited notable increase in chemical yield. After a while, Wang and co-workers reported a variant of the same cyclization reaction between aryl alkynyl aldehydes 1a with *N*-quinolinium, *N*-pyridinium, and *N*-imidazolium ylides 47a to construct biologically active 4,6-disubstituted α-pyrones 47b (Scheme 64).^77^

**Scheme 65:**
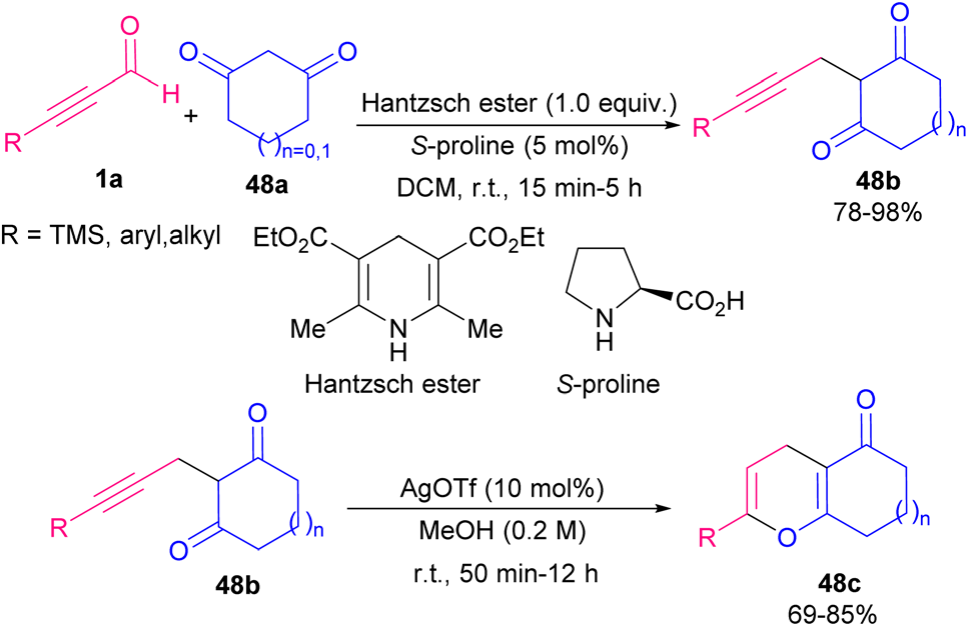
Three-component coupling reaction between ynals, cyclic-1,3-diketones and Hantzsch ester.

**Scheme 66:**
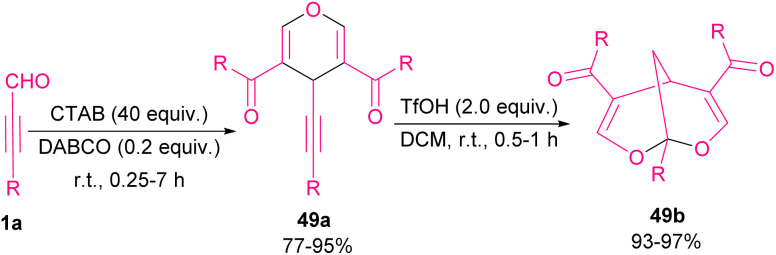
Synthesis of 4*H*-pyrans and 2,8-dioxabicyclo[3.3.1]nona-3,6-dienes starting from ynals.

**Scheme 67:**
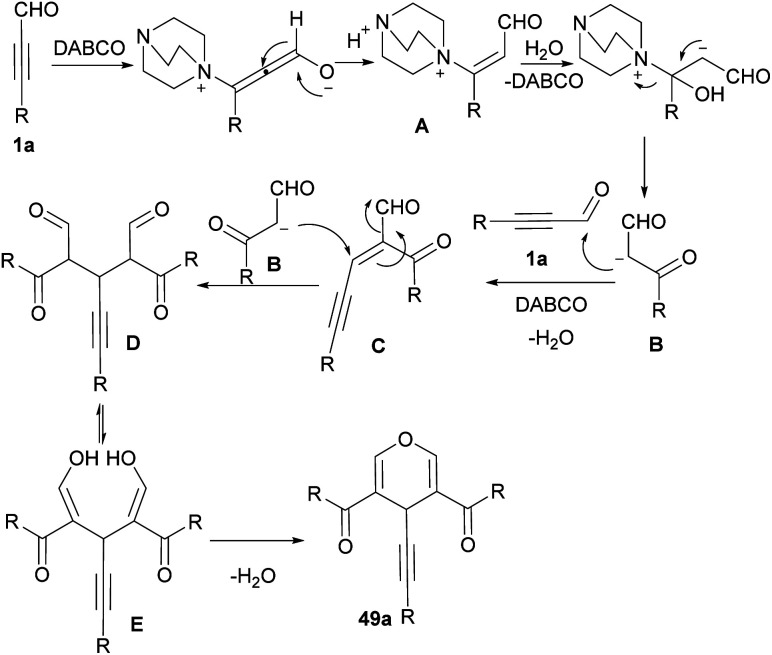
Possible mechanism for synthesis of 4*H*-pyrans.

In 2019, Ramachary *et al.* developed a three-component coupling reaction between ynals 1a, cyclic-1,3-diketones 48a and Hantzsch ester (Scheme 65).^78^ In this reaction, *S*-proline acted as an efficient amino acid catalyst for the chemoselective synthesis of 2-(3-aryl/alkylprop-2-yn-1-yl)cycloalkane-1,3-diones 48b in good to excellent yields. Afterward, by the addition of AgOTf as a catalyst, annulative esterification of 48b occurred to yield dihydropyran structures 48c. Green and sustainable synthesis of pyran derivatives from ynals 1a as starting materials access to 4*H*-pyrans 49a and then bioactive 2,8-dioxabicyclo[3.3.1]nona-3,6-diene scaffolds 49b was developed in 2021 (Scheme 66).^79^ In this method, trimerization of ynals 1a utilizing a cationic micelle like cetrimonium bromide (CTAB), and 1,4-diazabicyclo[2.2. 2]octane (DABCO) as a base in water as a solvent led to 4*H*-pyrans 49a. In the next step, obtained pyrans underwent a rearrangement process in the presence of 2.0 equivalent triflic acid (TfOH) in CH_2_Cl_2_ at room temperature to furnish 2,8-dioxabicyclo[3.3.1]nona-3,6-diene natural products 49b. The reaction started with Michael-type addition to ynal 1a to yield a zwitterionic allene intermediate A. Hereafter, Michael-type hydration and DABCO elimination in A led to β-keto aldehyde intermediate B. Aldol condensation between B and 9 rendered intermediate C. Afterward, C underwent second Michael-type addition with B to form intermediate D. Enolization of D to E and subsequent cyclodehydration produced 4*H*-pyran 49a (Scheme 67).

**Scheme 68:**
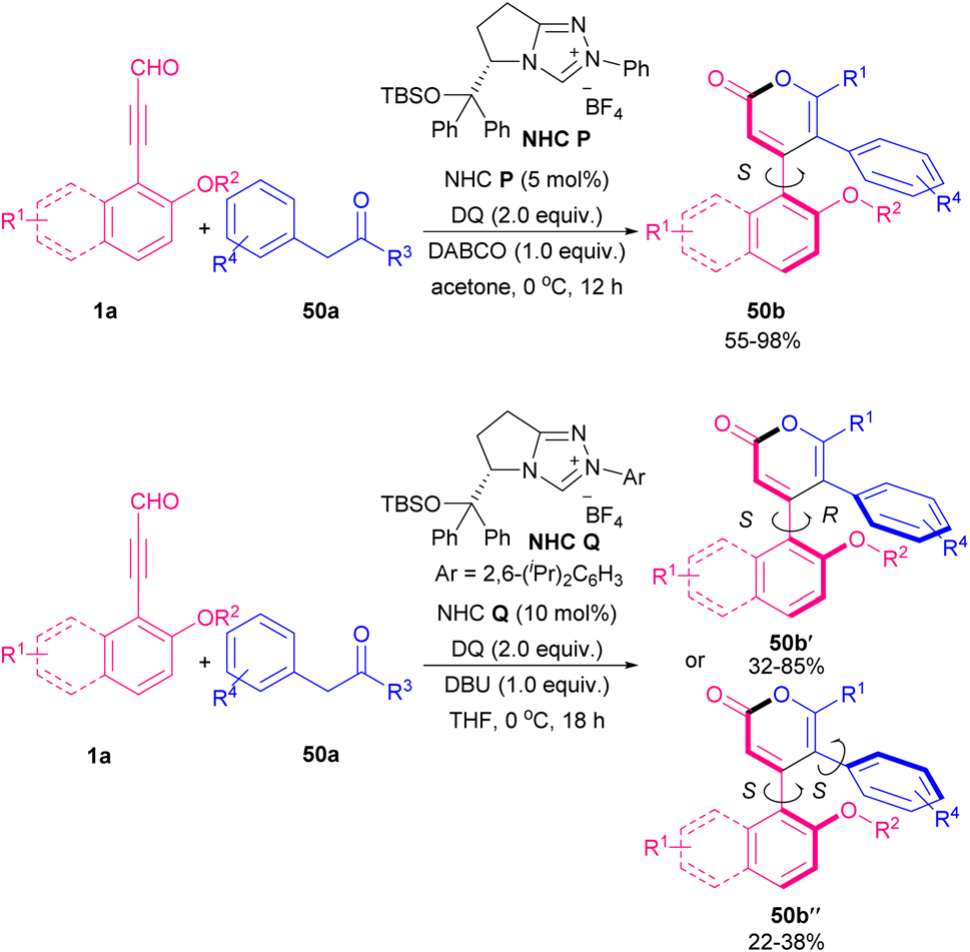
NHC-catalyzed synthesis of axially chiral triaryl-2-pyrones.

Very recently, Wong and Lu's research group introduced a protocol for the synthesis of axially chiral triaryl-2-pyrone frameworks by the employment of an oxidative organocatalysis system (Scheme 68).^80^ The products obtained *via* [3 + 3] annulation of alkynyl aldehydes 1a and 2-aryketones 50a, and contained one axis or two axes. After screening several NHC organocatalysts, chiral NHCs P, and Q were chosen for this transformation. The authors also investigated stereoisomer's energies, and rotational barriers by DFT calculation. Stereoisomer (*S*,*R*)-50b had the highest percentage (99.9%) compared to other stereoisomers.

## 4. Synthesis of *S*-heterocyclic compounds

### 4.1. Synthesis of thiophenes

**Scheme 69:**
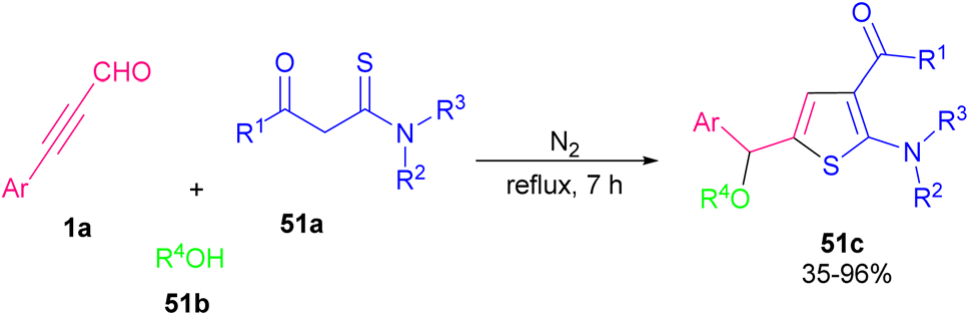
Aldol condensation/intramolecular cyclization/conjugate addition reaction of 2-ynals and thioamides.

**Scheme 70:**
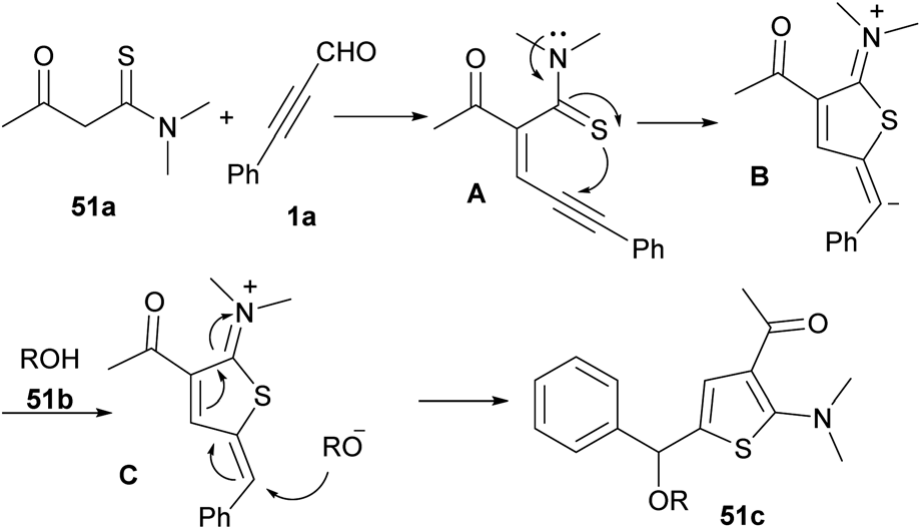
Plausible mechanism for aldol condensation/intramolecular cyclization/conjugate addition reaction of 2-ynals and thioamides.

Thiophene derivatives are important heterocyclic compounds and exhibit various properties and applications. Compounds containing thiophene ring system show several pharmaceutical properties, including anticancer,^81^ anti-inflammatory,^82^ antihypertensive,^83^ antimicrobial,^84^ and anti-atherosclerotic properties.^85^ In 2015 Luo and Deng and co-workers proposed an approach for the synthesis of functionalized 2-aminothiophene derivatives 51c (Scheme 69).^86^ They reported a simple, regioselective, and transition metal-free synthesis of multiubstituted 2-aminothiophenes 51c through a one-pot aldol condensation/intramolecular cyclization/conjugate addition cascade reaction of propargyl aldehydes 1a, thioamides 51a, and alcohols 51b in high yields. A possible mechanism for this transformation is depicted in Scheme 70. Aldol condensation of 1a with 51a produced the key intermediate A, which was exposed to spontaneous intramolecular cyclization and followed protonation to give intermediate C. The conjugate addition of alcohols 51b to intermediate C yielded the 2-aminothienyl ethers 51c.

### 4.2. Synthesis of thiopyrans

**Scheme 71:**
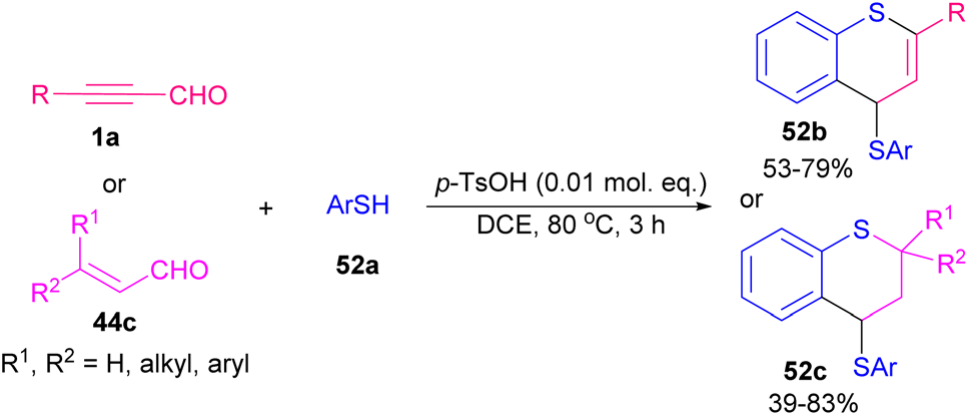
Intermolecular cycloaddition of α,β-unsaturated aldehydes with arenethiols.

**Scheme 72:**
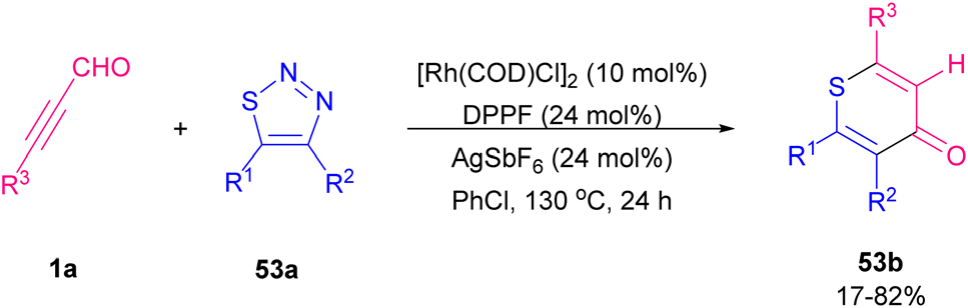
Rh-catalyzed reaction of 1,2,3-thiodiazoles with alkynyl aldehydes.

**Scheme 73:**
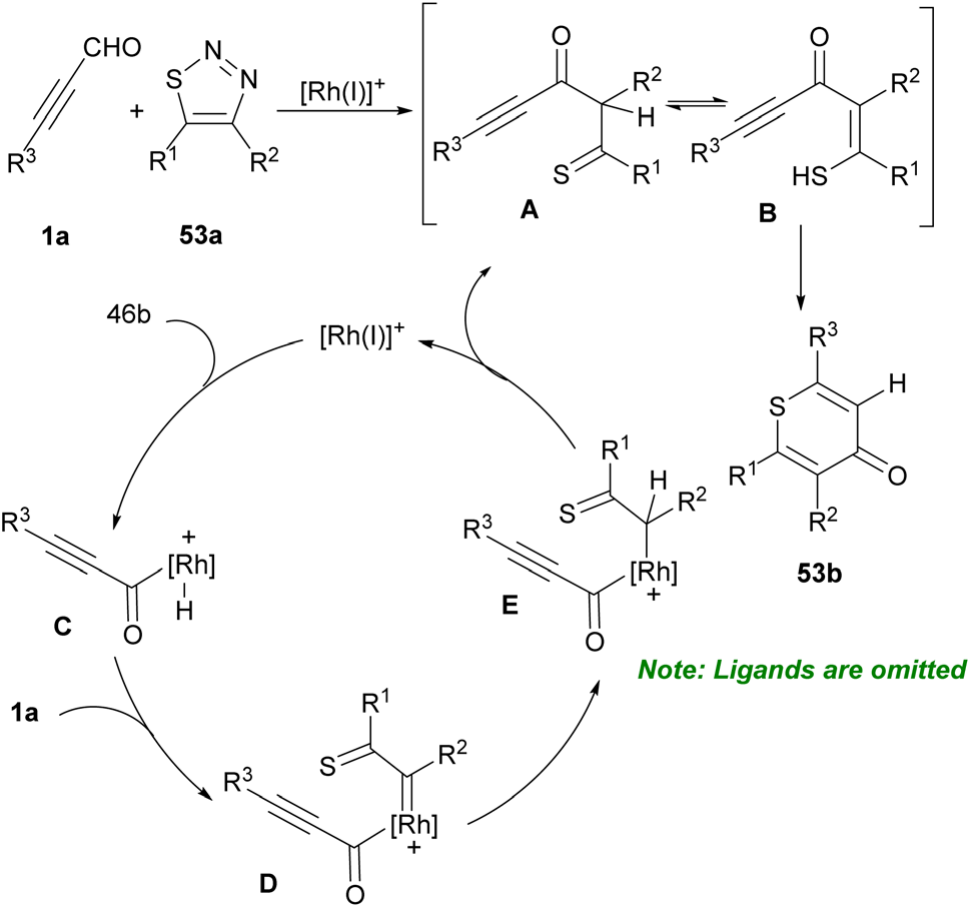
Tentative mechanism for Rh-catalyzed reaction of 1,2,3-thiodiazoles and alkynyl aldehydes.

In 2001, Ishino *et al.* developed a procedure for selective ring formation *via* intermolecular cycloaddition of α,β-unsaturated aldehydes 1a, and 44c and arenethiols 52a in the presence of a catalytic quantity of *p*-toluenesulfornic acid (*p*-TsOH) to afford the corresponding 4-thioaryl-2,3,4-trihydro-1-benzothiopyrans 52b, and 52c in good to excellent product yields (Scheme 71).^87^ This method is characterized by the employment of a catalytic amount of acids, an easy and convenient reaction procedure, mild conditions, and selective ring formation by readily available substrates, and good product yields. In 2019, Xu and Yang *et al.* found that 1,1-hydroacylation of thioacyl carbenes derived from 1,2,3-thiodiazoles 53a with alkynyl aldehydes 1a generated thioketones that can undergo 6-*endo*-dig cyclization to deliver [3 + 3] transannulation 4*H*-thiopyran-4-one products 53b as important subunits in a variety of advantageous compounds (Scheme 72).^88^ Similar transformations of alkenyl aldehydes also occurred to yield valuable products. In a tentative mechanism for 1,1-hydroacylation, oxidative addition of Rh(i) into alkynyl aldehydes 1a led to rhodium hydrides C, which further decomposed thiodiazoles 53a to obtain carbene complexes D. Migratory insertion of hydrido into carbenic center formed E, followed by reductive elimination to regenerate catalysts and gave 1,1-hydroacylation compounds 53b (Scheme 73).

## 5. Synthesis of O,N-heterocyclic compounds

### 5.1. Synthesis of isoxazolines, isoxazoles, and oxazoles

**Scheme 74:**
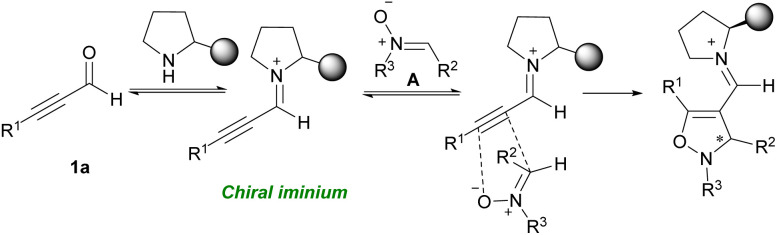
An iminium activation pathway for constructing chiral 4-isoxazolines.

**Scheme 75:**
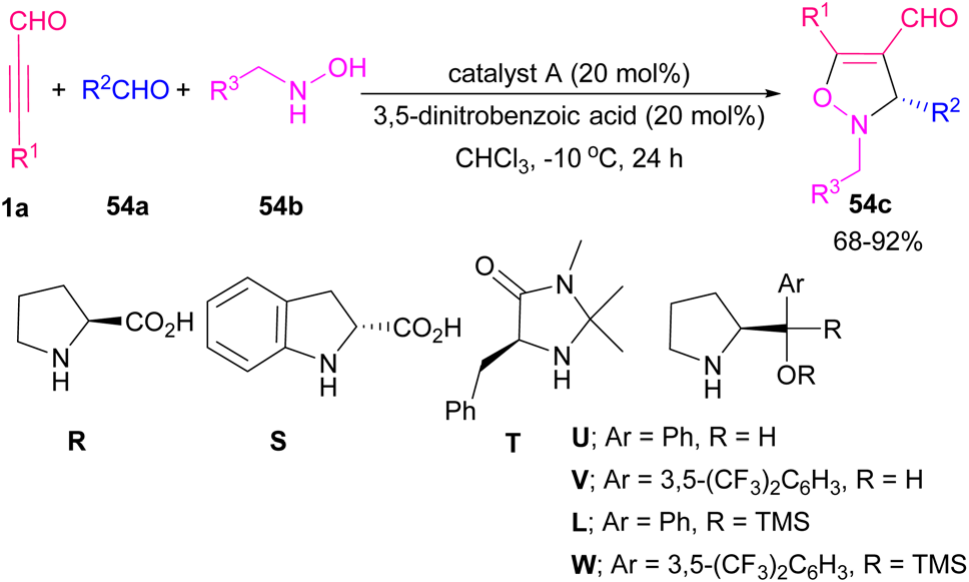
Use of organocatalyst for constructing chiral 4-isoxazolines.

**Scheme 76:**
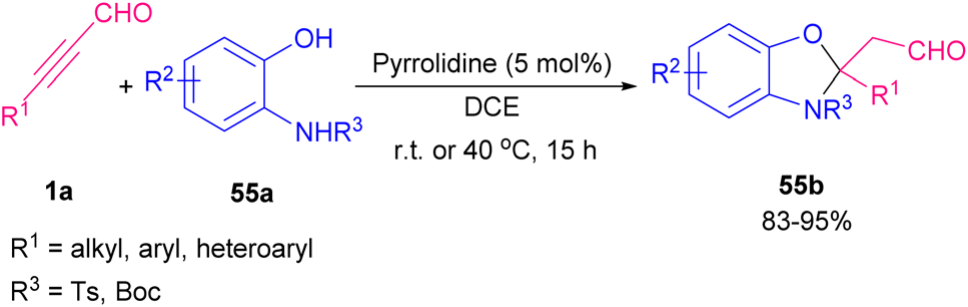
Amine-catalyzed [4 + 1] annulation for synthesize benzoxazoles.

An iminium activation pathway was described for enantioselective dipolar [3 + 2] cycloaddition between aryl/alkyl alkynyl aldehydes 1a and *in situ* generated nitrones A toward chiral 4-isoxazolines 54c (up to 96% ee) (Scheme 74).^89^ A series organocatalysts such as prolines R, and S, imidazolidine T, prolinols U, and V, and related ethers L, and W were utilized for this procedure. Among them, L-α,α-bis(3,5-ditrifluoromethylphenyl) prolinol W catalyzed the reaction in the presence of 3,5-dinitrobenzoic acid as an additive (Scheme 75). Meantime, the employment of pyrrolidines as the efficient catalysts to make isoxazoline rings from aryl/alkyl alkynyl aldehydes and arylnitrones was reported by Aleman and Fraile's research group.^90^ Pyrrolidine iminium activation procedure also used by Wang *et al.* for synthesizing biologically important benzoxazole 55b compounds from [4 + 1] annulation between 2-aminophenols 55a and alkyl/aryl alkynyl aldehydes 1a (Scheme 76).^91^

**Scheme 77:**
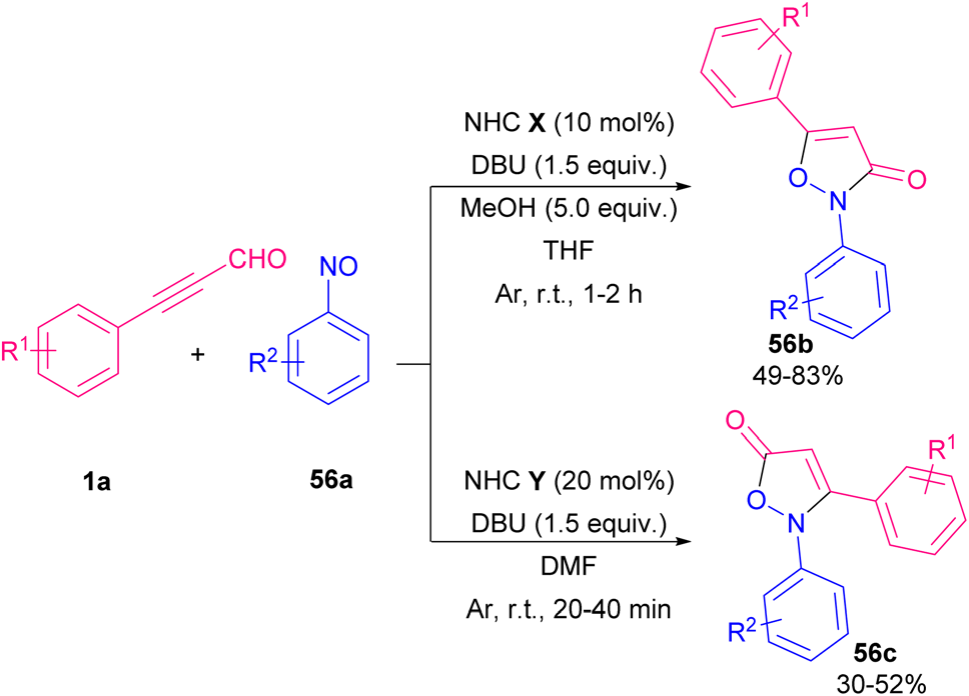
NHC-catalyzed [3 + 2] annulation of aryl alkynyl aldehydes with nitrosobenzenes.

**Scheme 78:**
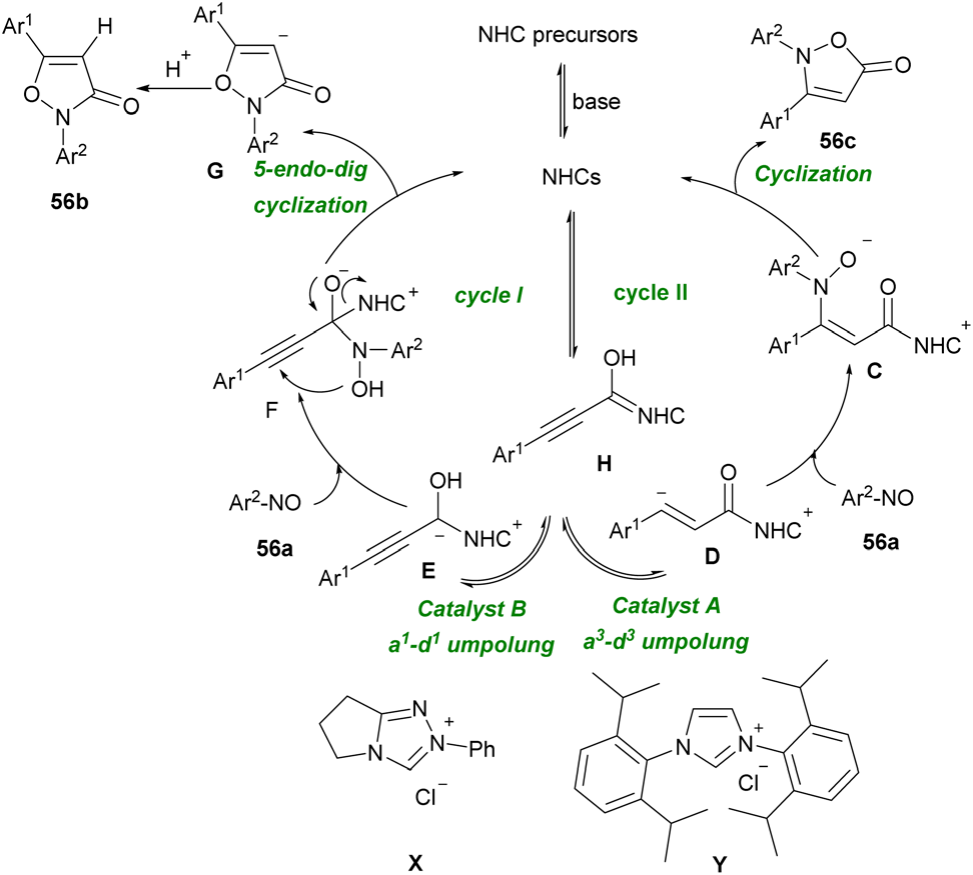
Proposed mechanism for NHC-catalyzed [3 + 2] annulation of aryl alkynyl aldehydes with nitrosobenzenes.

In 2013, She's research group developed a formal [3 + 2] annulation of aryl alkynyl aldehydes 1a and nitrosobenzenes 56a*via* C–O and C–N bond formation in the presence of NHC catalysis system (Scheme 77).^92^ A variety of 2,3-disubstituted isoxazol-5(2*H*)-ones 56b and 2,5-disubstituted isoxazol-3(2*H*)-ones 56c were prepared by this method. A mechanism for the regioselective Umpolung is illustrated in Scheme 78. In the presence of NHCs, intermediate H is generated. In cycle I, when catalyst B was used, acyl anion E formed, which reacted with 56a to form F. After releasing the NHC catalyst, product 56b was achieved. While in cycle II, the reaction of D with 56a resulted in the formation of C followed by a cyclization process to give product 56c.

**Scheme 79:**
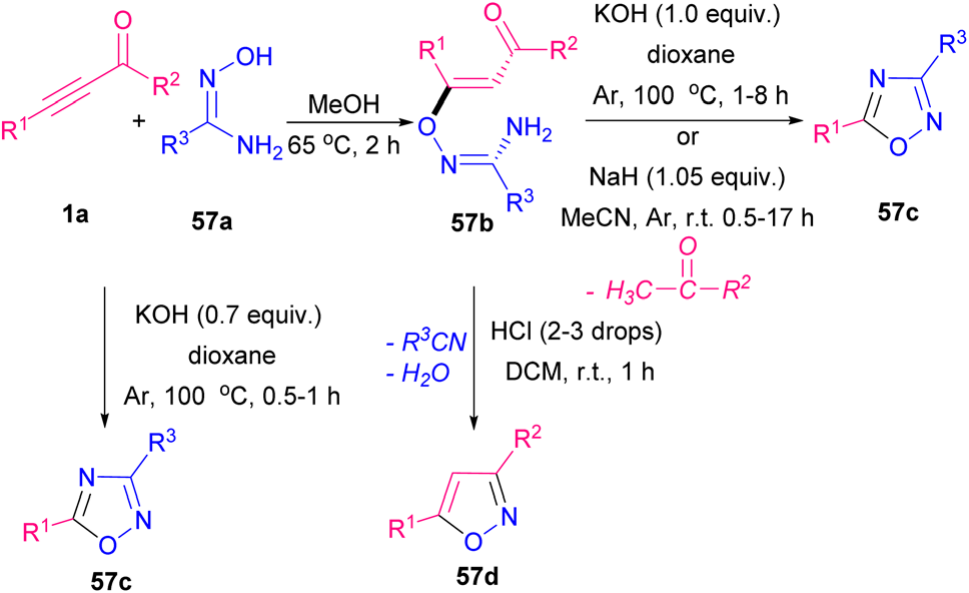
Construction of 1,2,4-oxadiazoles and isoxazoles.

A new strategy in metal-free constructing 1,2,4-oxadiazoles 57c and isoxazoles 57d was established by Zora and Kivrak (Scheme 79).^93^ The protocol employed amidoximes 57a and α,β-alkynic aldehydes 1a, or ketones as the starting materials to form conjugate addition products in the first step. Then, in the presence of KOH or NaH, 1,2,4-oxadiazoles 57c and isoxazoles 57d were obtained *via* a ketone group elimination. It is found that 1,2,4-oxadiazoles 57c also could synthesize using a base in one step. The transformation also proceeded by the addition of 2–3 drops of HCl, which after nitrile and water release, isoxazole rings were furnished.

**Scheme 80:**
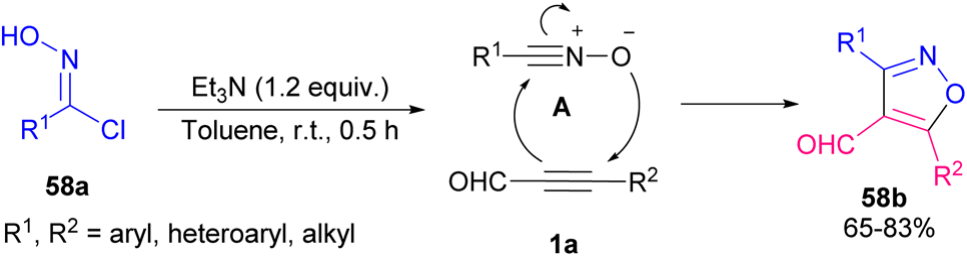
Construction of 1,2,4-oxadiazoles and isoxazoles.

**Scheme 81:**
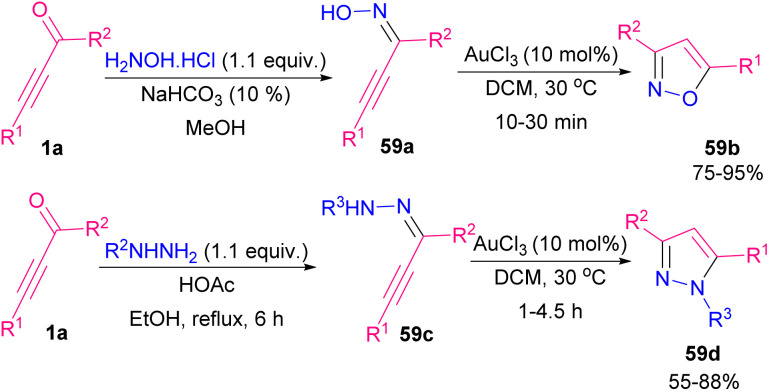
Preparation of isoxazoles and pyrazoles.

**Scheme 82:**
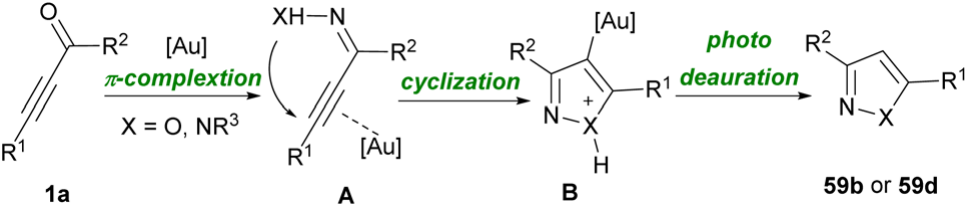
Possible mechanism for preparing isoxazoles and pyrazoles.

**Scheme 83:**
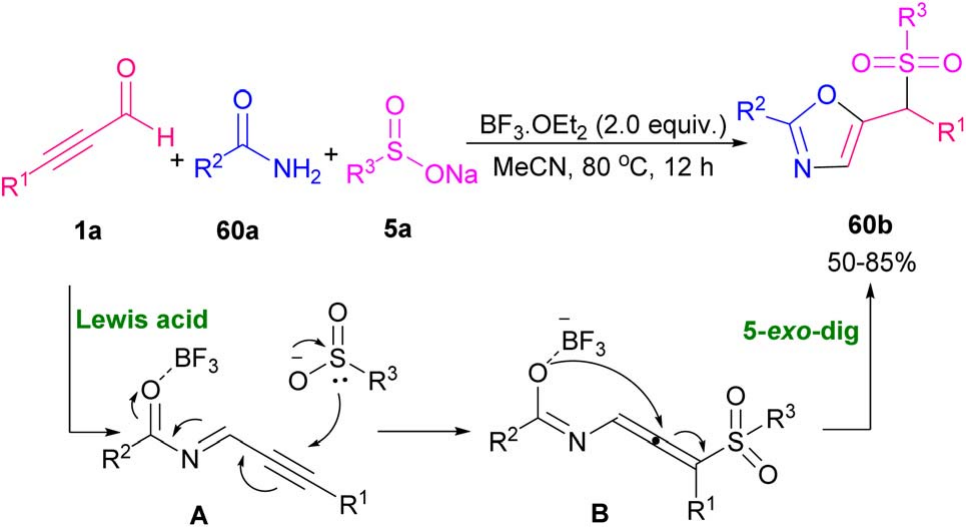
Lewis acid-promoted synthesis of oxazole derivatives.

Reactive nitrile oxides A can be generated *in situ* from chloro-oximes 58a to participate in [1,3]-dipolar cycloaddition with α,β-acetylenic aldehydes 1a toward 1,2-oxazols 58b. Both alkynyl aldehydes 1a and oximes 58a bearing aryl, heteroaryl and alkyl groups were compatible in this transformation (Scheme 80).^94^ Preparation and cytotoxic evaluation of isoxazoles 59b and pyrazoles 59d were performed by Praveen *et al.* (Scheme 81).^95^ In this regard, they investigated the capability of AuCl_3_ in the cycloisomerization of α,β-acetylenic oximes 59a and α,β-acetylenic hydrazones 59c. The reaction initiated by the activation of alkyne 1a to generate π-complex A. Meantime, an intramolecular nucleophilic attack of OH or NHR^3^ resulted in a cyclization process, in which proto-deauration of B led to producing 59b or 59d (Scheme 82). They also evaluated the cytotoxic potential of selected products toward cancer cells. Very recently, another interesting type of Lewis acid catalyzed the construction of 2,5-disubstituted oxazoles 60b was reported by Zhu and Cao *et al.* (Scheme 83).^96^ Broad substrate scope including propargyl aldehydes 1a, aromatic amides 60a, and aryl/alkyl-substituted sodium sulfonates 5a worked well in this metal-free cyclization reaction. The reaction proceeded *via* the formation of intermediate A, the addition of 5a into A, and 5-*exo*-dig cyclization.

**Scheme 84:**
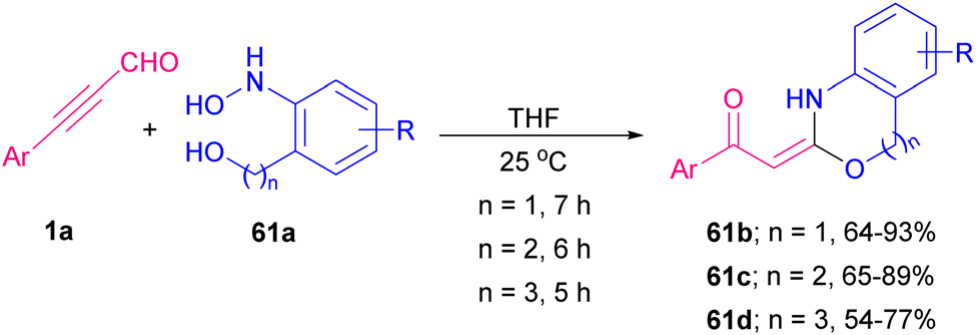
Synthesis of benzo-oxazine, benzo-oxazepine and benzo-oxazocine.

**Scheme 85:**
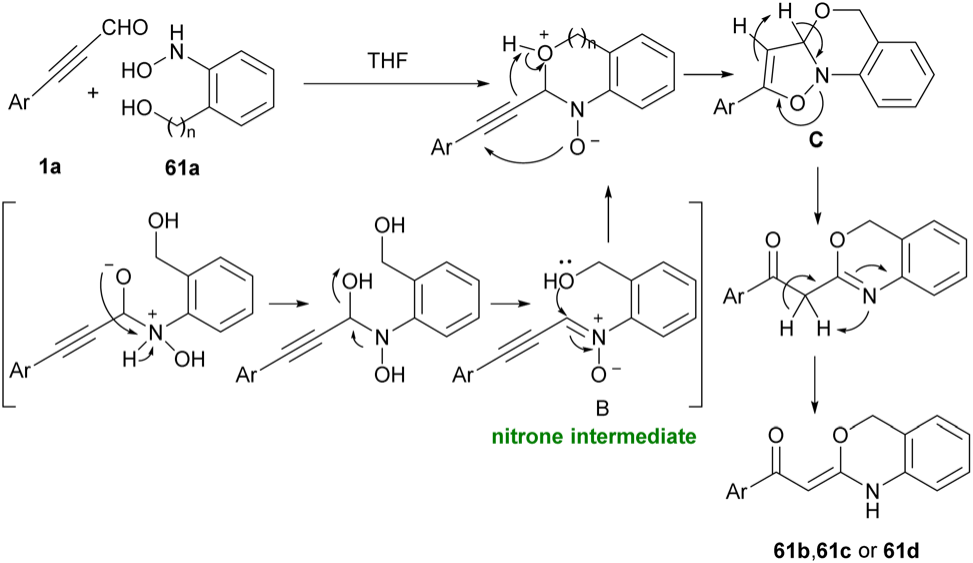
Possible mechanism for synthesis of benzo-oxazine, benzo-oxazepine and benzo-oxazocine.

Very recently, the synthesis of a series of six, seven, and eight-membered *N*,*O*-heterocyclic compounds, including benzo-oxazine 61b, benzo-oxazepine 61c and benzo-oxazocine 61d from easily available ynals 1a and 2-(hydroxyamino)phenyl alcohols 61a was explained by Dateer and co-workers (Scheme 84).^97^ Synthetic utility of benzo-oxazine was demonstrated by gram-scale synthesis and further conversion into the pyrazole derivative. A mechanism for this catalyst and additive-free method is illustrated in Scheme 85. The reaction commenced with the interaction of ynals 1a and 2-(hydroxyamino)phenyl alcohol 61a to generate nitrone intermediate A. Afterward, an intramolecular nucleophilic attack of OH on electron-deficient imine carbon and oxygen atom transfer to alkyne furnished dihydrobenzo[*d*]isoxazolol intermediate B. Finally, N–O bond cleavage, and rearrangement led to benzo-oxazine 61b, 61c, or 61d.

## 6. Synthesis of S, N-heterocyclic compounds

### 6.1. Synthesis of isothiazoles

**Scheme 86:**
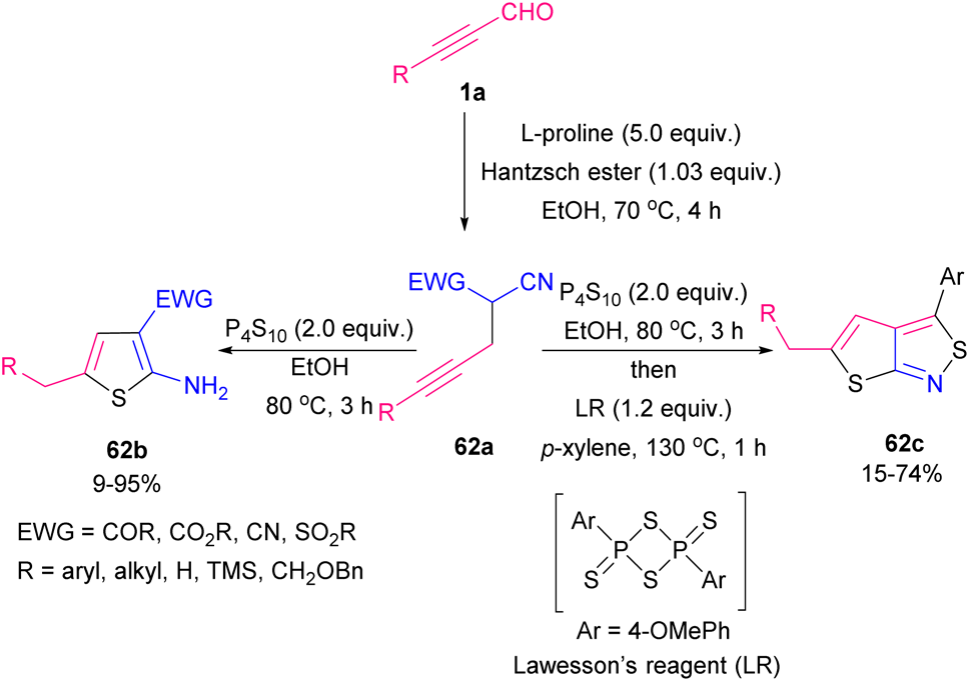
Synthesis of 2-aminothiophenes and thieno[2,3-*c*]isothiazoles.

**Scheme 87:**
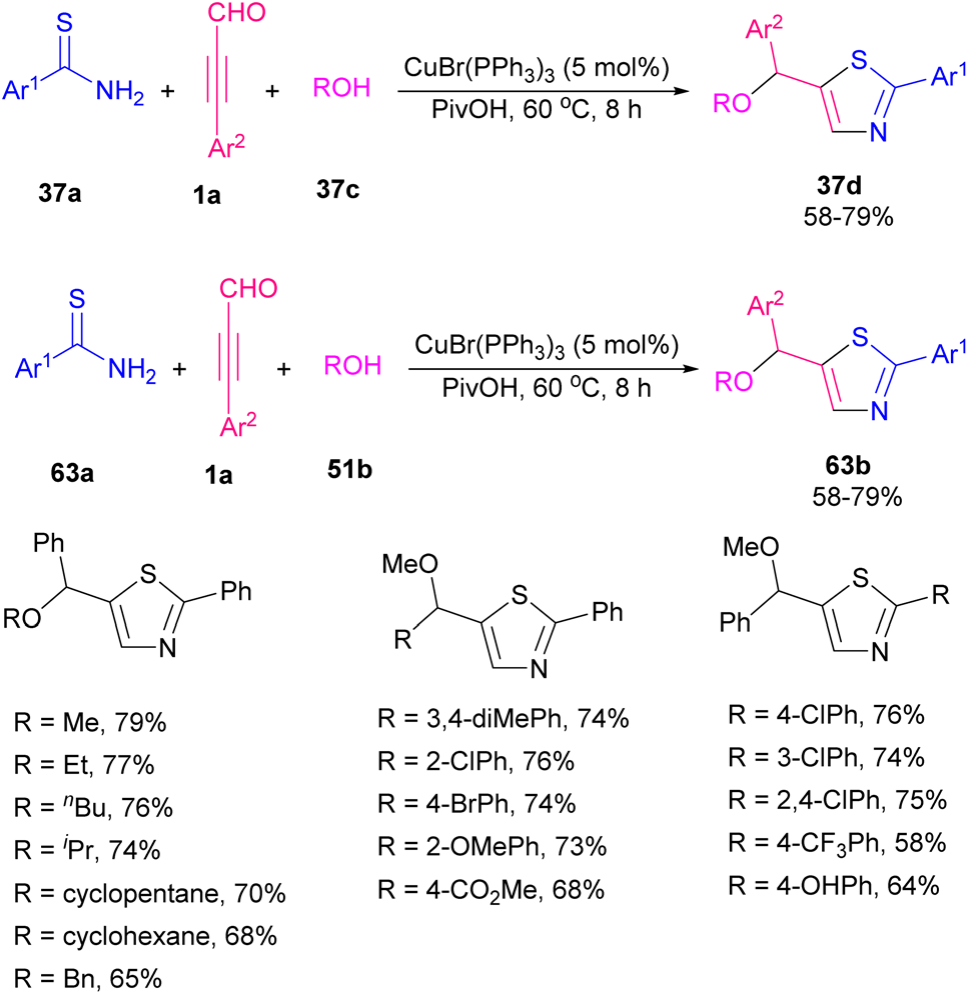
Cu-catalyzed construction of functionalized thiazoles.

An unprecedented temperature-controlled thiation of α-cyano-β-alkynyl carbonyl compounds 62a was explored by Shia and Wu *et al.* (Scheme 86).^98^ For this purpose, they examined phosphorus decasulfide and Lawesson's reagent as the sulfur-transferring reagents. In the case of 2-aminothiophene products 62b, the temperature was set at 80 °C in EtOH as the reaction solvent. When the temperature was enhanced to 130 °C, with the addition of *p*-xylene as the replaced solvent, thieno[2,3-*c*]isothiazole derivatives 62c were formed. It seems that the LR was responsible for the oxidation in the current procedure. Also, other thiation reagents like sodium sulfide and octasulfur did not effective. For producing functionalized thiazoles 63b*via* Cu-catalyzed C–N, C–O, and C–S bond formation, thioamides 63a, aryl alkynyl aldehydes 1a, and alcohols 51b were used (Scheme 87).^99^ Various aromatic and heteroaromatic groups in substrates tolerated well in such transformation and cyclized products achieved with good regioselectivity.

## 7. Conclusions

This comprehensive review has described the progress in synthesis processes to access N-, O-, and S-heterocycles from readily accessible alkynyl aldehydes by C–N/C–O/C–S/C–C bond formation. In this regard, a variety of transition metal-catalyzed, metal-free-promoted, and visible-light-mediated syntheses are highlighted in this concept. Challenging mechanistic insights as well as the scope of some substrate ranges showed. It is noted that NHC-catalyzed methodologies presented extensive potential applications compared to metal-catalyzed strategies that led to the green synthetic routes for constructing a vast number of N-, O-, and S-heterocycles. In our opinion, the design, and use of more simple NHC organocatalysts for alkynyl aldehyde reactions could strongly expand the scope of these synthetic applications. Despite recent progress, due to the importance of N-, O-, and S-heterocyclic frameworks in pharmaceutical and material chemistry, further developing new environmentally friendly and high atom-economic systems involving commercially available alkynyl aldehyde motifs is still required in this area. Also, it seems study on S-heterocyclic compounds from alkynyl aldehydes remain less explored in comparison to N-heterocycles. We hope that current review will open opportunities for medicinal and organic chemists to work in the field of N-, O-, and S-heterocycles synthesis.

## Conflicts of interest

There are no conflicts to declare.

## Supplementary Material
